# Supplementary material for: Nanoscale analysis of human G1 and metaphase chromatin in situ
Source: EMBO J. 2025 Mar 17;44(9):2658–94. doi: 10.1038/s44318-025-00407-2 (PMC12048539; doi:10.1038/s44318-025-00407-2)
Supplement: Supplementary file 1 — Appendix [file 44318_2025_407_MOESM1_ESM.pdf]

APPENDIX

Nanoscale analysis of human G1 and metaphase chromatin in situ

Jon Ken Chen, Tingsheng Liu (刘廷生), Shujun Cai (蔡舒君), Weimei Ruan, Cai Tong Ng, Jian Shi, Uttam Surana, and Lu Gan (甘露)*

* Correspondence: lu@anaphase.org

TABLE OF CONTENTS

Appendix Figures

Appendix Figure S1. Synchronization scheme. 3

Appendix Figure S2. Cryosections of RPE-1 cells in metaphase. 4

Appendix Figure S3. Screen of cryoprotectants in live cells. 5

Appendix Figure S4. Test of DMSO as a cryoprotectant for cryolamellae. 6

Appendix Figure S5. Tests of intracellular cryo-ET contrast in cryoprotectants. 7

Appendix Figure S6. Control cryo-ET of oligonucleosomes in DMSO cryoprotectant. 8

Appendix Figure S7. Classification of HeLa oligonucleosomes in 0% and 9% DMSO. 9

Appendix Figure S8. Cryoprotection treatments do not induce apoptosis markers. 10

Appendix Figure S9. Additional example Volta cryotomographic slice of a G1 cell. 11

Appendix Figure S10. Controls subtomogram analysis of ribosomes in situ. 13

Appendix Figure S11. Controls for nucleosome 3-D classification in situ. 14

Appendix Figure S12. Classification flowchart of G1 chromatin domains. 16

Appendix Figure S13. Refinement of G1 mononucleosomes and dinucleosomes. 17

Appendix Figure S14. Cryo-ET of G1 chromatin in situ of cells with glycerol cryoprotection. 19

Appendix Figure S15. Classification flowchart of G1 chromatin domains in 9% glycerol-cryoprotectant cells. 21

Appendix Figure S16. Refinement of mononucleosomes from glycerol-cryoprotected G1 cells. 22

Appendix Figure S17. Cryo-ET of G1 chromatin in situ of cells without cryoprotection. 24

Appendix Figure S18. Classification flowchart of G1 chromatin domains for cells without cryoprotection. 26

Appendix Figure S19. Refinement of Group 1 nucleosome class averages from G1 cells without cryoprotection. 27

Appendix Figure S20. Additional CNN annotations of chromatin domains in G1 nuclei. 29

Appendix Figure S21. Remapped models of G1 nucleosome groups. 30

Appendix Figure S22. Remapped models of G1 nucleosome individual classes. 31

Appendix Figure S23. Subtomogram analysis of the G1 nucleoplasm. 33

Appendix Figure S24. Targeting images for chromatin in metaphase cells. 34

Appendix Figure S25. Additional example Volta cryotomographic slice of a metaphase cell. 35

Appendix Figure S26. Classification flowchart of M cells. 38

Appendix Figure S27. Refinement of metaphase mononucleosomes and dinucleosomes. 39

Appendix Figure S28. Remapped models of metaphase nucleosome groups. 40

Appendix Figure S29. Remapped models of metaphase nucleosome individual classes. 41
Appendix Figure S30. Refinement of Group 1 mononucleosomes combined into two classes. 42
Appendix Figure S31. Uncropped immunoblots. 44
Appendix Figure S32. Non-denoised version of Fig 2. 45
Appendix Figure S33. Reproduction of Fig 3, but with non-denoised versions of the cryotomographic slices. 46
Appendix Figure S34. Non-denoised version of Fig 4. 47
Appendix Figure S35. Reproduction of Fig 5, but with non-denoised versions of the cryotomographic slices. 48
Appendix Figure S36. Reproduction of Fig 6, but with non-denoised versions of the cryotomographic slices. 49

Appendix Tables

Appendix Table S1. Research resources. 50
Appendix Table S2. Antibodies used. 52
Appendix Table S3. Confocal microscopy details. 53
Appendix Table S4. Cryo-ET imaging details. 54
Appendix Table S5. Cryotomogram details. 56
Appendix Table S6. Subtomogram analysis of chromatin. 57
Appendix Table S7. Subtomogram analysis of chromatin – nucleosomes with gyre-proximal densities. 58
Appendix Table S8. Subtomogram analysis of G1 chromatin with 9% glycerol or without cryoprotectant. 59
Appendix Table S9. Subtomogram analysis of megacomplexes. 60

Appendix Discussion 61

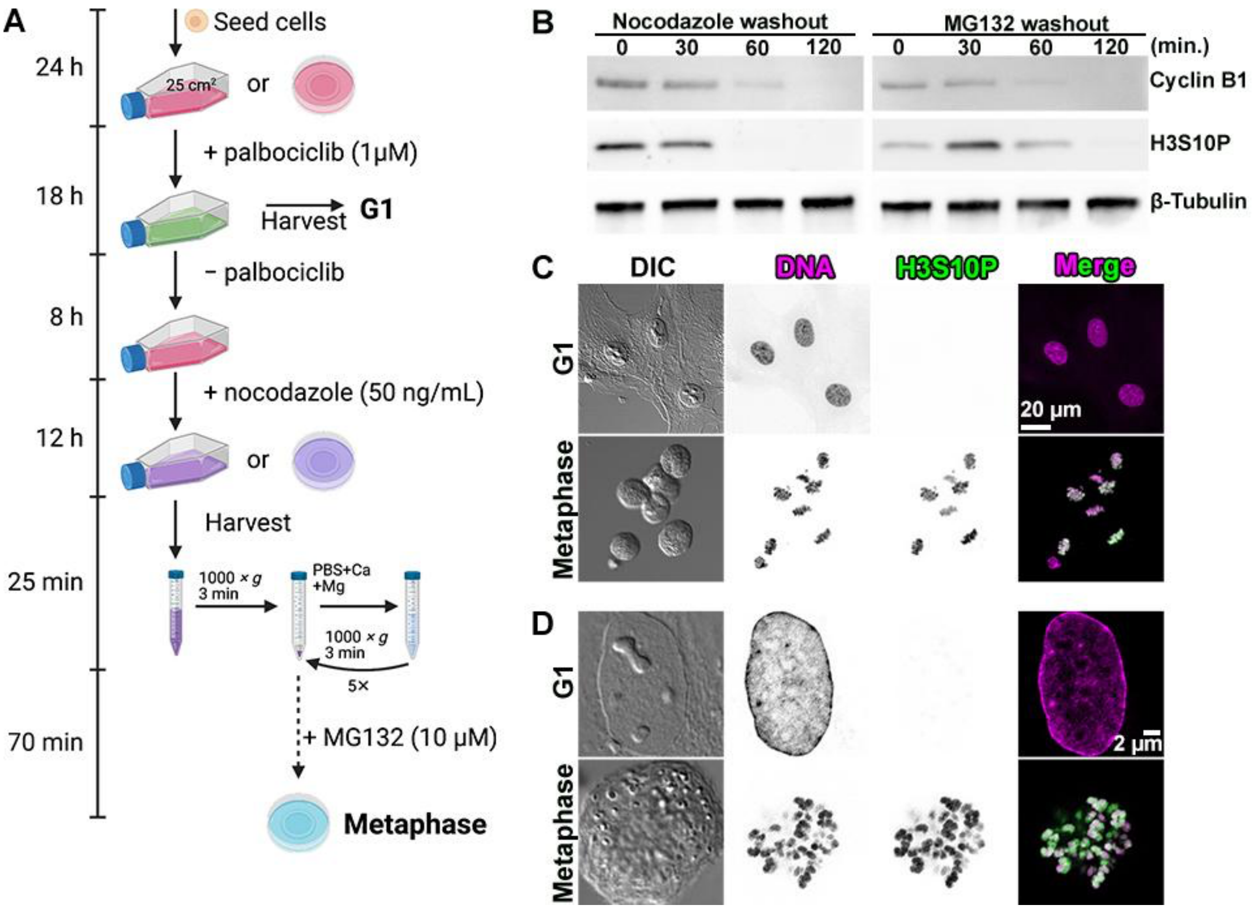

Appendix Figure S1. Synchronization scheme.

(A) Schematic of RPE-1 G1 and metaphase cell-cycle synchronization. Created with BioRender.com. (B) Immunoblot analysis showing decrease of mitotic markers cyclin B1 and histone H3 phosphorylated at serine 10 (H3S10P) 0 – 120 minutes following washout of nocodazole and MG132. β-Tubulin is the loading control. The uncropped blots are shown in Appendix Fig S31. (C) Differential interference contrast (DIC) and immunofluorescence images of G1 and metaphase cells. The chromatin is stained with DAPI (DNA). Immunostaining for histone H3 phosphorylated at serine 10 (H3S10P). The fluorescence contrast is inverted for better visibility. (D) Airyscan sections of a G1 and a metaphase cell, showing the localization of H3S10P in the metaphase chromosomes.

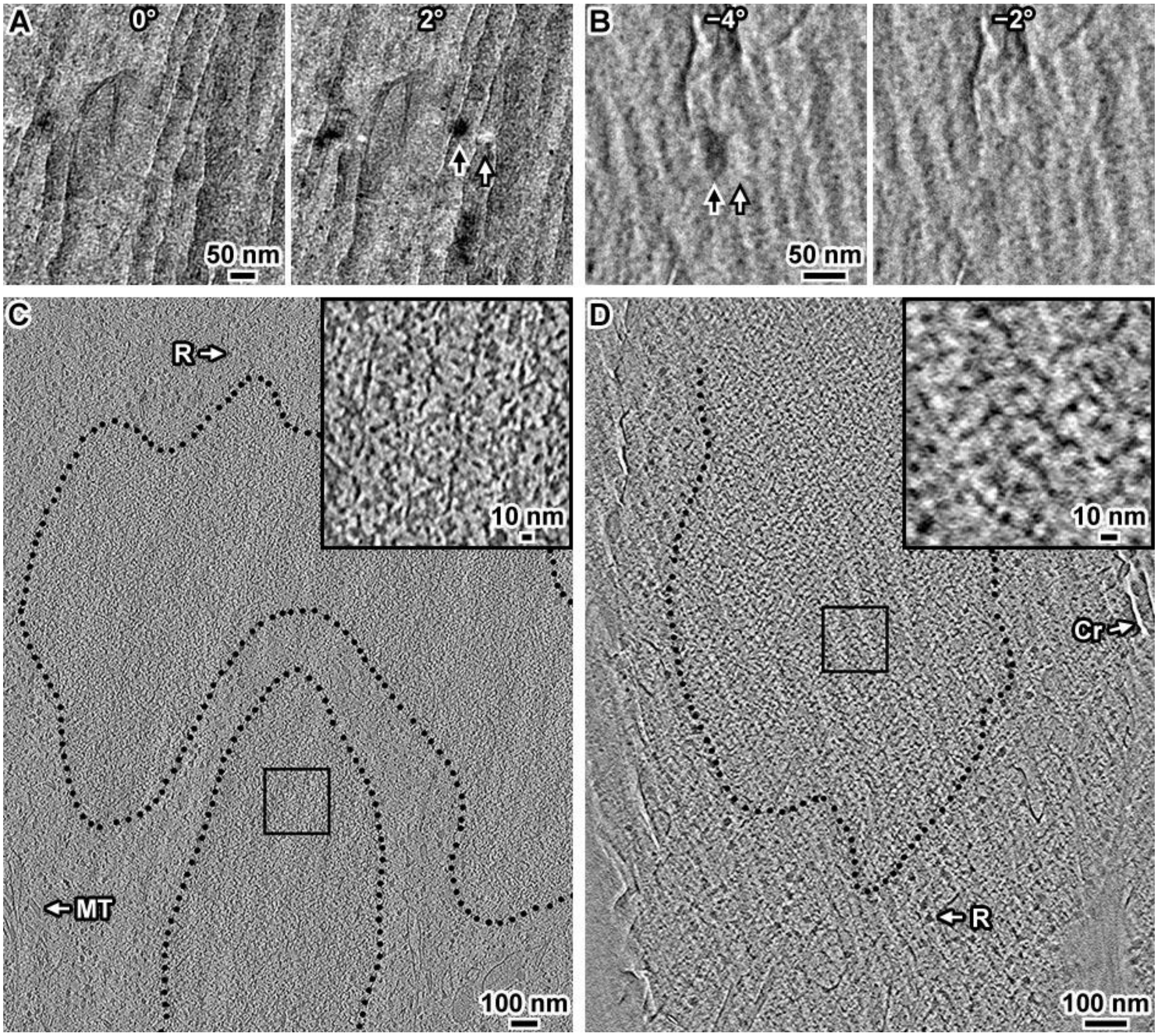

Appendix Figure S2. Cryosections of RPE-1 cells in metaphase.
(A and B) Pairs of tilt series images of two RPE-1 cells. In each panel, the two images correspond to a 2° difference in tilt angle. The black and white arrows indicated diffraction contrast features that arise from crystalline ice within the cellular cryosection. The linear vertical features are crevasses. (C and D) Cryotomographic slices (12 nm) of the cells in panels A and B, respective. Cytological features such as ribosomes (R) and a microtubule (MT) are indicated. (Cr) indicates a crevasse feature. Black dotted lines indicate the approximate boundary that encloses the compacted chromosomes. The insets show 4-fold enlargements of the boxed areas in the chromosomes.

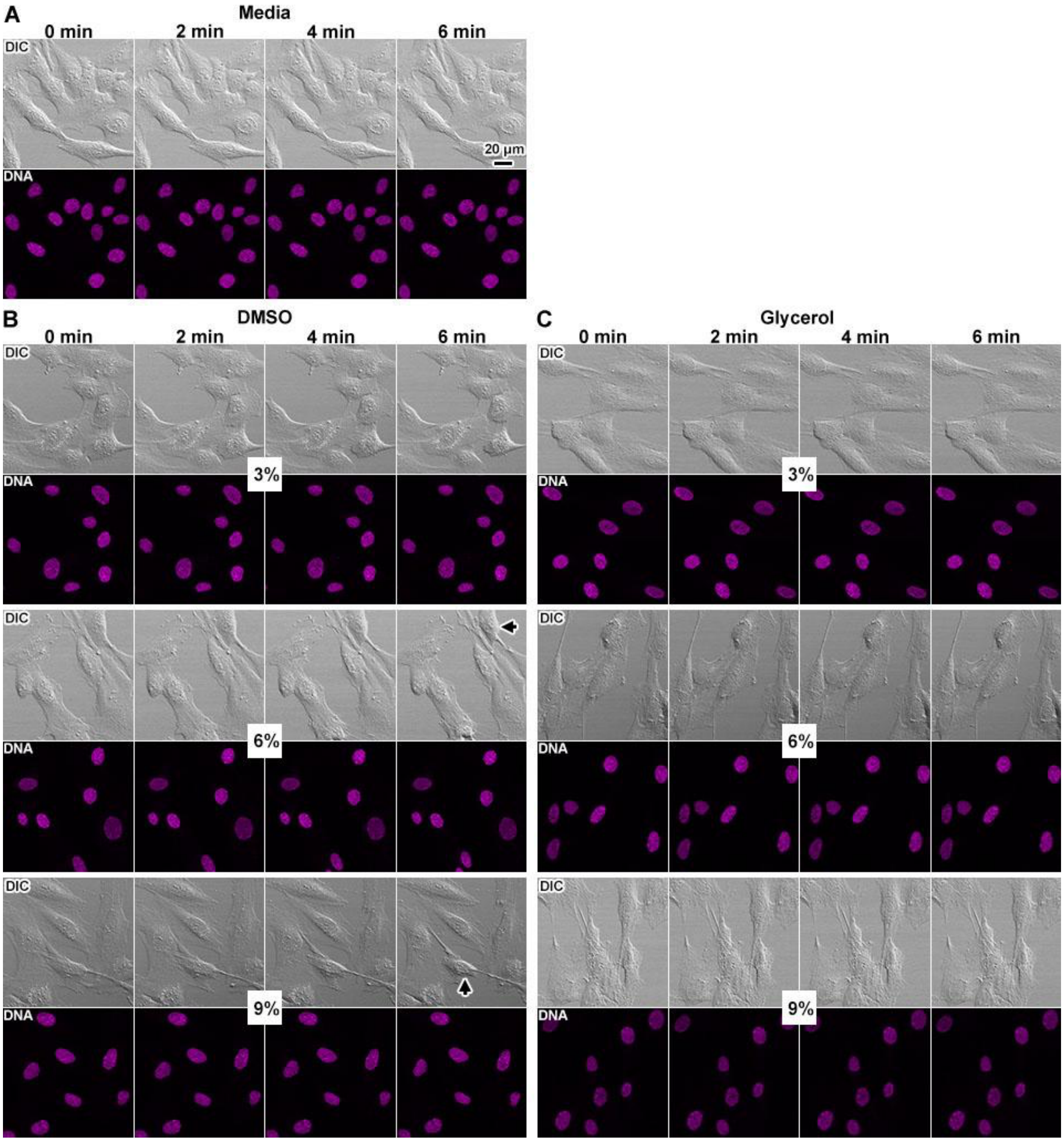

Appendix Figure S3. Screen of cryoprotectants in live cells.
(A) Control timelapse images of unsynchronized RPE-1 cells in complete media and stained with Hoechst 33342, imaged every 2 minutes for 6 minutes. Images were acquired in the DIC (upper row) and Hoechst 33342 (lower row) channels. The experimental delay between the addition of cryoprotectant and the acquisition of the first image is estimated to be 1 minute. Timelapse imaging was done for cells in either (B) DMSO or (C) glycerol, at three different concentrations (3%, 6%, and 9%). The short arrows in panel B indicate cells that have started to detach.

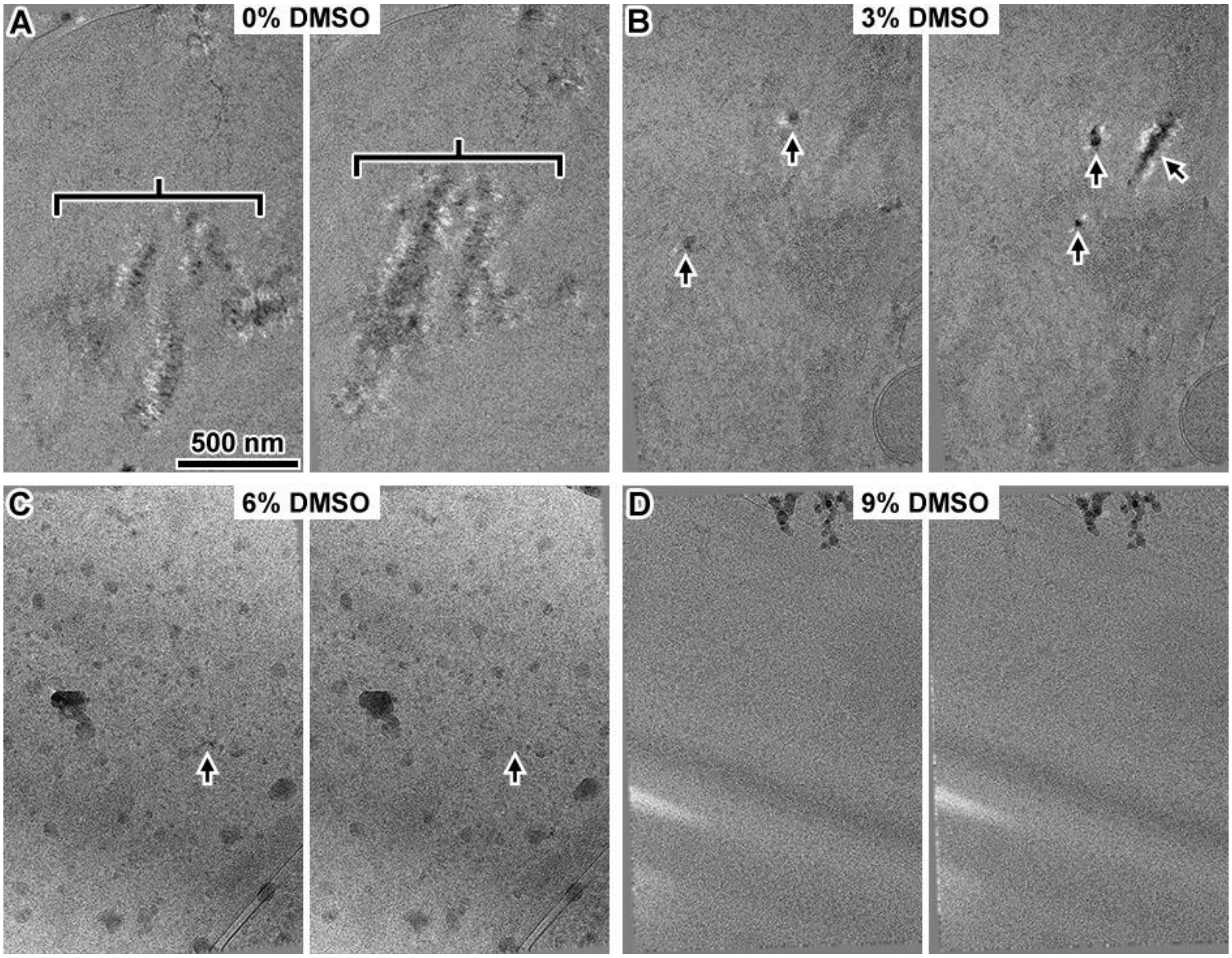

Appendix Figure S4. Test of DMSO as a cryoprotectant for cryolamellae.
Example G1-arrested RPE-1 cells, grown on EM grids, were briefly dipped in PBS+Ca+Mg supplemented with (A) 0%, (B) 3%, (C) 6%, or (D) 9% DMSO. The cells were then plunge-frozen, cryo-FIB milled, and imaged as a tilt series. To ensure that all four freezing conditions were tested on samples of similar thickness, the tilt series were collected at the nuclear periphery. Distinct nuclear double membranes (nuclear envelope) were observed for all tilt series, indicating that the sampled heights were indeed at the nuclear periphery. Note that the double membranes are not clear in panels B and D but are visible in the reconstructed cryotomograms. For each image pair, the tilt angle differed by 2°. The ice-crystal diffraction-contrast features are indicated by the brackets in panel A and short arrows in panel B. In panel C, the dense ~100- to 200-nm rounded features are ice-crystal contaminants on the lamella's surface. In panel D, the bright and dark streaks oriented 10 to 4 o'clock are from uneven milling.

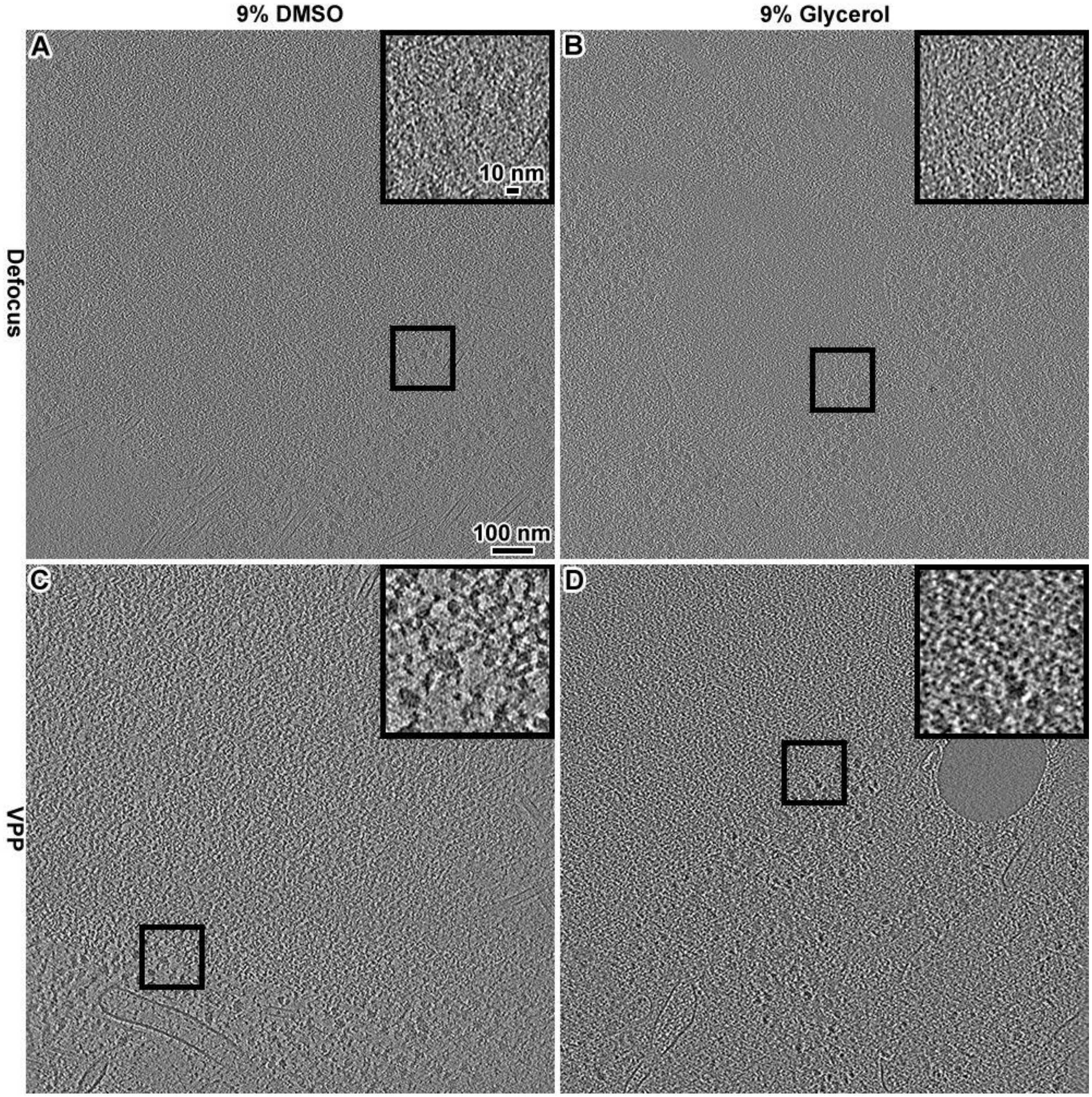

Appendix Figure S5. Tests of intracellular cryo-ET contrast in cryoprotectants. Cryotomographic slices (12 nm) of metaphase-arrested RPE-1 cells frozen in (A) 9% DMSO and imaged by defocus phase contrast (Defocus), (B) 9% Glycerol and imaged by defocus phase contrast, (C) 9% DMSO and imaged with a Volta phase plate (VPP), (D) 9% Glycerol and imaged with a VPP. The fitted defocus was −5 μm in panels A and B and the nominal defocus was −0.5 μm in panels C and D. Insets show 3-fold enlargements of the corresponding boxed areas. A region that has both ribosomes and chromatin was chosen for each inset.

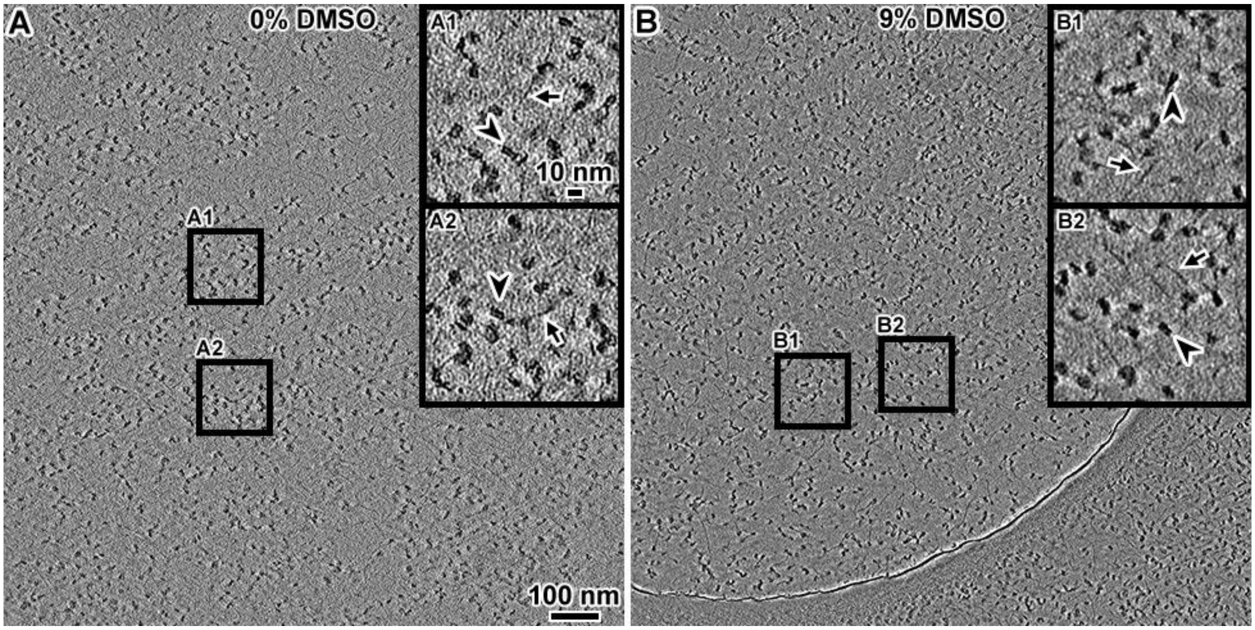

Appendix Figure S6. Control cryo-ET of oligonucleosomes in DMSO cryoprotectant. Volta cryotomographic slices (10 nm) of HeLa oligonucleosomes in (A) storage buffer and (B) storage buffer plus 9% v/v DMSO. The granular densities are the nucleosomes. The large arc-shaped feature in the lower portion of panel B is the edge of the holey-carbon support film. The insets show 3-fold enlargements of boxed regions. Stretches of naked DNA (short arrows) and nucleosome double-gyre motifs (arrowheads) are indicated.

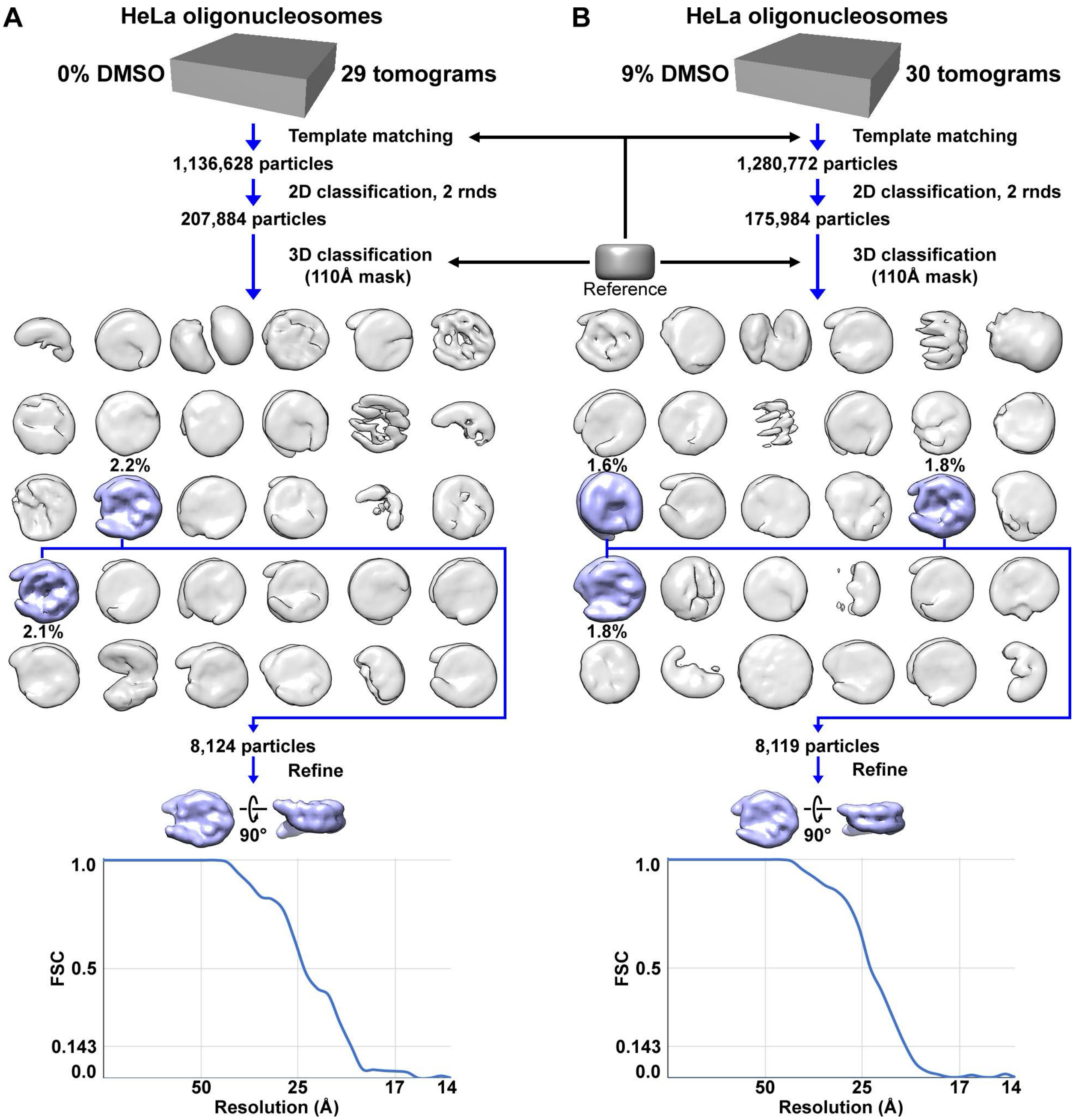

Appendix Figure S7. Classification of HeLa oligonucleosomes in 0% and 9% DMSO. Classification and subtomogram analysis of oligonucleosomes in (A) 0% DMSO control and (B) 9% DMSO. Nucleosome-like particles were template matched using a 10-nm-wide, 6-nm-thick smooth cylindrical reference. Then two sequential rounds of 2-D classification were performed. One round of 3-D classification using k = 30 classes was done. The canonical nucleosomes with the higher-resolution features (those that didn't have smooth surfaces) were combined for 3-D refinement. Based on the Fourier shell correlation (FSC) = 0.5 cutoff criterion, the resolution of the 0% and 9%-DMSO-treated oligonucleosomes class averages were 19 Å and 19.5 Å, respectively.

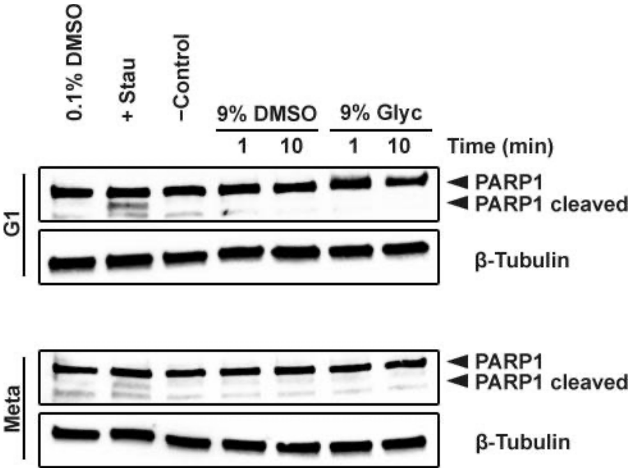

Appendix Figure S8. Cryoprotection treatments do not induce apoptosis markers.
Rows 1+2 and 3-4: Immunoblots of RPE-1 G1 and metaphase (Meta) cells, respectively. Rows 1 and 3 were probed for PARP1 while rows 2 and 4 were probed for beta-tubulin (loading control). The cleaved PARP1 band is found in apoptotic cells. Lanes 1 and 2: Apoptosis positive control in which RPE1 cells were treated with either carrier (0.1% DMSO) or 1 μM staurosporine (Stau) in 0.1% DMSO for 6 hours. Lane 3: untreated RPE-1 cells (negative control). Lanes 4 and 5: RPE-1 cells treated for 1 or 10 minutes with 9% DMSO. Lanes 6 and 7: RPE-1 cells treated for 1 or 10 minutes with 9% glycerol (Glyc). The uncropped blots are shown in Appendix Fig S31, C and D.

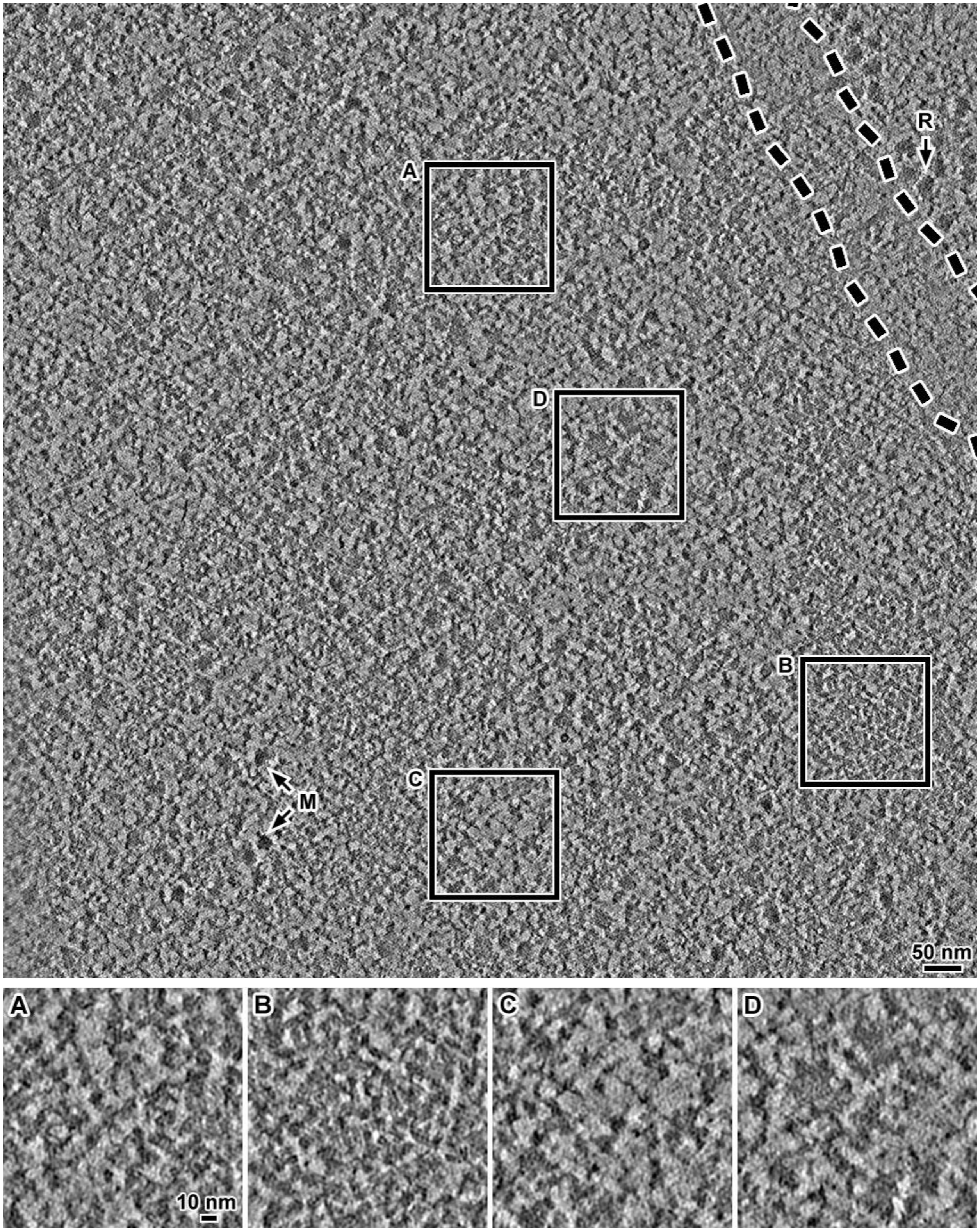

Appendix Figure S9. Additional example Volta cryotomographic slice of a G1 cell. Cryotomographic slice (20 nm) of nuclear densities centered near the nuclear envelope of an RPE-1 cell. Rendered with low JPEG compression. Insets show 2-fold enlargements of (A and B) portions of chromatin domains, (C) region with fewer macromolecular complexes, and (D) a

region with many megacomplexes. Cytological features are highlighted: megacomplex (M); ribosome (R). The region between the two dashed lines is the lumen of the nuclear envelope, which is oriented obliquely to the milling direction. As such, the nuclear envelope membranes are not visible.

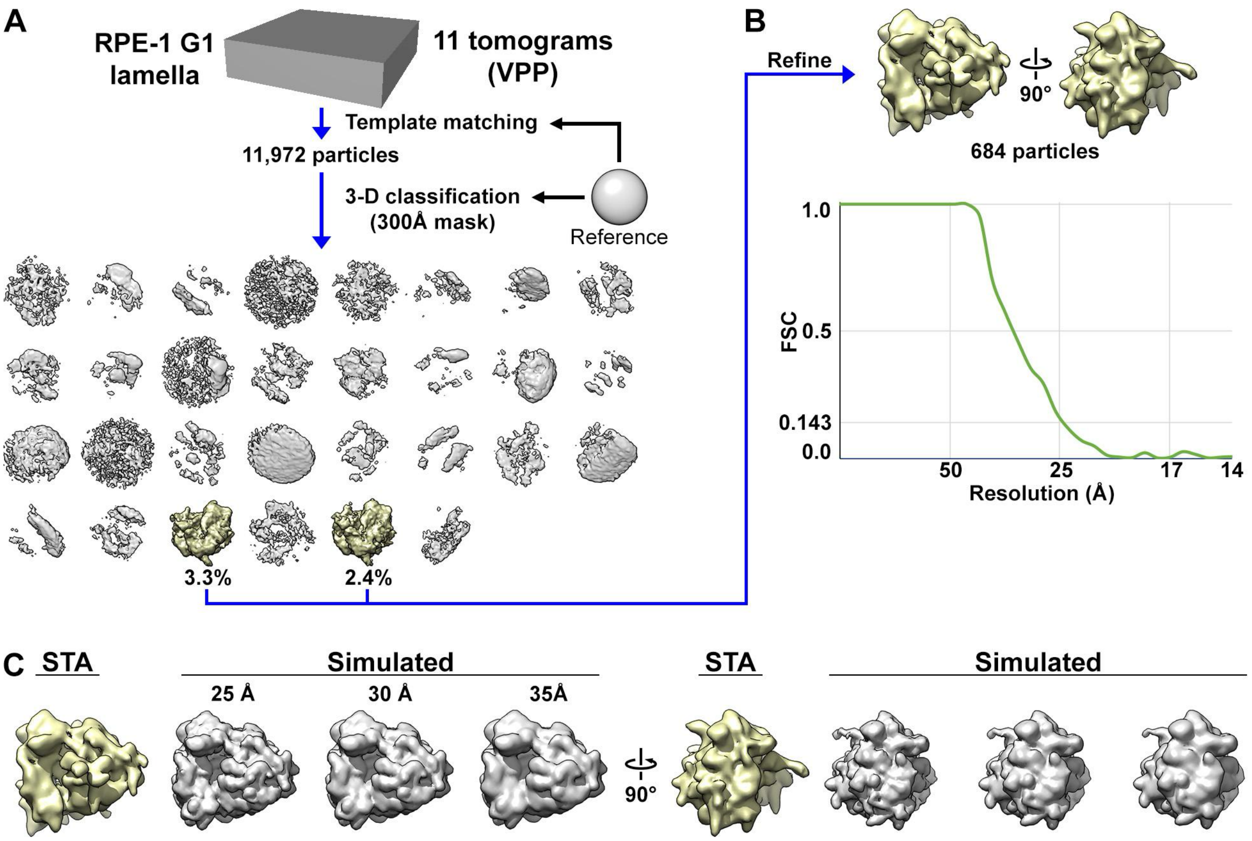

Appendix Figure S10. Controls subtomogram analysis of ribosomes in situ.
(A) Template matching was done only on the cytoplasmic regions, using a spherical reference. The template-matching hits were then subjected to direct 3-D classification (also using a spherical reference), revealing numerous non-ribosome class averages (gray) and two 80S ribosome class averages (yellow). (B) The particles of these two class averages were combined and refined to ~33 Å resolution, based on the FSC = 0.5 criterion. (C) The (yellow) refined *in situ* 80S ribosome density map reproduced from panel B, alongside (gray) density maps of purified human ribosomes (4UG0) (Khatter *et al*, 2015) simulated at different resolutions.

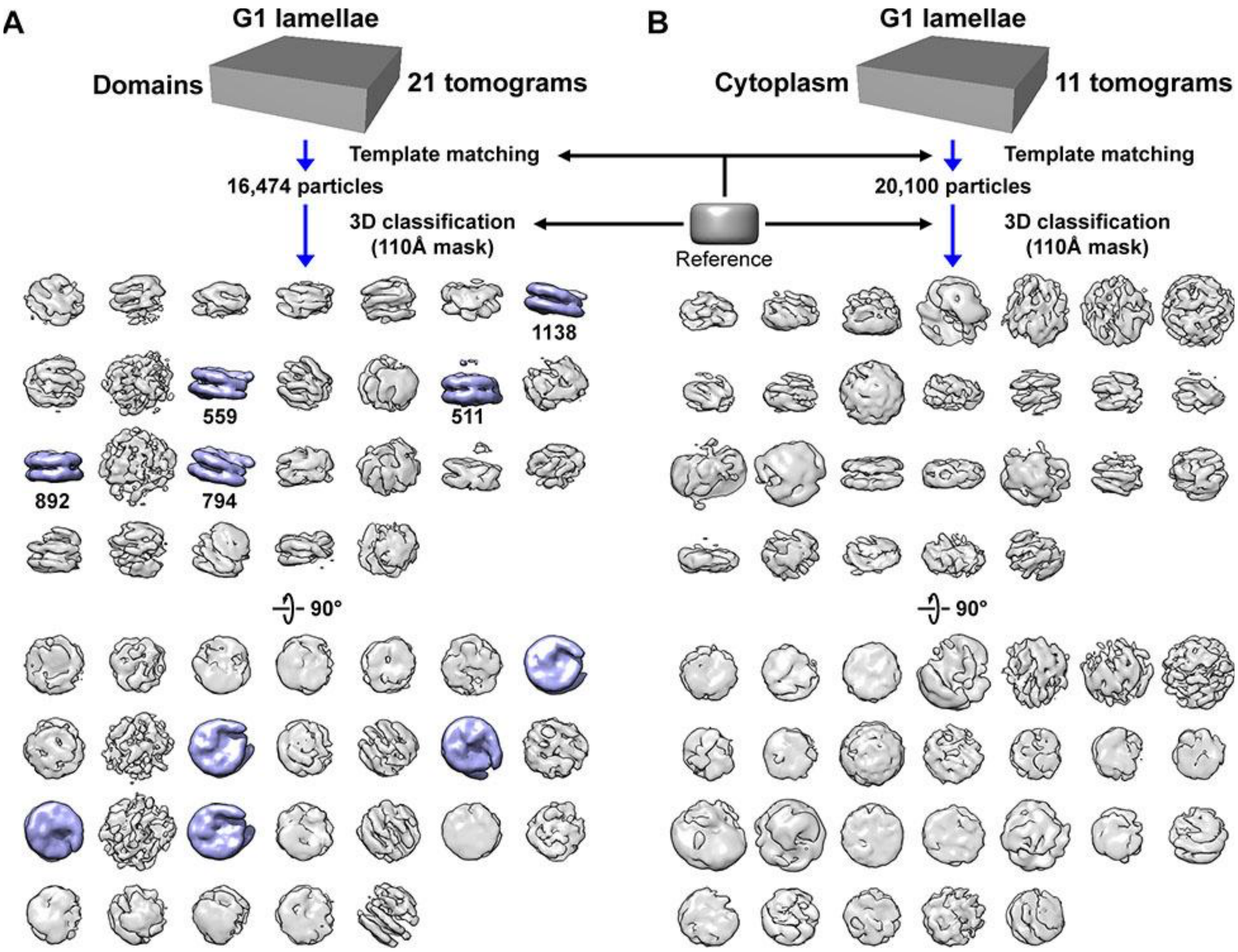

Appendix Figure S11. Controls for nucleosome 3-D classification in situ.
(A) Subtomogram analysis of G1 chromatin domains. (B) Subtomogram analysis of G1 cytoplasm. Template matching was done with a featureless cylinder reference and a 110 Å spherical mask. The grid spacing for both template matching experiments was 21 nm. The same reference and mask were used for both datasets. Canonical nucleosome class averages are shaded in blue while ambiguous class averages are shaded in gray. The ambiguous densities (gray) are not canonical nucleosomes; they are abundant because the template-matching process uses a featureless cylinder reference and a low cross-correlation cutoff. As a result, large numbers of false positives are rejected in the classification analysis. Classification was done using 30 classes for each experiment. Some classes had too few particles and were therefore not shown.

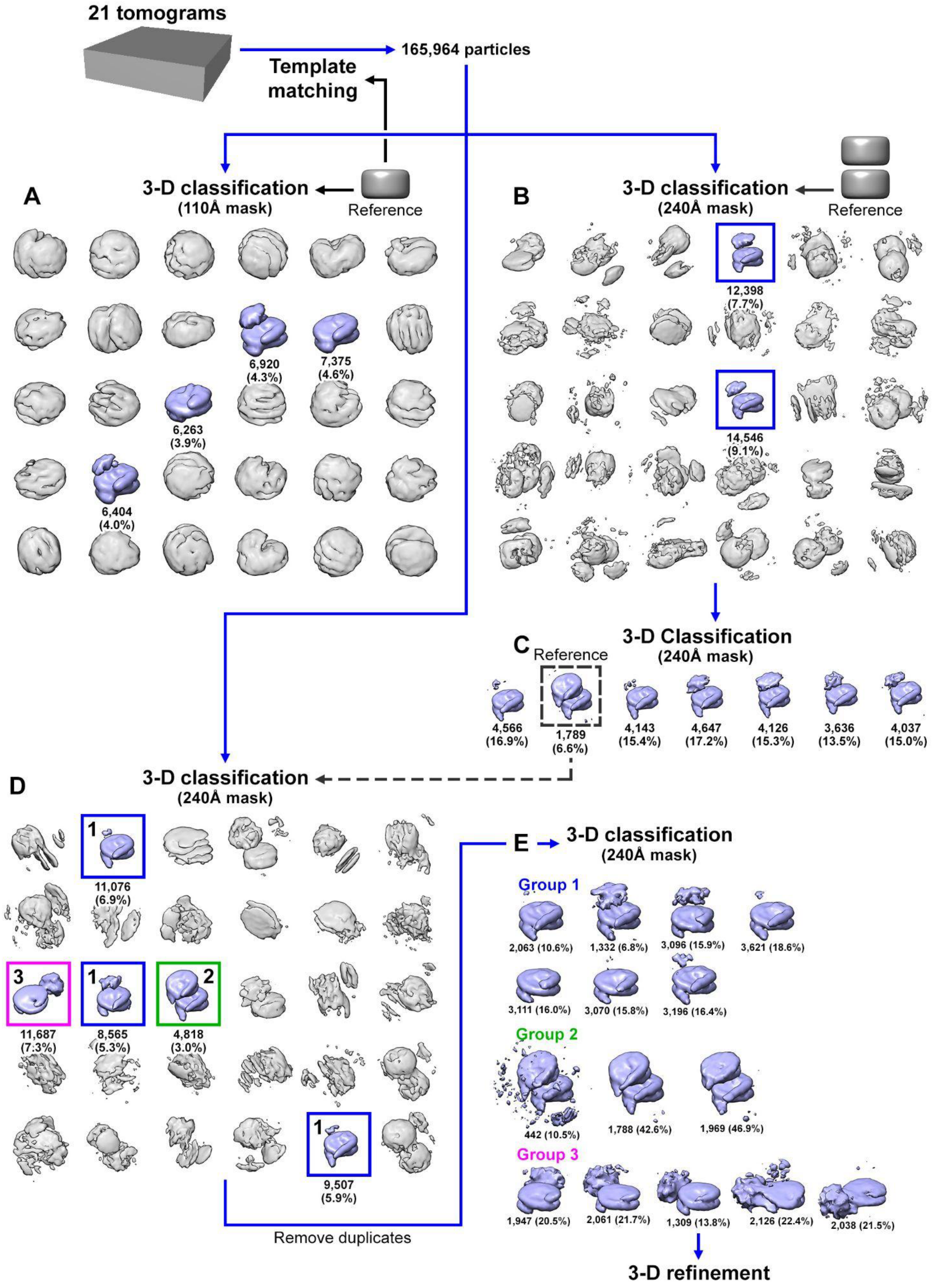
21 tomograms
165,964 particles
Template matching
A
3-D classification
(110Å mask)
Reference
6,920
(4.3%)
7,375
(4.6%)
6,263
(3.9%)
6,404
(4.0%)
B
3-D classification
(240Å mask)
Reference
12,398
(7.7%)
14,546
(9.1%)
C
Reference
3-D Classification
(240Å mask)
4,566
(16.9%)
1,789
(6.6%)
4,143
(15.4%)
4,647
(17.2%)
4,126
(15.3%)
3,636
(13.5%)
4,037
(15.0%)
D
3-D classification
(240Å mask)
1
11,076
(6.9%)
3
1
2
11,687
(7.3%)
8,565
(5.3%)
4,818
(3.0%)
1
9,507
(5.9%)
Remove duplicates
E
3-D classification
(240Å mask)
Group 1
2,063 (10.6%)
1,332 (6.8%)
3,096 (15.9%)
3,621 (18.6%)
3,111 (16.0%)
3,070 (15.8%)
3,196 (16.4%)
Group 2
442 (10.5%)
1,788 (42.6%)
1,969 (46.9%)
Group 3
1,947 (20.5%)
2,061 (21.7%)
1,309 (13.8%)
2,126 (22.4%)
2,038 (21.5%)
3-D refinement

Appendix Figure S12. Classification flowchart of G1 chromatin domains.
(A) G1 candidate nucleosome subtomograms that were template matched with a cylindrical reference were directly classified in 3-D, using a cylindrical reference and a 110 Å spherical mask. Note that there are more template-matching hits in this set of experiments than that of Appendix Fig S11 because a smaller grid spacing was used here. The ambiguous densities (gray) are not canonical nucleosomes; they are abundant because the template matching process uses a featureless cylinder reference and a low cross-correlation cutoff. As a result, large numbers of false positives are rejected in the classification analysis. (B) In parallel, the same set of subtomograms were directly classified in 3-D using a stacked cylinder reference and a larger spherical mask. (C) A second round of classification using a nucleosome class average from panel B as the reference and the larger mask yielded mononucleosomes with extra densities at their face plus an unambiguous dinucleosome class. (D) Direct 3-D classification was done on the original set of subtomograms using a larger mask and the stacked dinucleosome class average from panel C as the reference. Three groups of class averages were obtained, corresponding to (1) mononucleosome, (2) stacked dinucleosome, and (3) mononucleosome with a gyre-proximal density. (E) These three groups of class averages were subjected to a third round of classification using either a mononucleosome, stacked dinucleosome or mononucleosome with a gyre-proximal density from panel D as the reference and large spherical mask.

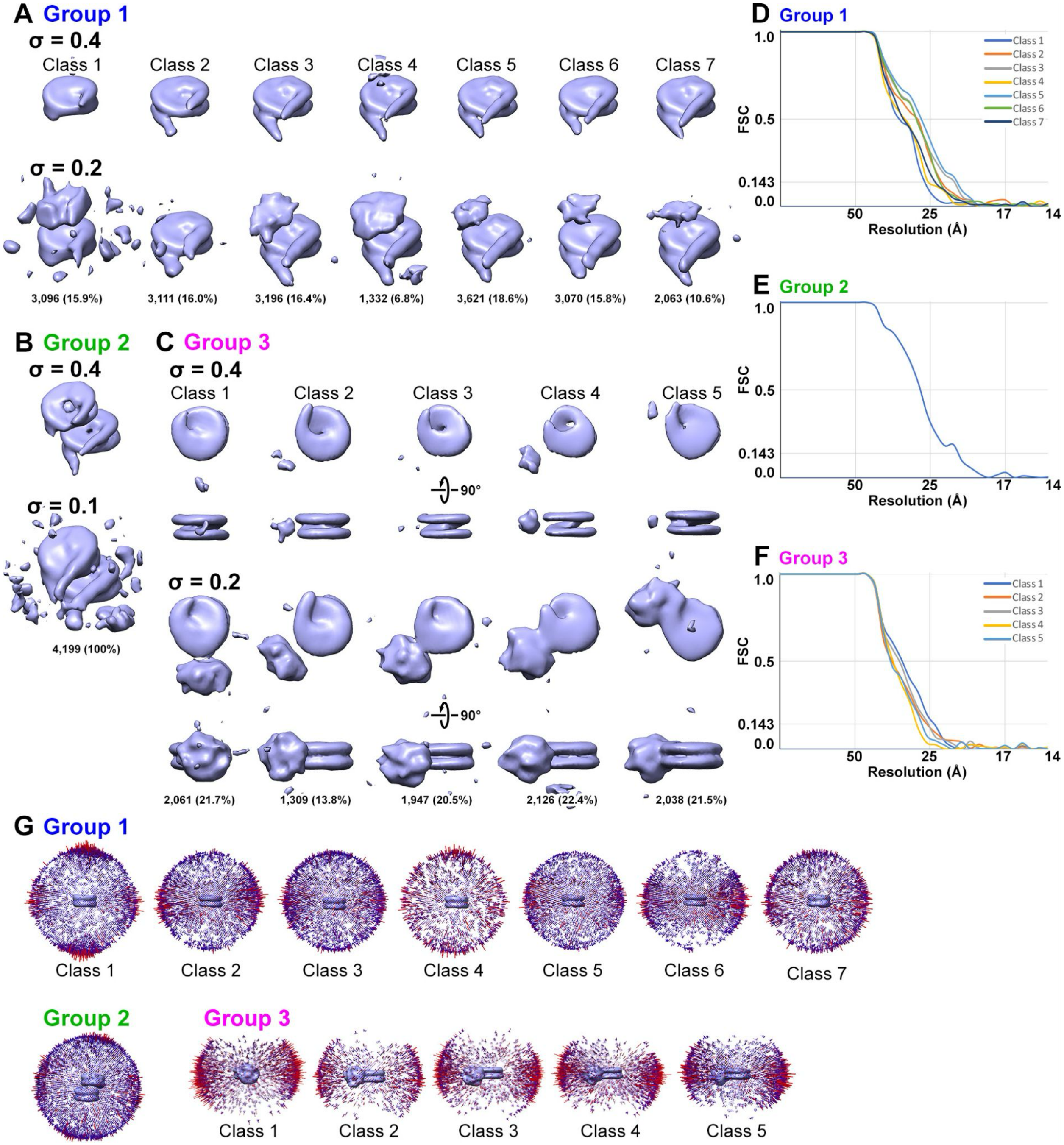

Appendix Figure S13. Refinement of G1 mononucleosomes and dinucleosomes. Refined class averages for (A) mononucleosomes, (B) stacked dinucleosomes, and (C) mononucleosomes with a gyre-proximal density. Two contour levels are shown for each class average. (D, E, F) FSC plots and (G) angular distribution of the refined density maps. For panels A – C, the hide dust feature in UCSF Chimera was disabled.

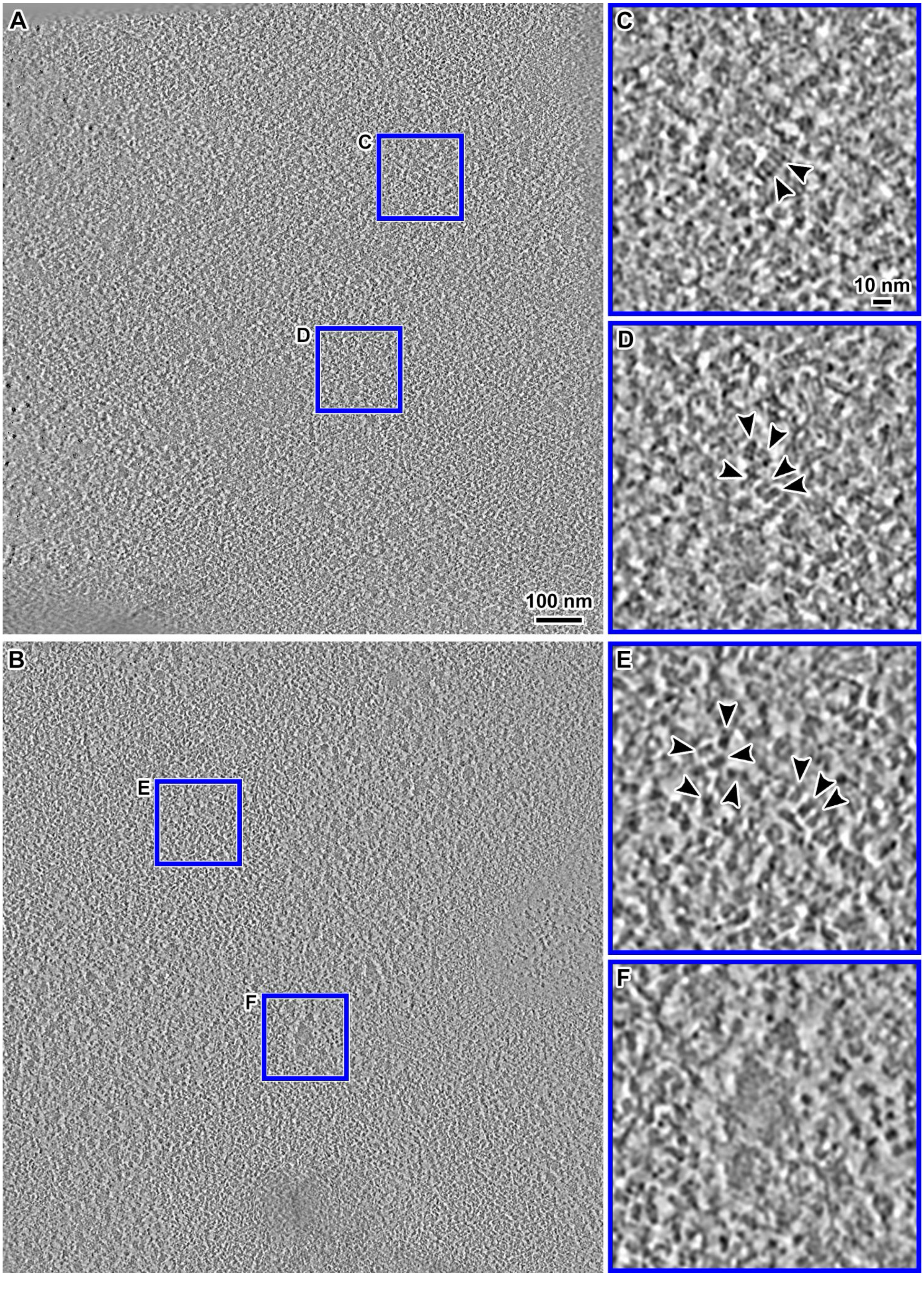
A
C
D
100 nm
B
E
F
C
10 nm
D
E
F

Appendix Figure S14. Cryo-ET of G1 chromatin in situ of cells with glycerol cryoprotection.

(A & B) Cryotomographic slices (10 nm) of the nuclear region of G1 RPE-1 cells that were plunge frozen in the presence of 9% glycerol for cryoprotection. (C & D) Enlargements (4-fold) of the two boxed regions in panel A, showing chromatin domains. (E) Enlargement (4-fold) of another chromatin domain in Panel B. (F) Enlargement (4-fold) of a “dense irregular body” in panel B. Nucleosomes are indicated by black arrowheads in panels C, D and E. These images are from cryotomograms denoised by CryoCARE.

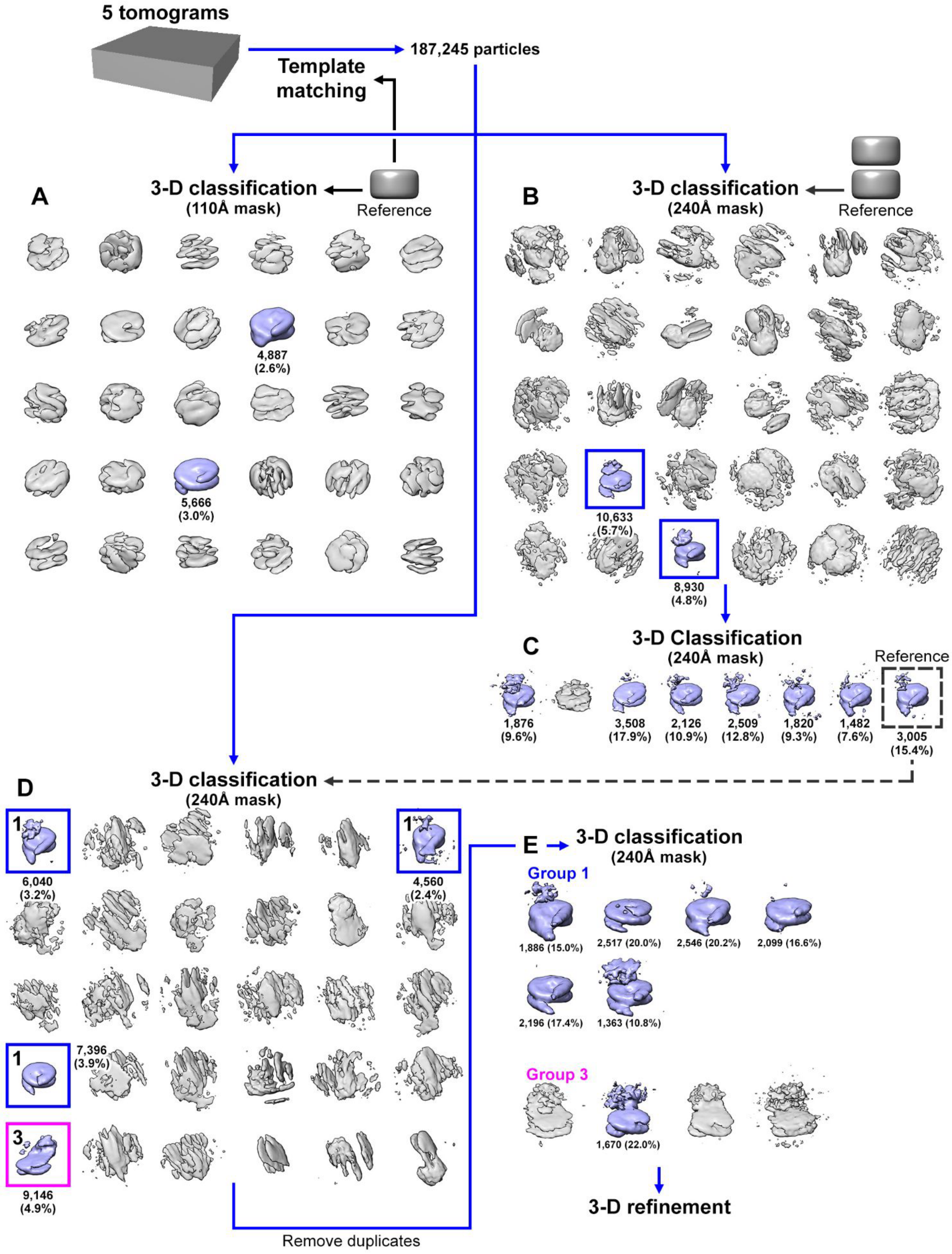
5 tomograms
187,245 particles
Template matching
A
3-D classification
(110Å mask)
Reference
4,887
(2.6%)
5,666
(3.0%)
B
3-D classification
(240Å mask)
Reference
10,633
(5.7%)
8,930
(4.8%)
C
3-D Classification
(240Å mask)
Reference
1,876
(9.6%)
3,508
(17.9%)
2,126
(10.9%)
2,509
(12.8%)
1,820
(9.3%)
1,482
(7.6%)
3,005
(15.4%)
D
3-D classification
(240Å mask)
1
6,040
(3.2%)
1
4,560
(2.4%)
1
7,396
(3.9%)
3
9,146
(4.9%)
Remove duplicates
E
3-D classification
(240Å mask)
Group 1
1,886 (15.0%)
2,517 (20.0%)
2,546 (20.2%)
2,099 (16.6%)
2,196 (17.4%)
1,363 (10.8%)
Group 3
1,670 (22.0%)
3-D refinement

Appendix Figure S15. Classification flowchart of G1 chromatin domains in 9% glycerol-cryoprotectant cells.

The same classification workflow used in the analysis of the 9% DMSO-cryoprotected cell dataset (Appendix Fig S12) is used here. Briefly, tomograms collected from 9% glycerol-cryoprotected G1 cells were template matched using a featureless cylindrical reference. (A) The resultant candidate nucleosome subtomograms were then classified in 3-D, using the cylinder reference and a 110 Å spherical mask. (B) In parallel, the same set of candidate subtomograms were also classified using a stacked cylinder reference and a larger 240 Å spherical mask. (C) Nucleosome class averages detected in panel B were then subjected to a second round of classification, using one of the class averages as the reference. Unlike in the tomograms of 9% DMSO-cryoprotected cells (Appendix Fig S14C), the stacked dinucleosome class average was not detected here. (D) The original set of the candidate subtomograms was classified again, this time using a mononucleosome class average from panel C as the reference. Two groups of mononucleosome class averages were obtained, corresponding to (Group 1, boxed blue) mononucleosomes and (Group 3, boxed magenta) mononucleosomes with a proximal-gyre density. (E) The two groups were then subjected to a third round of classification separately, using either a mononucleosome or a mononucleosome with proximal-gyre density class average from panel D as the reference. The nucleosome class averages in panel E were then individually subjected to refinement (see Appendix Fig S16). Nucleosome class averages are colored blue; ambiguous class averages are colored gray.

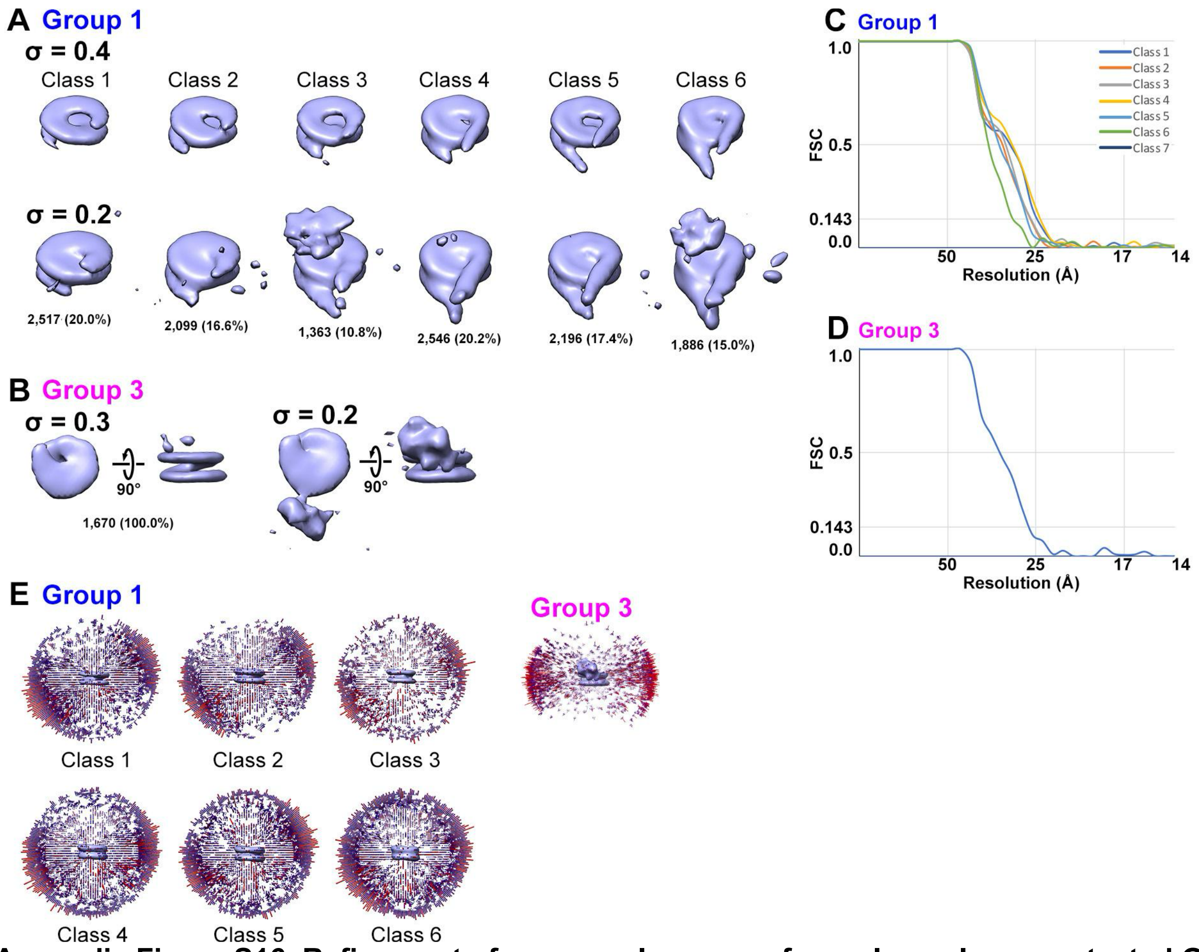

Appendix Figure S16. Refinement of mononucleosomes from glycerol-cryoprotected G1 cells.

Refined class averages for (A) mononucleosomes and (B) mononucleosome with a proximal-gyre density. Two contour levels are shown for each class average. (C & D) FSC plots and (E) angular distributions of the refined class averages in panels A and B.

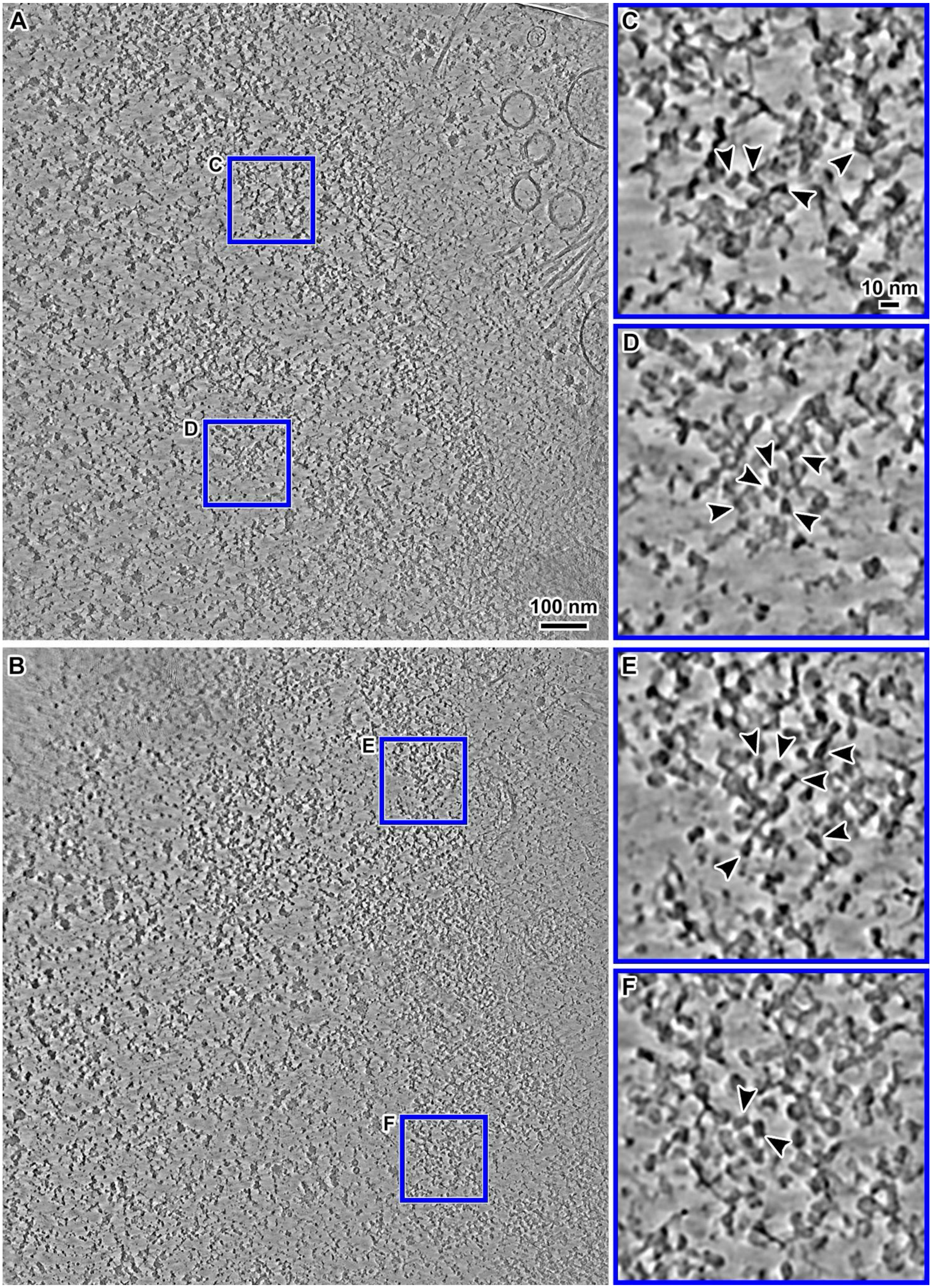
A
C
D
100 nm
B
E
F
C
10 nm
D
E
F

Appendix Figure S17. Cryo-ET of G1 chromatin in situ of cells without cryoprotection. (A & B) Cryotomographic slices (10 nm) of perinuclear regions of G1 RPE-1 cells that were plunge frozen without addition of a cryoprotectant. The nuclear envelopes are indicated by the dashed lines. (C & D) Enlargements (4-fold) of chromatin domains in Panel A. (E & F) Enlargements (4-fold) of chromatin domains in panel B. Nucleosomes in panels C – F are indicated by black arrowheads. These images are from cryotomograms denoised by CryoCARE.

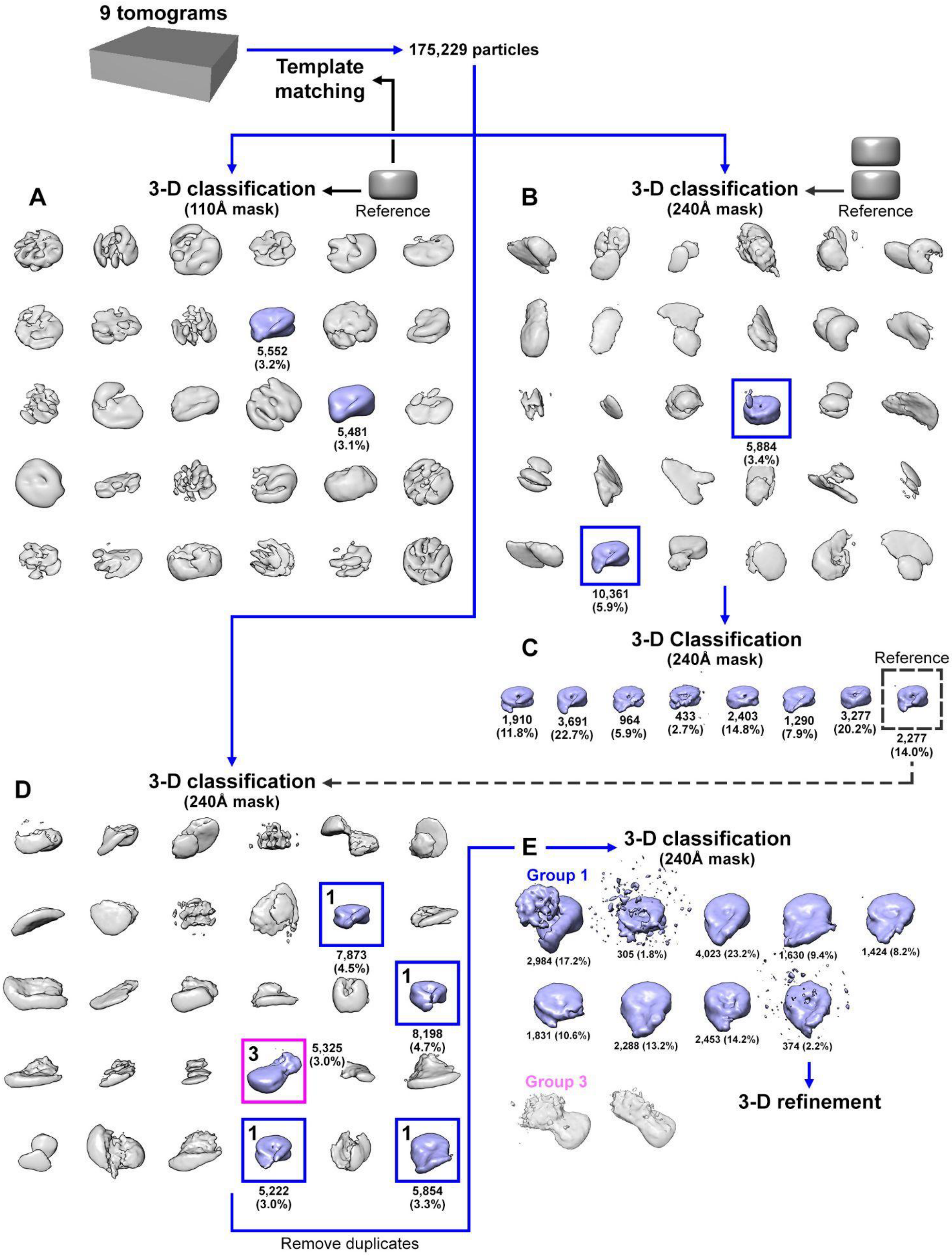
9 tomograms
175,229 particles
Template matching
A
3-D classification
(110Å mask)
Reference
5,552
(3.2%)
5,481
(3.1%)
B
3-D classification
(240Å mask)
Reference
5,884
(3.4%)
10,361
(5.9%)
C
3-D Classification
(240Å mask)
Reference
1,910
(11.8%)
3,691
(22.7%)
964
(5.9%)
433
(2.7%)
2,403
(14.8%)
1,290
(7.9%)
3,277
(20.2%)
2,277
(14.0%)
D
3-D classification
(240Å mask)
1
7,873
(4.5%)
1
8,198
(4.7%)
3
5,325
(3.0%)
1
5,222
(3.0%)
1
5,854
(3.3%)
Remove duplicates
E
3-D classification
(240Å mask)
Group 1
2,984 (17.2%)
305 (1.8%)
4,023 (23.2%)
1,630 (9.4%)
1,424 (8.2%)
1,831 (10.6%)
2,288 (13.2%)
2,453 (14.2%)
374 (2.2%)
Group 3
3-D refinement

Appendix Figure S18. Classification flowchart of G1 chromatin domains for cells without cryoprotection.

The same classification workflow used in the 9% DMSO- (Appendix Fig S12) and 9% glycerol-cryoprotected (Appendix Fig S15) G1 datasets was used for the analysis here. Tomograms collected from G1 cells plunge frozen without cryoprotection were subjected to template matching using a featureless cylindrical reference. (A) Subsequently, the candidate nucleosomes from template matching were classified in 3-D using the cylindrical reference and a 110 Å spherical mask. (B) In parallel, the candidate nucleosomes were also classified using a stacked cylinder reference and a larger 240 Å spherical mask. (C) Nucleosome class averages detected in panel B were then subjected to a second round of 3-D classification, using one of the nucleosome class averages from Panel B as the reference. The stacked dinucleosome class average was not detected at this stage. (D) The original set of candidate nucleosomes was once again subjected to 3-D classification, this time using one of the nucleosome class averages from panel C (dashed box) as the reference. Several mononucleosome class averages (Group 1, boxed blue) and one class average resembling the mononucleosome with a proximal-gyre density (Group 3, boxed magenta) were detected here. (E) Nucleosomes in Group 1 and 3 were then separately subjected to another round of classification. The Group 1 mononucleosomes obtained in this round of classification were then subjected to refinement (Appendix Fig S19). For Group 3, the resultant class averages did not reveal a clear nucleosome density and thus were not analyzed further. Nucleosome class averages are colored blue; ambiguous class averages are colored gray.

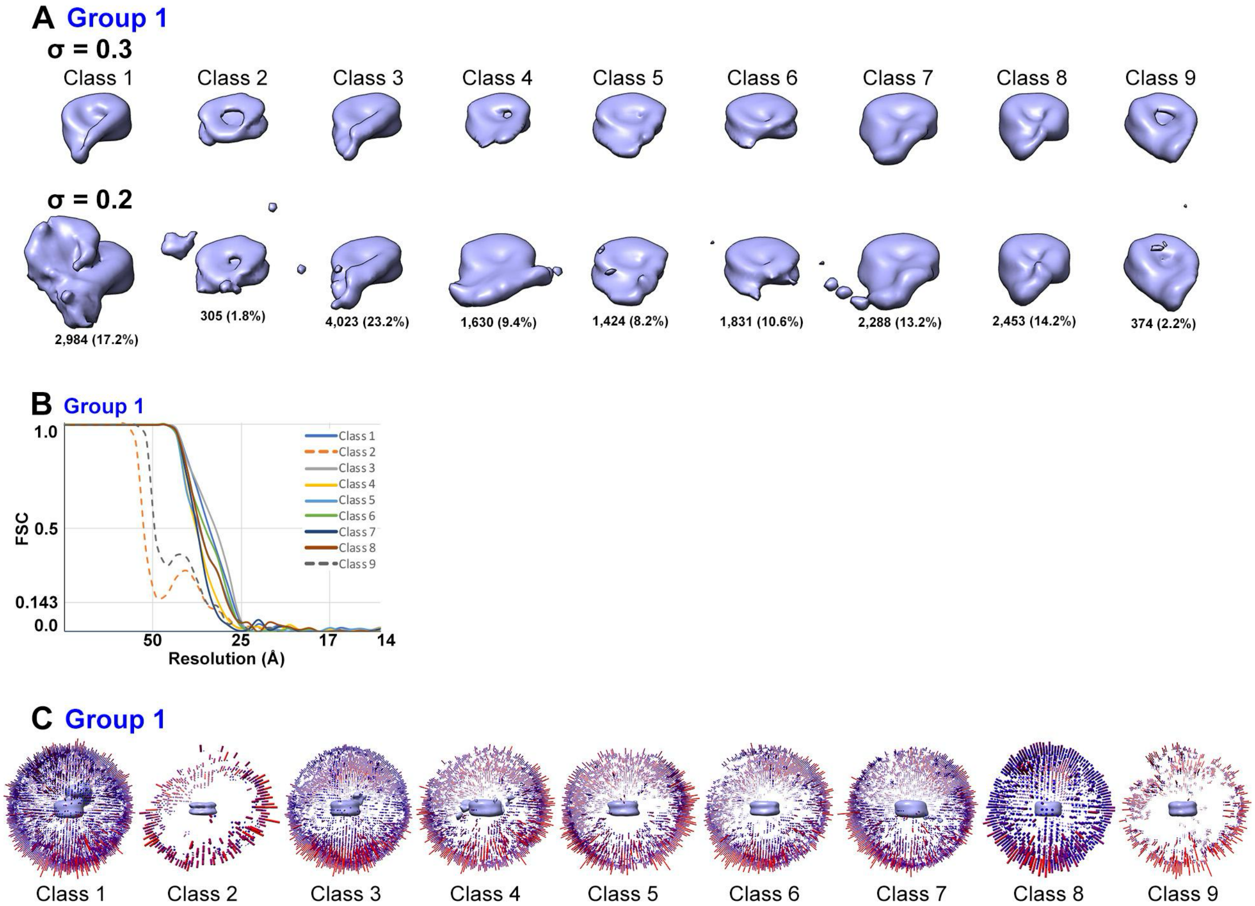

Appendix Figure S19. Refinement of Group 1 nucleosome class averages from G1 cells without cryoprotection.
(A) Refined mononucleosome class averages, rendered at two different threshold levels. The FSC plot and angular distributions of the class averages are shown in panels B and C, respectively.

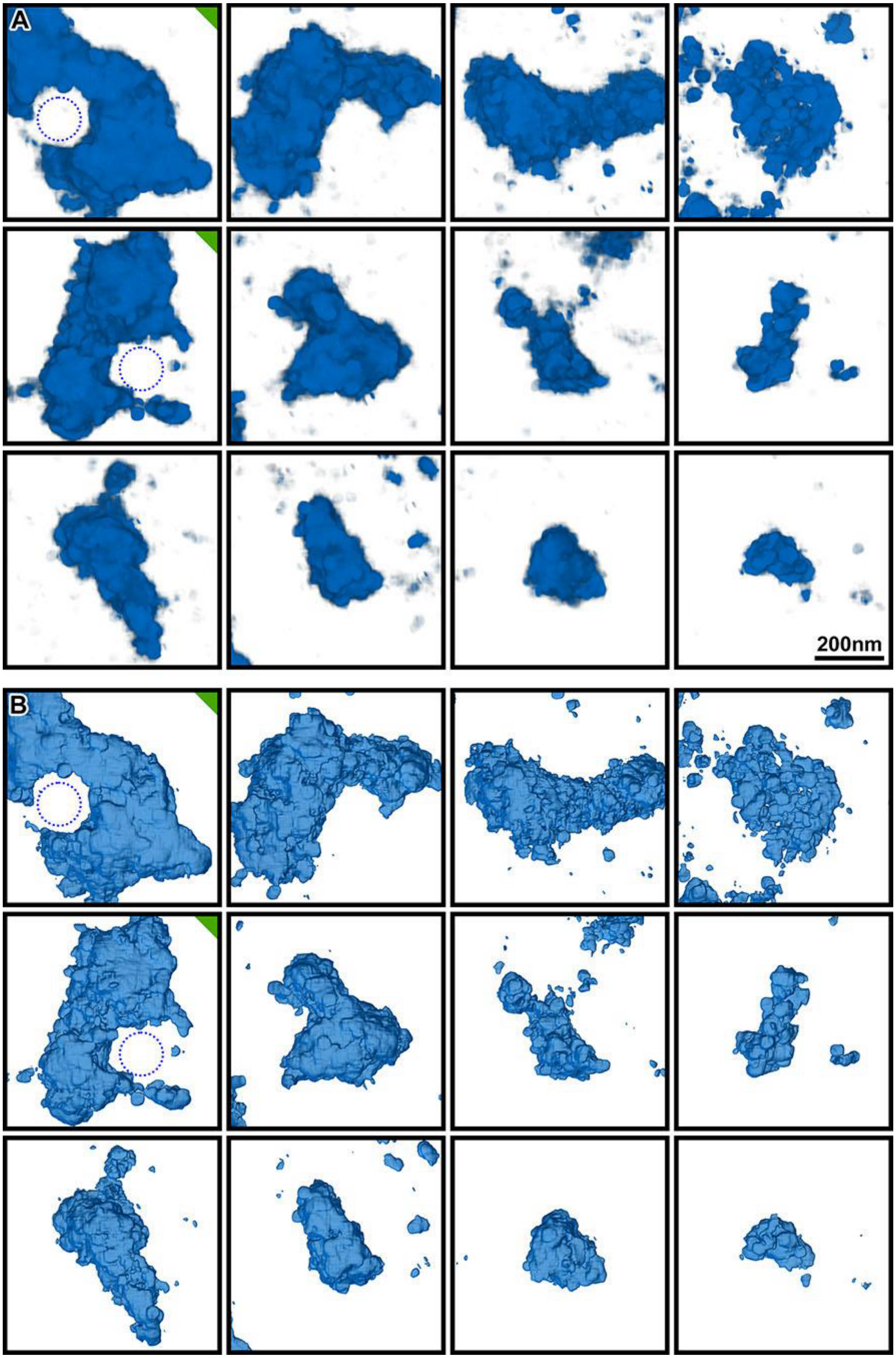
A
200nm
B

Appendix Figure S20. Additional CNN annotations of chromatin domains in G1 nuclei.
(A) Volume and (B) isosurface renderings of chromatin domains. Panels marked with a green triangle contain perinuclear chromatin domains. The dotted blue circles indicate the approximate positions of nuclear pore complexes (not segmented).

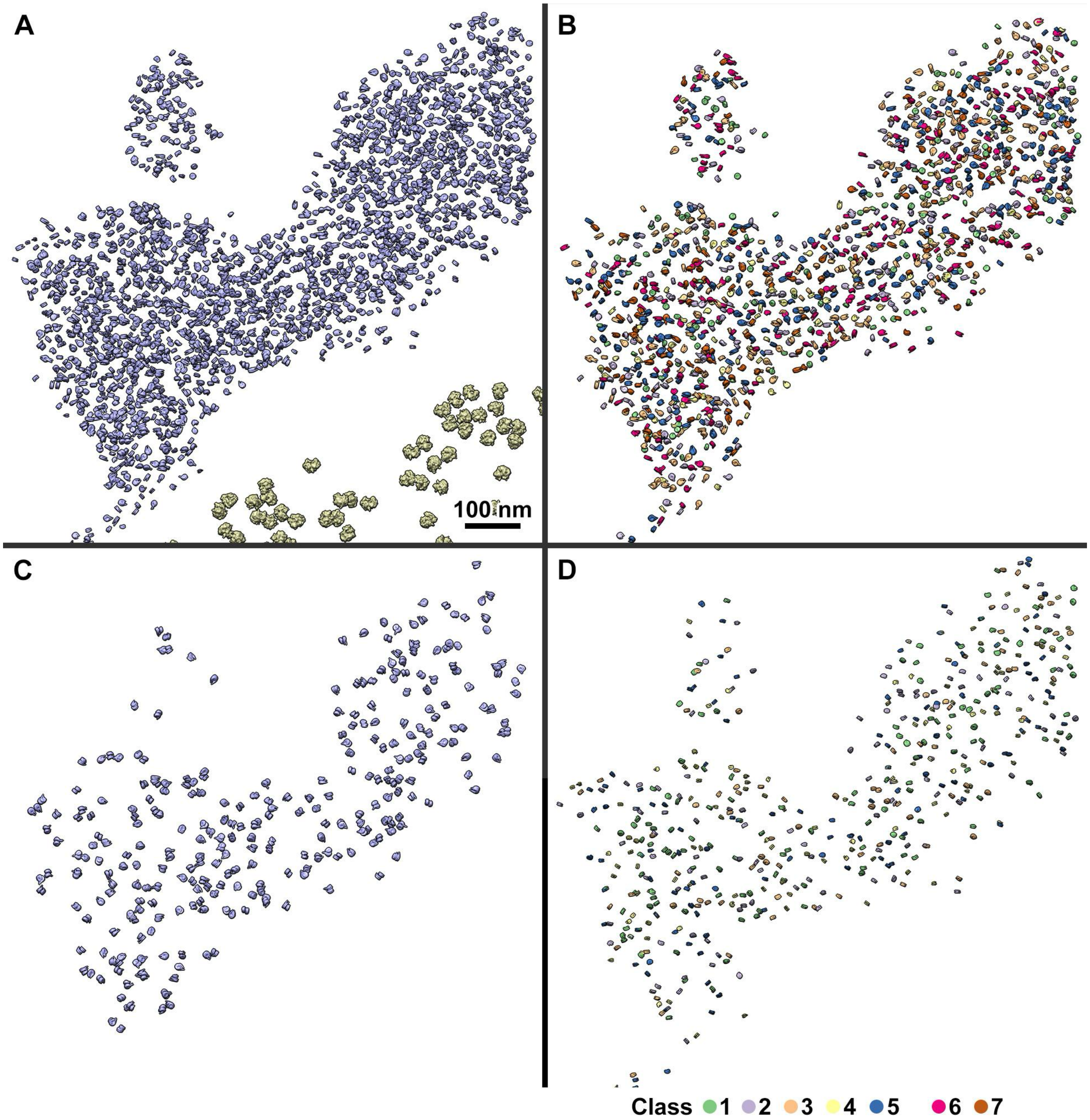

Appendix Figure S21. Remapped models of G1 nucleosome groups.
(A) Enlargement of a segment of Fig 3D showing all remapped nucleosomes (blue) in a G1 domain. Class averages of mononucleosomes (group 1), ordered stacked dinucleosomes (group 2) and mononucleosomes with gyre-proximal density (group 3) are remapped separately in panels B, C and D respectively. The color-coding of the nucleosome densities in panels B and D correspond to the class averages shown in Fig 3A and Fig 3C.

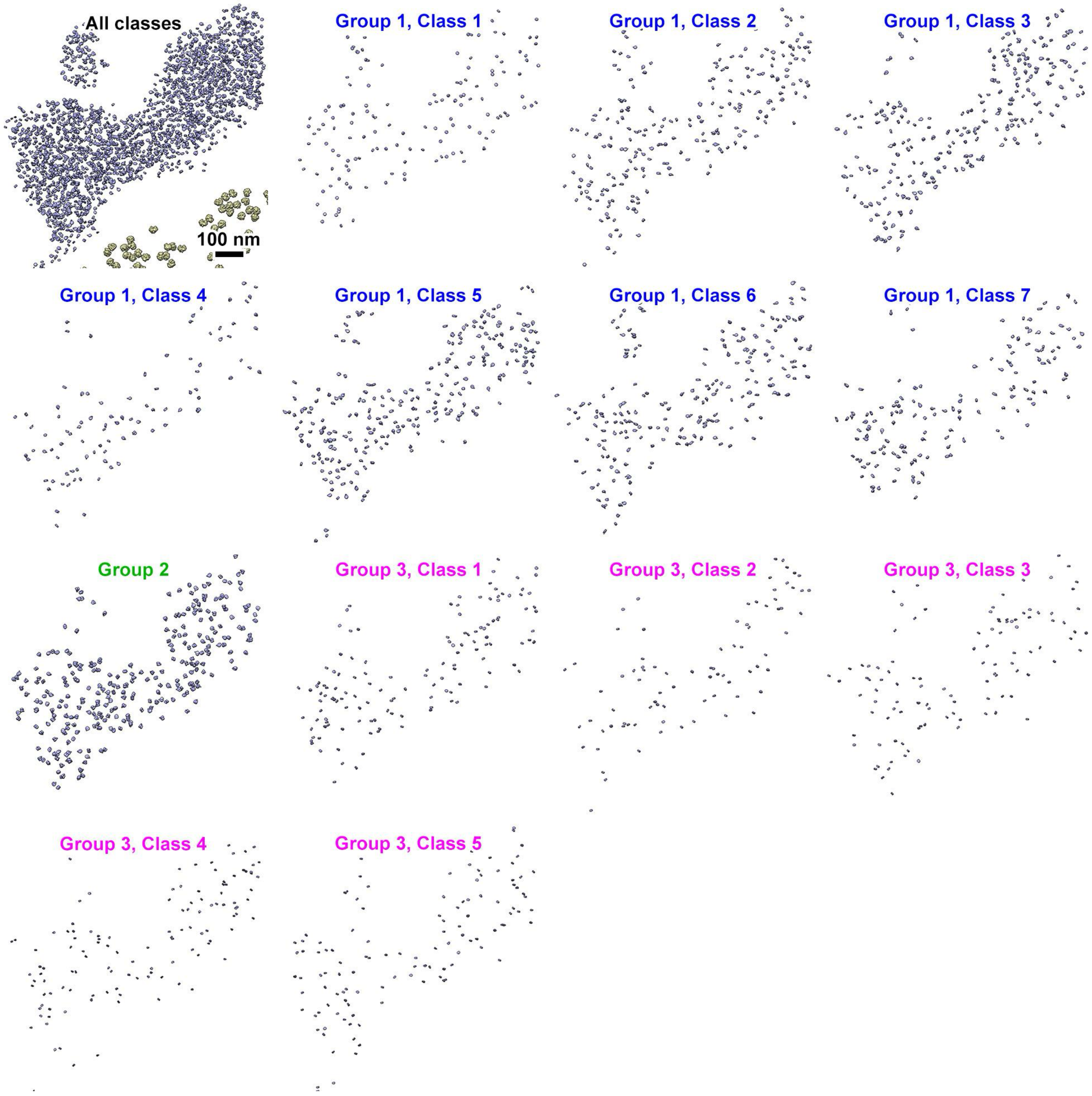

Appendix Figure S22. Remapped models of G1 nucleosome individual classes.
The upper left panel shows a section of the remapped model presented in Fig 3D. The remaining panels also show the same section of the remapped model, but with each of the nucleosome class averages from Fig 3A – C rendered separately.

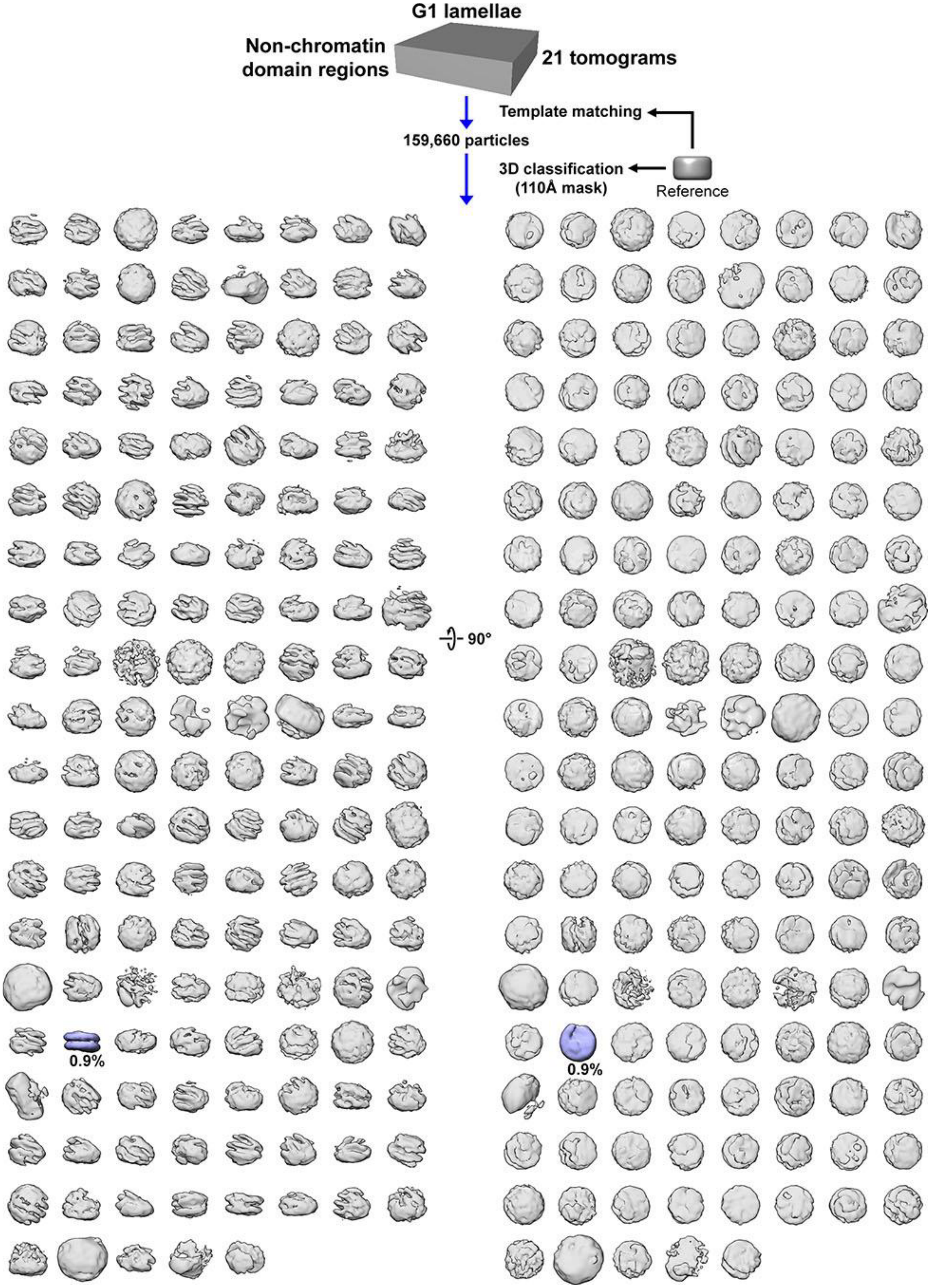
G1 lamellae
Non-chromatin domain regions
21 tomograms
Template matching
159,660 particles
3D classification (110Å mask)
Reference
90°
0.9%
0.9%

Appendix Figure S23. Subtomogram analysis of the G1 nucleoplasm.
The regions outside the chromatin domains were template matched with a featureless cylinder reference. The template-matching hits were then subjected to direct 3-D classification with 200 classes using the same featureless cylinder as the reference. Out of the 157 non-empty classes, the vast majority do not resemble canonical nucleosomes (gray). Only one canonical nucleosome class average was detected (blue), but the subtomograms from this class were mostly located at the periphery of chromatin domains.

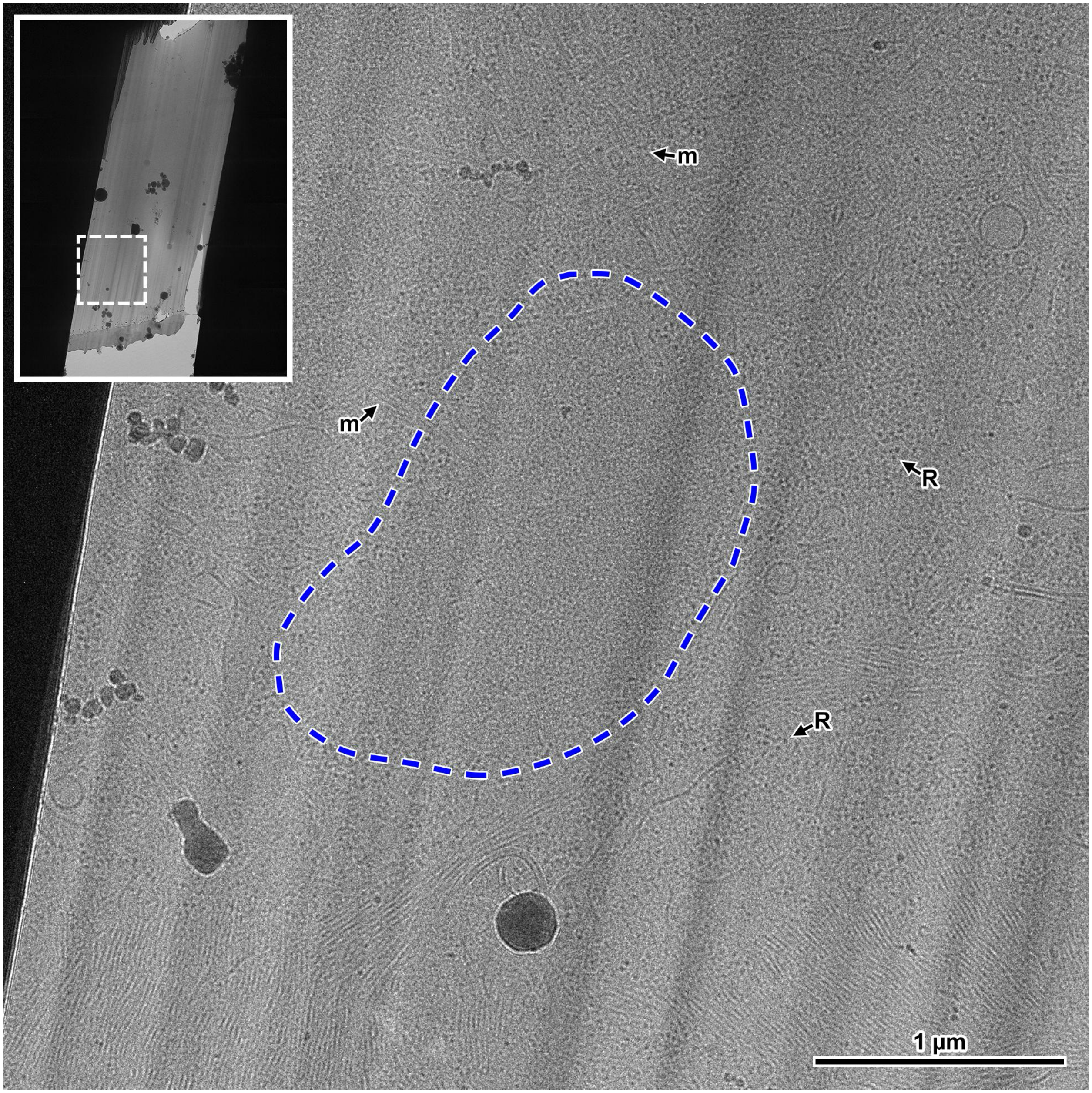

Appendix Figure S24. Targeting images for chromatin in metaphase cells.
Inset shows a montage of an entire lamella – the area bounded by the white dashed line corresponds to the location of the enlarged view. The region indicated by the blue dashed line contains chromatin and is shown at higher magnification as a cryotomographic slice in Fig 4C. Microtubules (m) and ribosomes (R) are also observed, and guided tilt-series target selection.

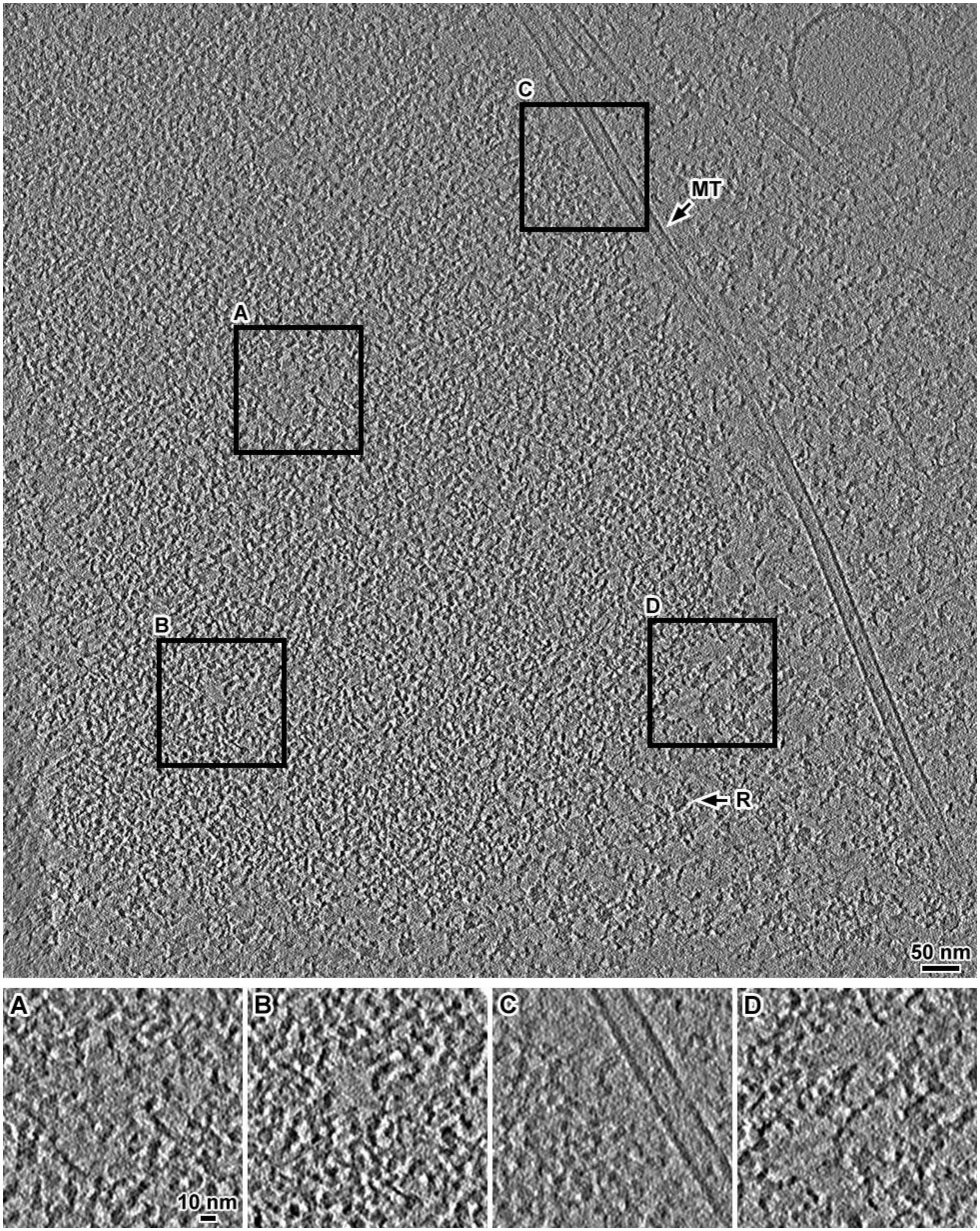

Appendix Figure S25. Additional example Volta cryotomographic slice of a metaphase cell.
Cryotomographic slice (20 nm) centered on a mitotic chromatin in a metaphase RPE-1 cell. Rendered with low JPEG compression. Insets show 2-fold enlargements of nucleosome-free

pockets inside the chromosome (A and B) and the chromosome periphery (C and D). Cytological features are highlighted: ribosome (R); microtubule (MT).

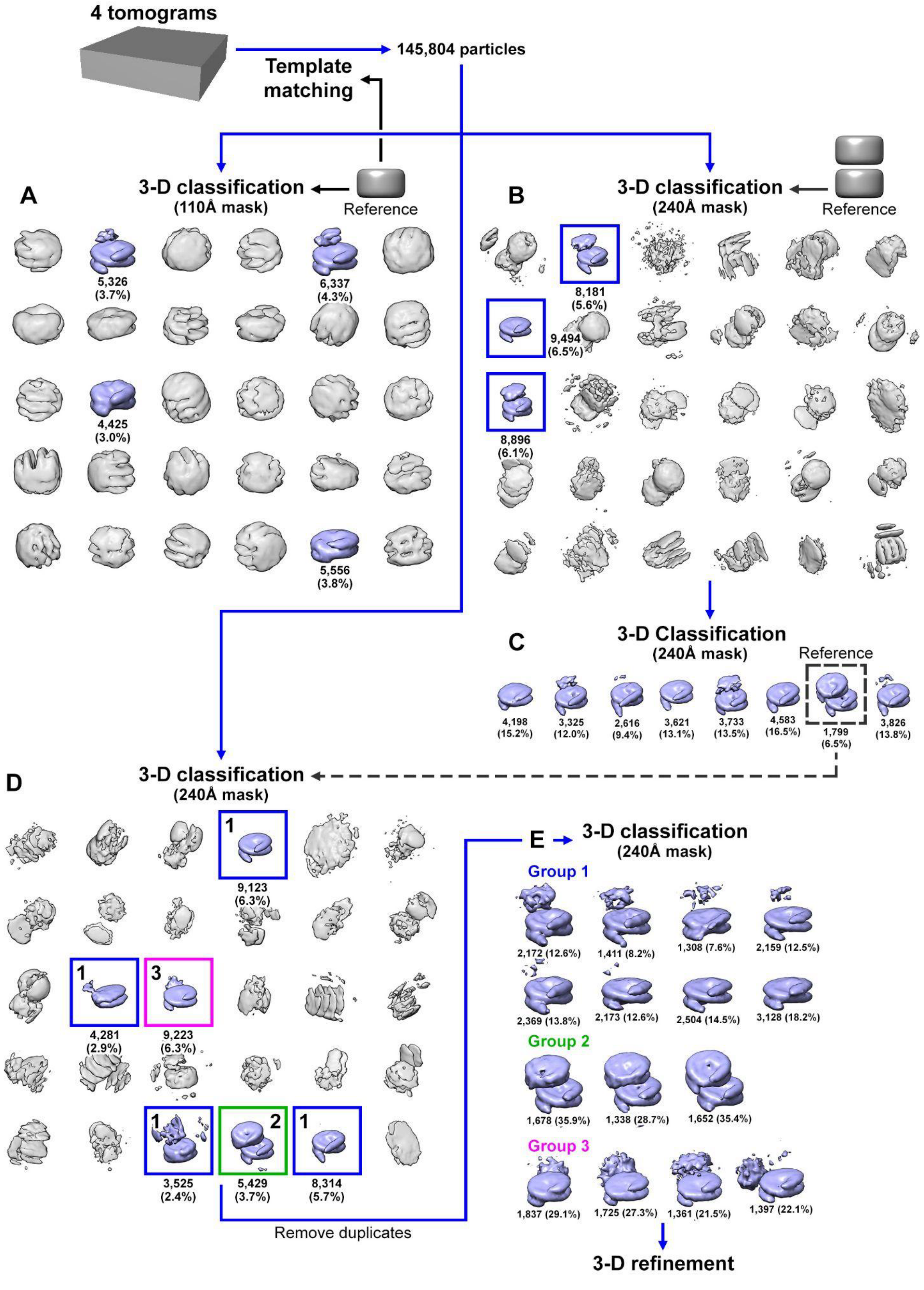

4 tomograms
145,804 particles
Template matching
A
3-D classification
(110Å mask)
Reference
5,326
(3.7%)
6,337
(4.3%)
4,425
(3.0%)
5,556
(3.8%)
B
3-D classification
(240Å mask)
Reference
8,181
(5.6%)
9,494
(6.5%)
8,896
(6.1%)
C
3-D Classification
(240Å mask)
Reference
4,198
(15.2%)
3,325
(12.0%)
2,616
(9.4%)
3,621
(13.1%)
3,733
(13.5%)
4,583
(16.5%)
1,799
(6.5%)
3,826
(13.8%)
D
3-D classification
(240Å mask)
9,123
(6.3%)
4,281
(2.9%)
9,223
(6.3%)
3,525
(2.4%)
5,429
(3.7%)
8,314
(5.7%)
Remove duplicates
E
3-D classification
(240Å mask)
Group 1
2,172 (12.6%)
1,411 (8.2%)
1,308 (7.6%)
2,159 (12.5%)
2,369 (13.8%)
2,173 (12.6%)
2,504 (14.5%)
3,128 (18.2%)
Group 2
1,678 (35.9%)
1,338 (28.7%)
1,652 (35.4%)
Group 3
1,837 (29.1%)
1,725 (27.3%)
1,361 (21.5%)
1,397 (22.1%)
3-D refinement

Appendix Figure S26. Classification flowchart of M cells.
(A) Metaphase candidate nucleosome subtomograms that were template matched with a cylindrical reference were directly classified in 3-D, using a cylindrical reference and a 120 Å spherical mask. (B) In parallel, the same set of subtomograms were directly classified in 3-D using a stacked cylinder reference and a larger spherical mask. The ambiguous densities (gray) are not canonical nucleosomes; they are abundant because the template matching process uses a featureless cylinder reference and a low cross-correlation cutoff. As a result, large numbers of false positives are rejected in the classification analysis. (C) A second round of classification using a nucleosome class average from B as the reference and the larger mask yielded mononucleosomes with extra densities at their face plus an unambiguous dinucleosome class. (D) Direct 3-D classification was done on the original set of subtomograms using a larger mask and the stacked dinucleosome class average from panel C as the reference. Three groups of class averages were obtained, corresponding to (1) mononucleosome, (2) stacked dinucleosome, and (3) mononucleosome with a gyre-proximal density. (E) These three groups of class averages were subjected to a third round of classification using either a mononucleosome, stacked dinucleosome or mononucleosome with a gyre-proximal density from panel D as the reference and large spherical mask.

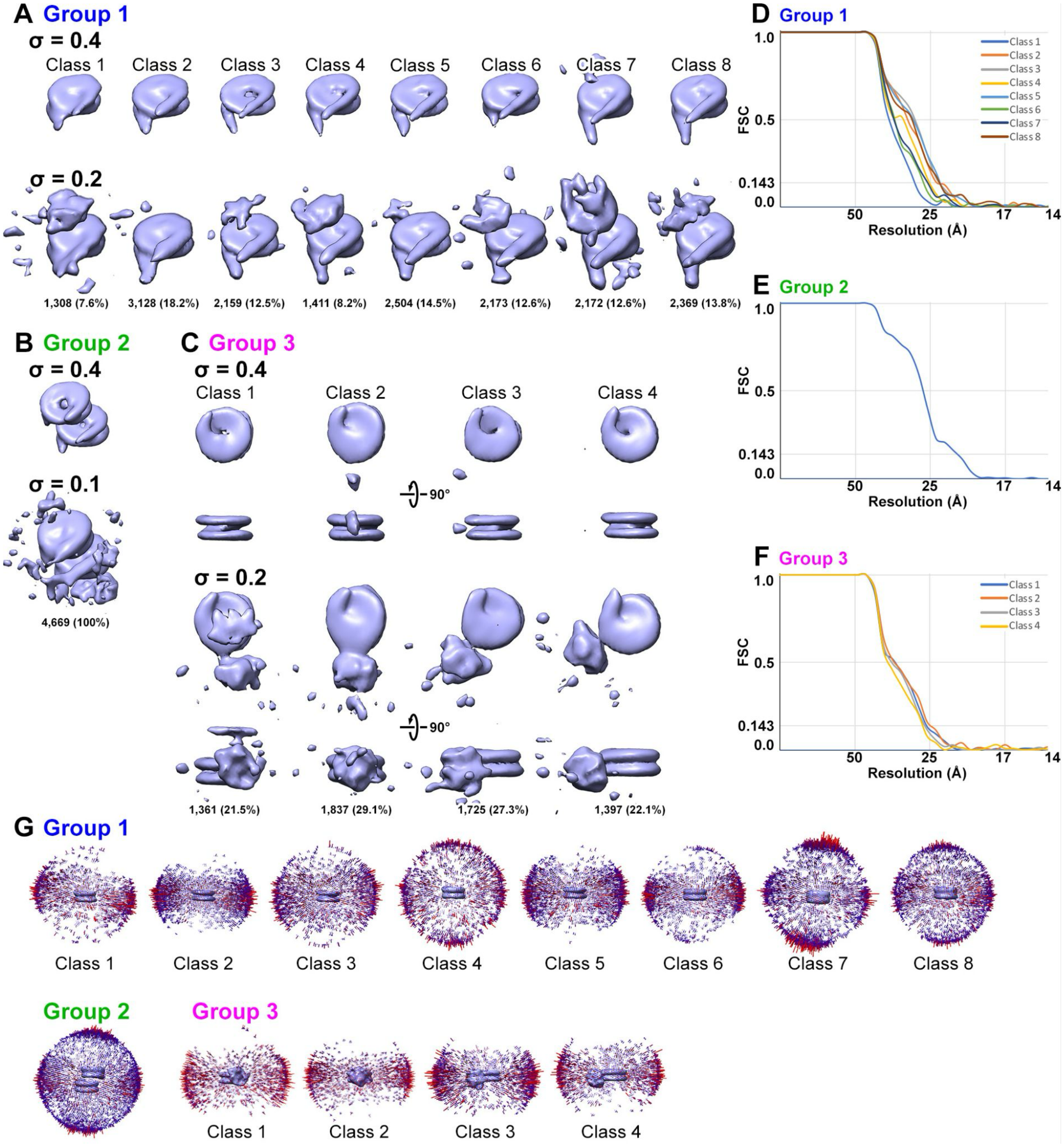

Appendix Figure S27. Refinement of metaphase mononucleosomes and dinucleosomes. Refined class averages for (A) mononucleosomes, (B) stacked dinucleosomes, and (C) mononucleosomes with a gyre-proximal density. Two contour levels are shown for each class average. (D, E, F) FSC plots and (G) angular distribution of the refined density maps. For panels A – C, the hide-dust feature in UCSF Chimera was disabled.

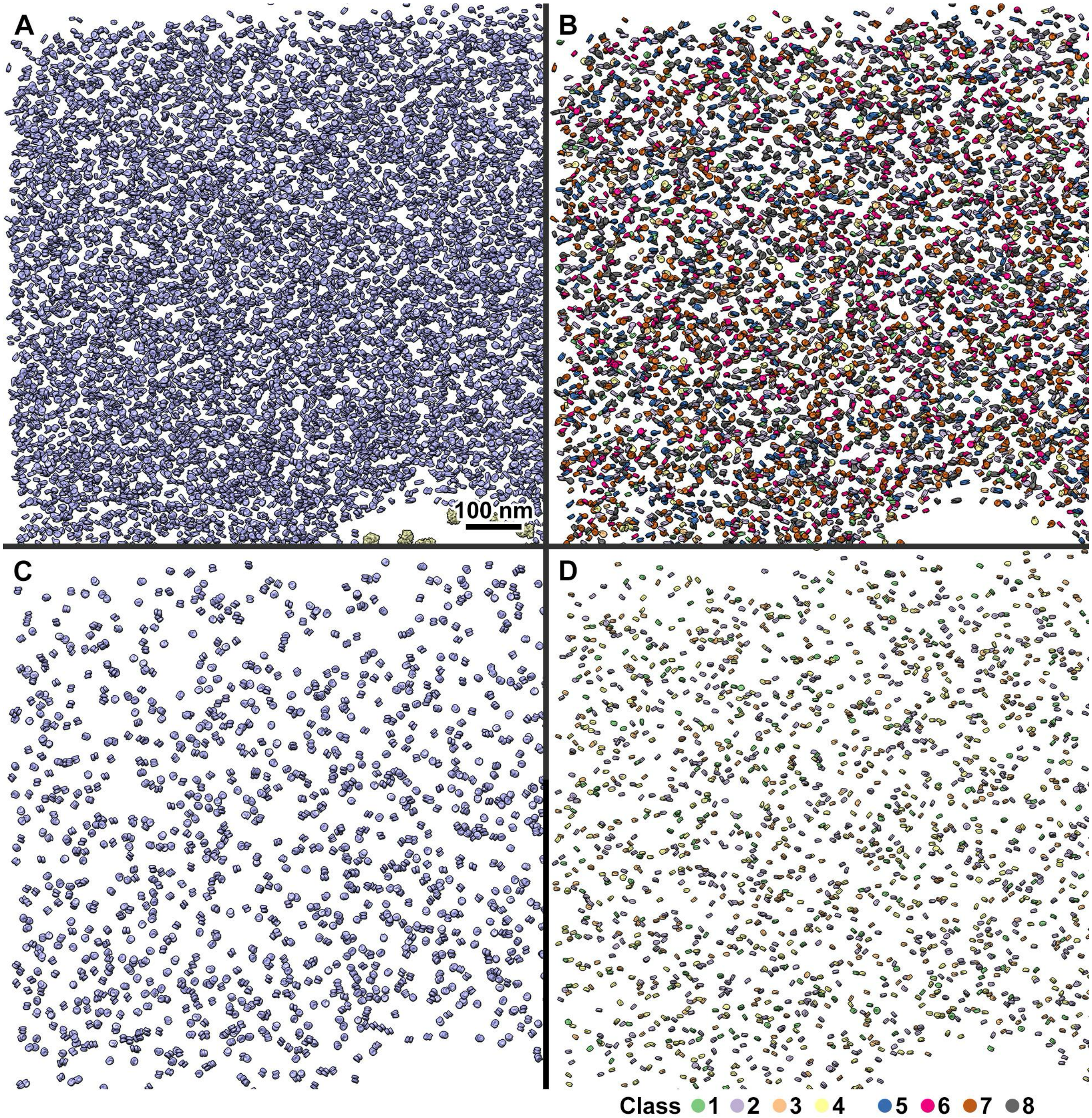

Appendix Figure S28. Remapped models of metaphase nucleosome groups.
(A) Enlargement of a segment of Fig 5D showing all remapped nucleosomes (blue) in a G1 domain. Class averages of mononucleosomes (group 1), ordered stacked dinucleosomes (group 2) and mononucleosomes with gyre-proximal density (group 3) are remapped separately in panels B, C and D respectively. The color-coding of the nucleosome densities in panels B and D correspond to the class averages shown in Fig 5A and Fig 5C.

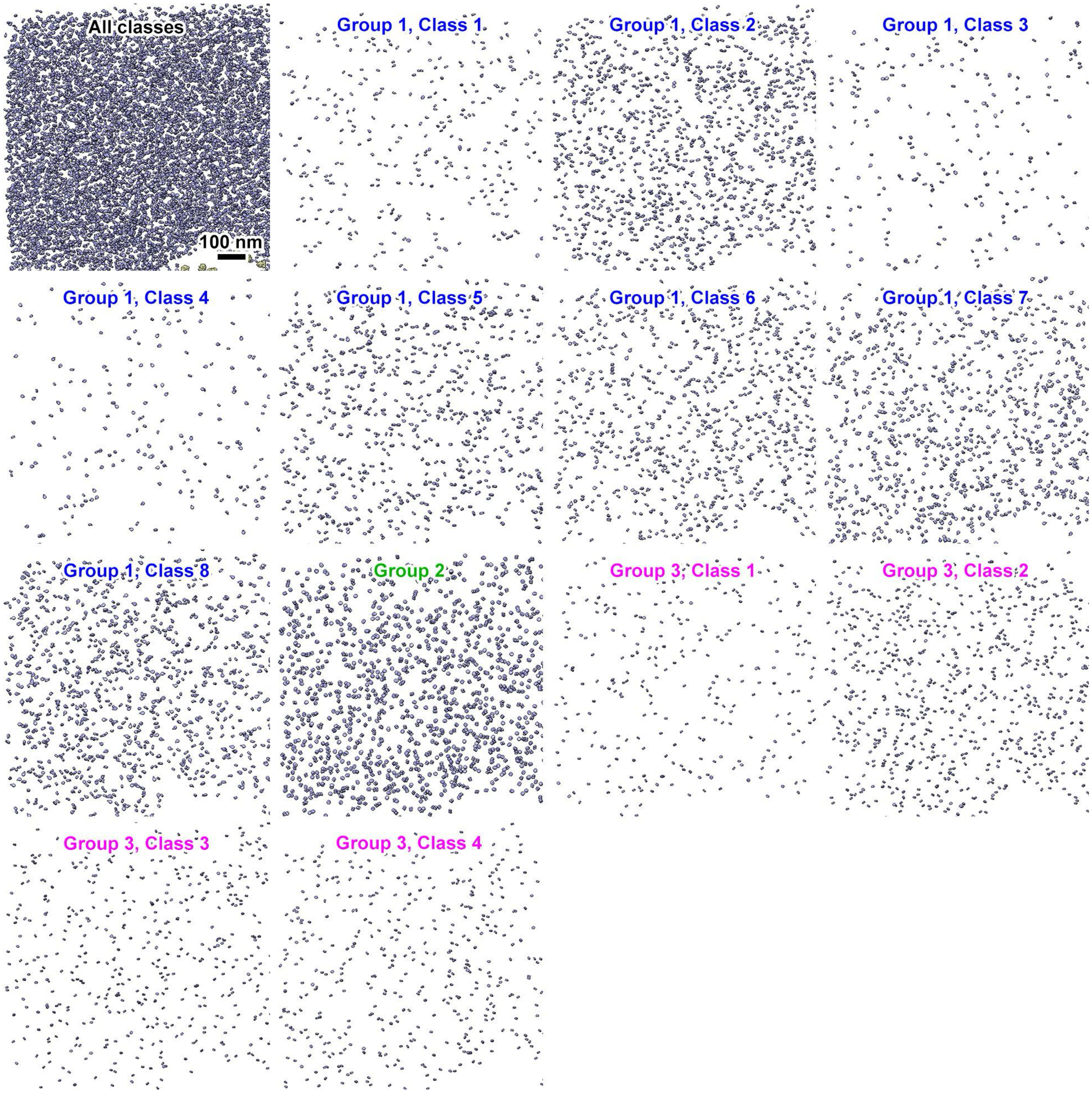

Appendix Figure S29. Remapped models of metaphase nucleosome individual classes. The upper left panel shows a section of the remapped model presented in Fig 5D. The remaining panels also show the same section of the remapped model, but with each of the nucleosome class averages from Fig 5A – C rendered separately.

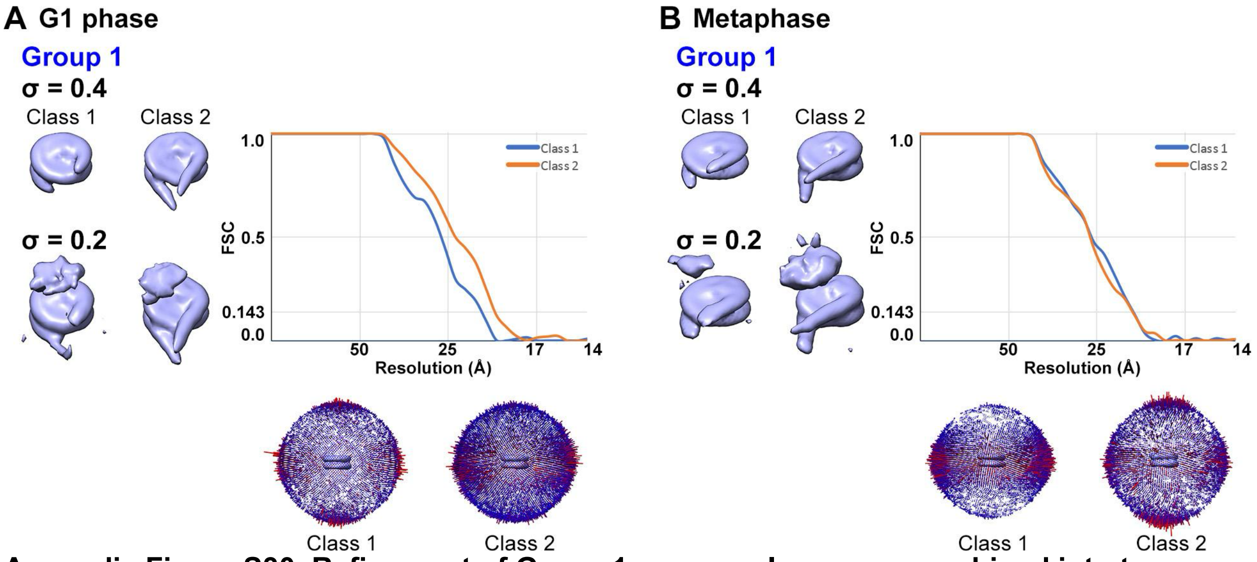

Appendix Figure S30. Refinement of Group 1 mononucleosomes combined into two classes.

Mononucleosome classes from Group 1 (Figs 3A and 5A) were manually assigned into two groups, based on the length of the linker DNA. Shown here are the refinement results for the short linker (Class 1) and long linker (Class 2) class for (A) G1 and (B) metaphase cells, respectively. The refined mononucleosome class averages here were used for docking, as shown in Fig 7.

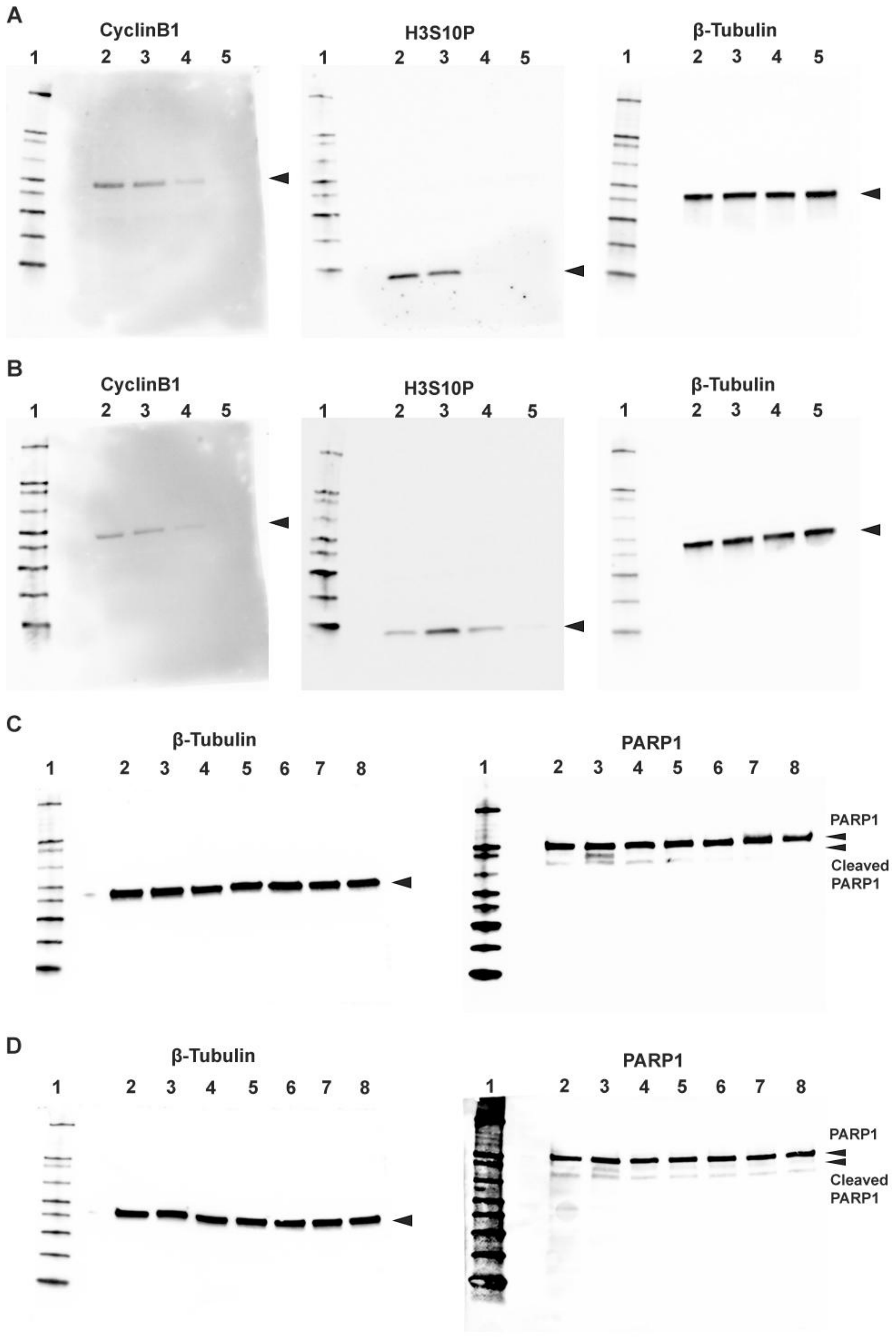
A
CyclinB1
1 2 3 4 5
H3S10P
1 2 3 4 5
β-Tubulin
1 2 3 4 5
B
CyclinB1
1 2 3 4 5
H3S10P
1 2 3 4 5
β-Tubulin
1 2 3 4 5
C
β-Tubulin
1 2 3 4 5 6 7 8
PARP1
1 2 3 4 5 6 7 8
PARP1
Cleaved
PARP1
D
β-Tubulin
1 2 3 4 5 6 7 8
PARP1
1 2 3 4 5 6 7 8
PARP1
Cleaved
PARP1

Appendix Figure S31. Uncropped immunoblots.
(A and B) Uncropped immunoblots from Appendix Fig S1. (C and D) Uncropped immunoblots from Appendix Fig S8. (A) Nocodazole arrest and washout. (B) MG132 arrest and washout. The lane number is indicated above the gels and correspond, respectively, to the protein ladder (Invitrogen, #LC5602); arrested cells; 30 minutes after washout; 60 minutes after washout; 120 minutes after washout. (C) Apoptosis detection in G1 phase RPE1 cells treated with or without cryoprotectant. (D) Apoptosis detection in metaphase RPE1 cells treated with or without cryoprotectants. Lanes 1, 2, 3 in both (C) and (D) correspond respectively to the protein ladder, 0.1% DMSO treatment for 6 hours, and 1 µM staurosporine treatment for 6 hours. Lanes 4, 5, 6, 7, 8 in (C) are G1 phase RPE1 cells, the lanes correspond respectively to the G1 cells, G1 cells treated with 9% DMSO for 1 minute, G1 cells treated with 9% DMSO for 10 minutes, G1 cells treated with 9% glycerol for 1 minute, G1 cells treated with 9% glycerol for 10 minutes. Lanes 4, 5, 6, 7, 8 in (D) are metaphase RPE1 cells, the lanes correspond respectively to the metaphase cells, metaphase cells treated with 9% DMSO for 1 minute, metaphase cells treated with 9% DMSO for 10 minutes, metaphase cells treated with 9% glycerol for 1 minute, metaphase cells treated with 9% glycerol for 10 minutes. The positions of PARP1 and cleaved PARP1 proteins are indicated with the arrowheads.

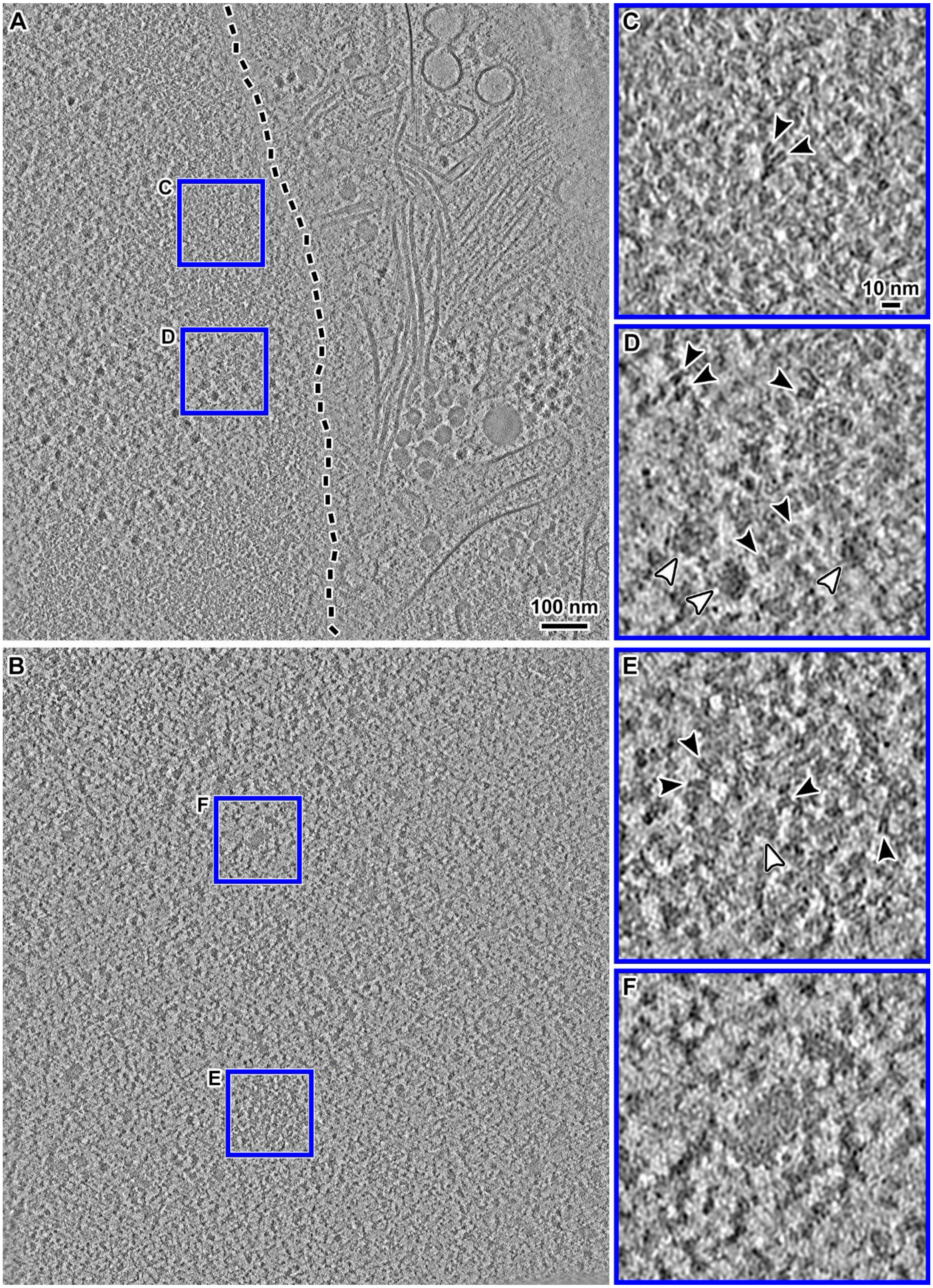

Appendix Figure S32. Non-denoised version of Fig 2.

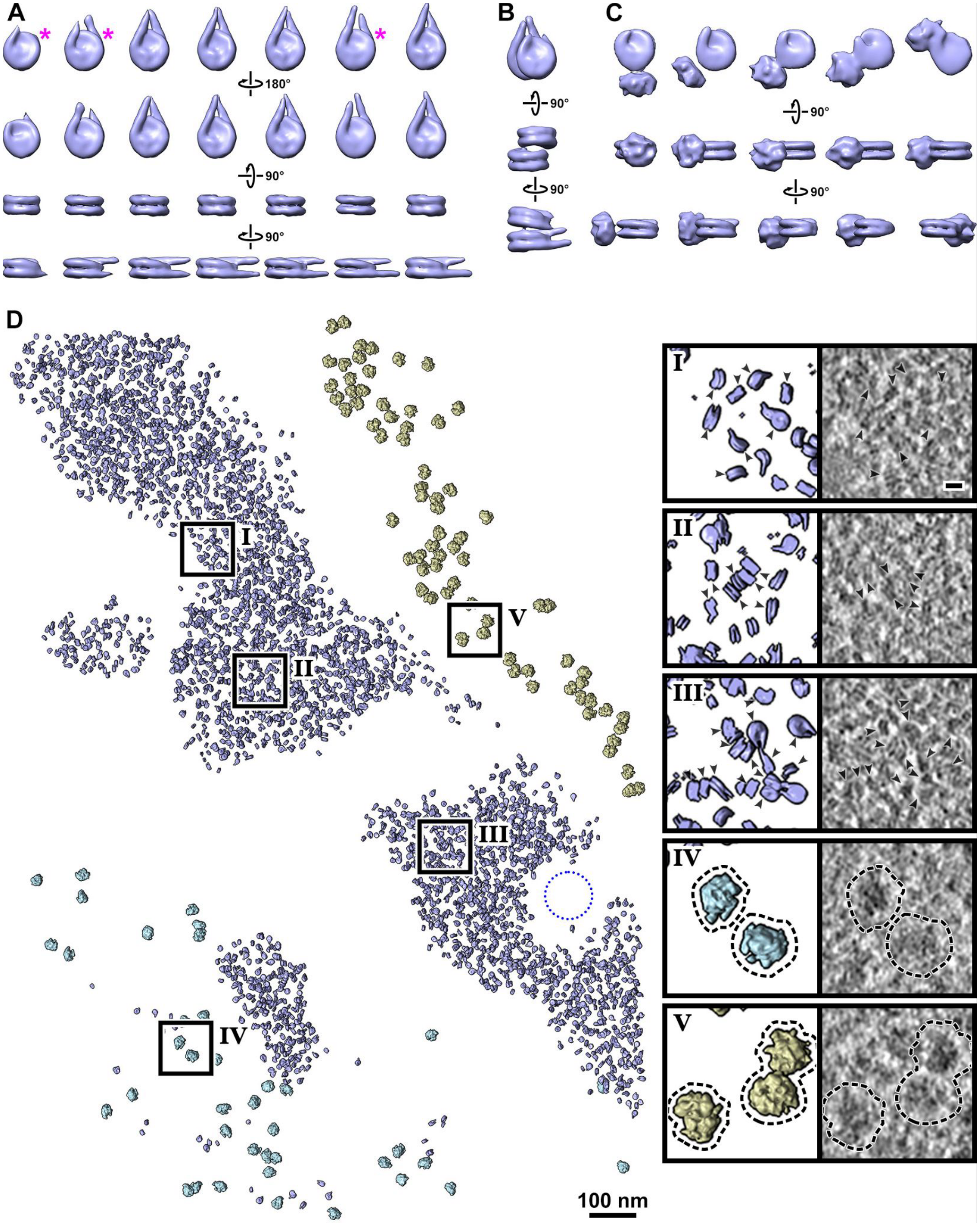

Appendix Figure S33. Reproduction of Fig 3, but with non-denoised versions of the cryotomographic slices.

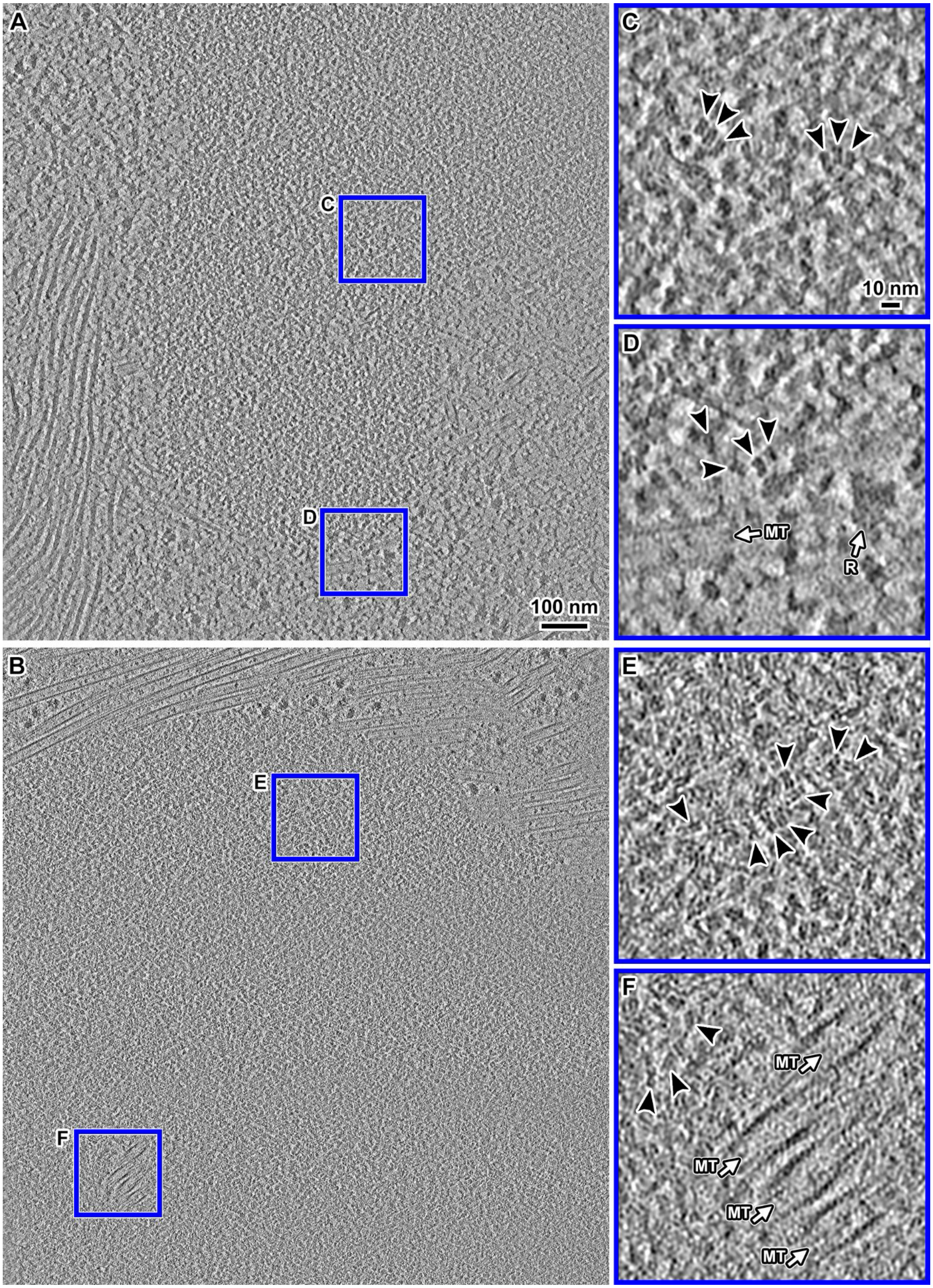

Appendix Figure S34. Non-denoised version of Fig 4.

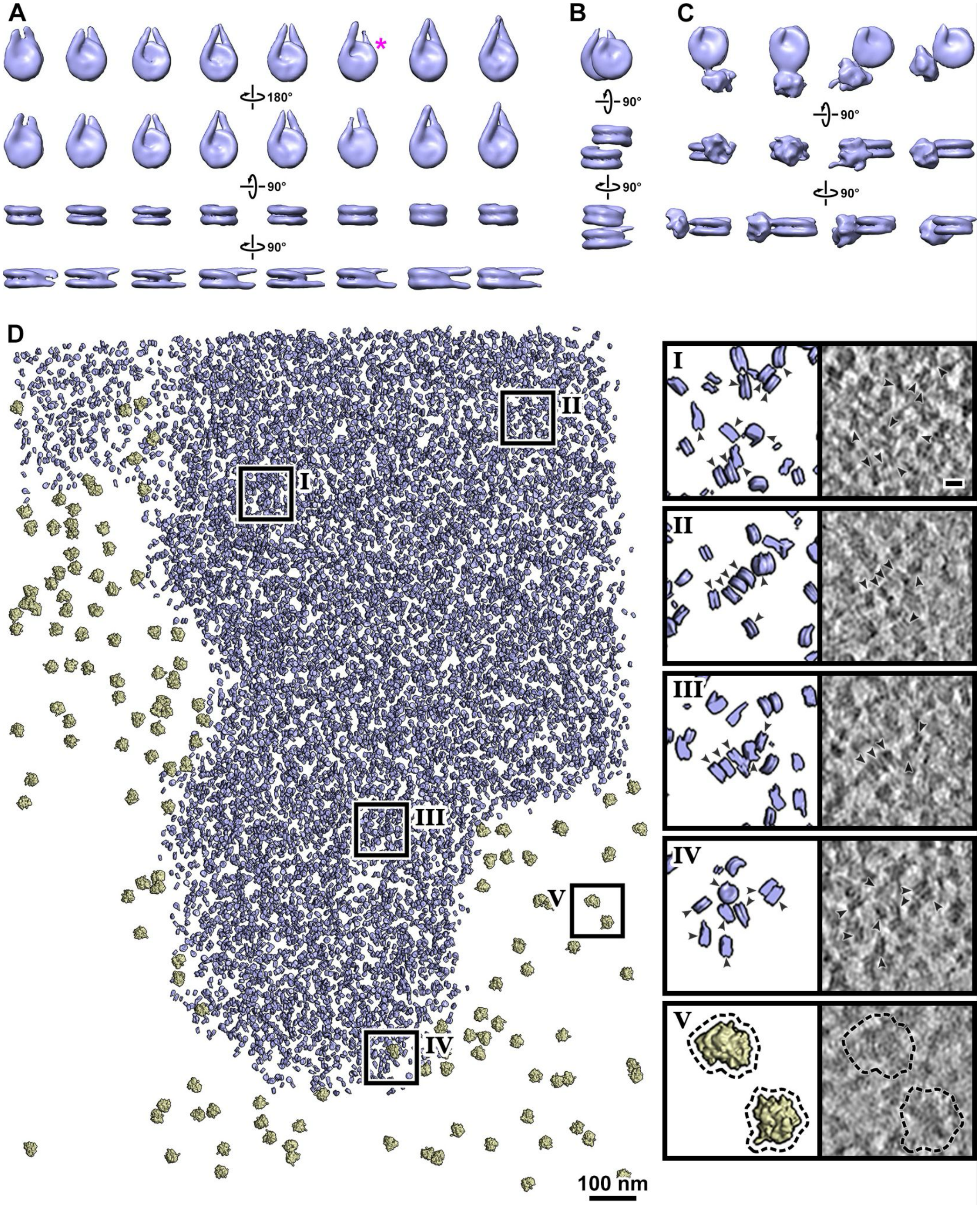

Appendix Figure S35. Reproduction of Fig 5, but with non-denoised versions of the cryotomographic slices.

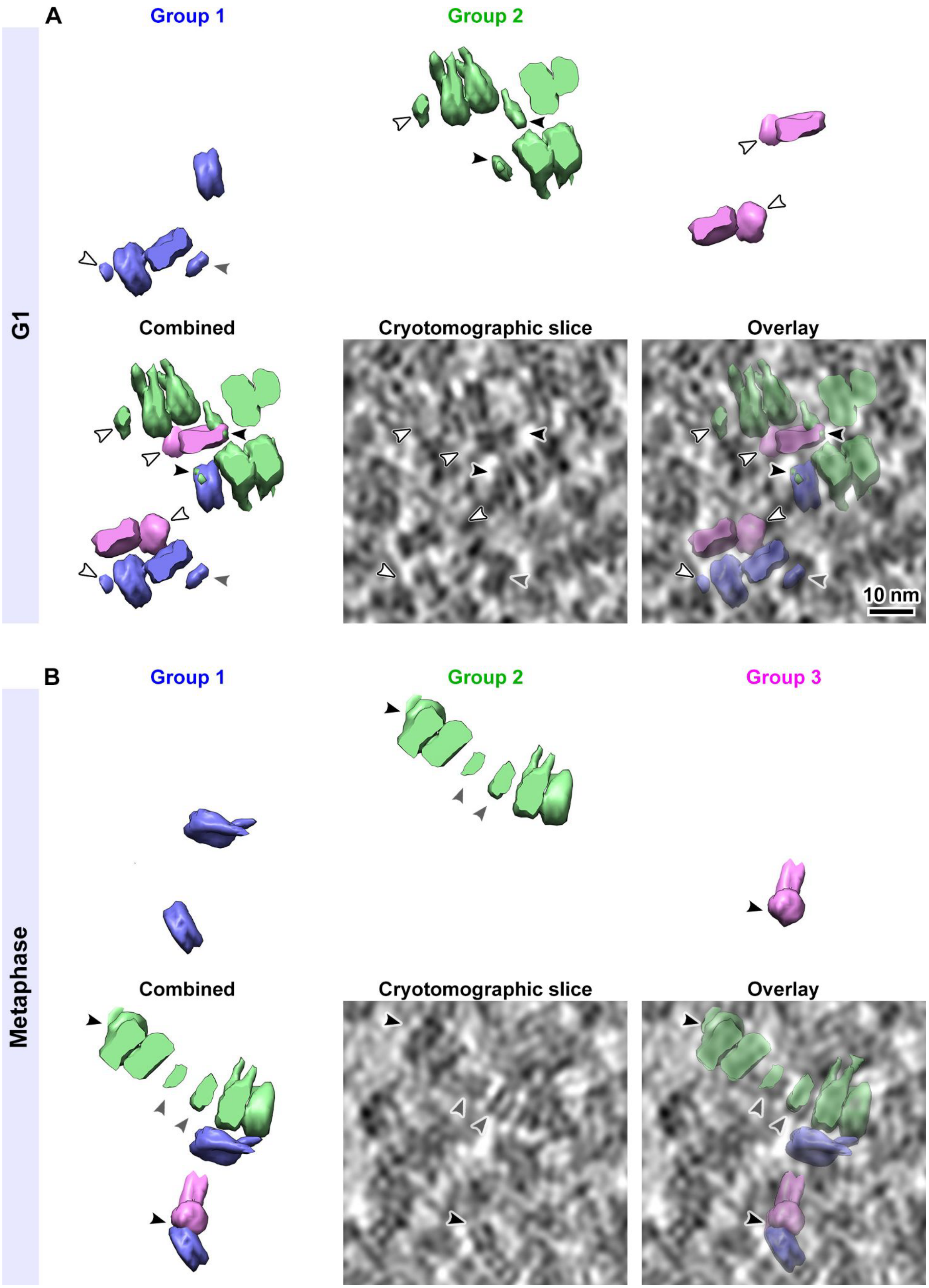

Appendix Figure S36. Reproduction of Fig 6, but with non-denoised versions of the cryotomographic slices.

Appendix Table S1. Research resources.

Resource	Source	Catalog ID / link
**Chemicals**		
4′,6-diamidino-2-phenylindole (DAPI)	TFS	D1306
Dimethyl sulfoxide (DMSO)	Sigma	472301
DMEM/F-12 with GlutaMAX	Gibco/TFS	10565-018
Fetal bovine serum	Sigma	F9665
Glycine	Bio-Rad	1610718
MG132	Merck	M7449-1ML
Nocodazole	Merck	SML1665-1ML
Palbociclib	Selleckchem	S1116
Paraformaldehyde	EMS	15714
Penicillin-streptomycin	Gibco	15140-122
Phosphate buffered saline (PBS)	Vivantis	PB0344-1L
PBS-T (PBS + 0.1% Tween 20)	Sinopharm	T20087687
Prolong Gold antifade reagent	TFS	P36930
Taxol	Santa Cruz	33069-62-4
Triton X-100	Alfa Aesar	A16046
**Antibodies**		
Alexa Fluor® 647 anti-histone H3 (phospho S10)	Abcam	ab196698
Rabbit polyclonal Anti-phospho-Histone H3 (Ser10)	Abcam	ab5176
Mouse anti-rabbit IgG-HRP	Santa Cruz	sc-2357
Rabbit polyclonal to beta Tubulin	Abcam	ab6046
m-IgGκ BP-HRP	Santa Cruz	sc-516102
cyclin B1 (GNS1) mouse monoclonal IgG1	Santa Cruz	sc-245
Anti-RPB1 CTD(S5P)	Abcam	ab5131
Anti-RPB1 CTD(S2P)	Abcam	ab5095
**Cell line**		
hTERT-RPE-1 cells	ATCC	CRL-4000
**Chromatin**		
HeLa oligonucleosomes	EpiCypher	16-0003
**Light microscopy**		
LSM900 AiryScan microscope	Zeiss	N/A
20×, 0.8 N.A. Plan-Apochromat	Zeiss	N/A
63×, 1.4 N.A. oil Plan-Apochromat	Zeiss	N/A
ZEN 3.0 (blue edition)	Zeiss	N/A
Eclipse Ti microscope	Nikon	N/A
Plan Fluor 10x Ph1 objective	Nikon	N/A
20 mm Ø coverslip	Paul Marienfeld GmbH	0112600
**Electron cryomicroscopy**		

C-flat 2/4 200 mesh, gold	Protochips	CF-2/4-2Au
C-Clip Ring (autogrid)	TFS	1036173
CryoFIB autogrid	TFS	1205101
C-flat 2/4 200 mesh, copper	Protochips	CF-2/4-2C
Continuous carbon	EMS	CF200-Cu-UL
Whatman Grade 1 filter paper	Whatman	1001-055
Copper tubes, 0.3mm inner diameter	Wohlwend	N/A
Copper tube loading tool	Wohlwend	Part 733-1
Copper tube cutting device	Wohlwend	Part 732
UC7/FC7 Microtome	Leica	N/A
35° diamond knife	Diatome	Cryo35
Micromanipulator, Leica	Leica	N/A
Micromanipulator, MN-151-S	Narishige	N/A
Vitrobot Mark IV	TFS	N/A
Helios Nanolab 650 FIB-SEM	TFS	N/A
PolarPrep 2000 Cryo Transfer System	Quorum	N/A
Titan Krios G1 cryo-TEM	TFS	N/A
Falcon II camera	TFS	N/A
Gatan K3 Summit camera	AMETEK	N/A
Gatan BioContinuum imaging filter	AMETEK	N/A
**Software**		
Adobe Illustrator	Adobe	adobe.com
Adobe Photoshop	Adobe	adobe.com
Adobe Premiere Pro	Adobe	adobe.com
Auxiliary scripts	Gan lab	github.com/anaphaze/ot-tools
BioRender	BioRender	biorender.com
Bsoft	(Heymann & Belnap, 2007)	lsbr.niams.nih.gov/bsoft
cryo-CARE	(Buchholz *et al*, 2019)	github.com/juglab/cryoCARE_pip
EMAN2	(Chen *et al*, 2017)	blake.bcm.edu/emanwiki/EMAN2
FIJI	(Schindelin *et al*, 2012)	imagej.net/software/fiji
Google sheets	Google	www.google.com/sheets
IMOD	(Mastronarde, 1997)	bio3d.colorado.edu/imod
PEET	(Nicastro *et al*, 2006)	bio3d.colorado.edu/PEET
RELION	(Scheres, 2012)	github.com/3dem/relion
SerialEM	(Mastronarde, 2005)	bio3d.colorado.edu/SerialEM
UCSF Chimera	(Pettersen *et al*, 2004)	www.cgl.ucsf.edu/chimera

Appendix Table S2. Antibodies used.

Antigen	1º antibody or conjugated antibody	2º antibody	Dilution 1º	2º
**Immunoblots**				
H3S10P	Rabbit polyclonal Anti-phospho-Histone H3 (Ser10) Antibody (Abcam ab5176)	mouse anti-rabbit IgG-HRP (Santa Cruz sc-2357)	1:500	1:5000
Cyclin B	cyclin B1 (GNS1) mouse monoclonal IgG1 (Santa Cruz sc-245)	m-IgGκ BP-HRP (Santa Cruz sc-516102)	1:500	1:5000
Beta Tubulin	Rabbit polyclonal to beta Tubulin (Abcam ab6046)	mouse anti-rabbit IgG-HRP (Santa Cruz sc-2357)	1:500	1:5000
PARP1	Rabbit monoclonal [EPR18461] to PARP1	mouse anti-rabbit IgG-HRP (Santa Cruz sc-2357)	1:500	1:5000
**Immunofluorescence**				
H3S10P	Alexa Fluor 647 Mouse Monoclonal Anti-Histone H3 (phospho S10) (Abcam ab196698)	N/A	1:250	N/A
RPB1 CTD(S5P)	Rabbit polyclonal, (Abcam ab5131)	Goat anti-Rabbit IgG (H+L) Secondary Antibody, Alexa Fluor 488 (TFS A-11008)	1:300	1:500
RPB1 CTD(S2P)	Rabbit polyclonal, (Abcam ab5095)	Goat anti-Rabbit IgG (H+L) Secondary Antibody, Alexa Fluor 488 (TFS A-11008)	1:300	1:500

TFS = Thermo Fisher Scientific.

Appendix Table S3. Confocal microscopy details.

**General**	
Model	Zeiss LSM900
Control software	cellSense v X.Y
Pinhole	1 AU
X, Y pixel	0.087 [μm]
Z pixel	0.410 [μm]
**Acquisition**	
Objective Lens	UPLSAPO 60XO
Objective Lens Mag.	60×
Objective Lens NA	1.35
Scan Device	Galvano
Scan Direction	One way
Sampling Speed	2.0 [μs/pixel]
Sequential Mode	Line
Integration Type	None
Integration Count	0
Zoom	×2.38
**GFP channel settings**	
Emission WaveLength	510 [nm]
PMT Voltage	500 [V]
C.A.	200 [μm]
Bits/Pixel	12 [bits]
Laser Wavelength	488 [nm]
Laser Transmissivity	0.05 [%]
AOTF/AOM Transmissivity	0.5 [%]
Laser ND Filter	10 [%]
Detection Wavelength	500 – 600 [nm]
**DIC channel settings**	
PMT Voltage	380 [V]
C.A.	200 [μm]
Bits/Pixel	12 [bits]
Laser Wavelength	488 [nm]
Laser Transmissivity	0.05 [%]
AOTF/AOM Transmissivity	0.5 [%]
Laser ND Filter	10 [%]
**Live cell imaging**	
Interval	30 seconds
Duration	2 hours
FoV	50 μm × 50 μm

Appendix Table S4. Cryo-ET imaging details.

**Sample preparation**	
EM grids	Cryosections: continuous carbon<br>Lamellae: gold
Plunge freezer	Vitrobot Mark IV
Cryomicrotome	UC7/FC7
Attachment device	Crion
Micromanipulators	Leica micromanipulator; Narishige MN-151-S
Cryomicrotome feed	100 nm
Cryo-FIB-SEM	Helios NanoLab 650 DualBeam
Cryo-transfer device	Quorum PP2000T
Milling currents	Rough: 2.8 nA<br>Intermediate: 0.28 nA<br>Polishing: 48 pA
**Cryo-ET data collection**	
Microscope	Titan Krios
Energy	300 keV
Camera: recording mode	FII: integration<br>K3: super-resolution movie frames
Energy filter width	K3: 20 eV
Tomography software	TFS Tomo4 and SerialEM
Unbinned pixel size	FII: 7.3, 12.4Å<br>K3: 3.4, 4.5 Å
Contrast mechanism	Defocus phase contrast (cryolamellae, cryosections, oligonucleosomes)<br>Volta phase contrast (cryolamellae, cryosections)
Defocus (nominal)	Defocus phase contrast: −5 (K3) or −10 μm (FII)<br>Volta phase contrast: −0.5 μm
Cumulative dose	cryosections: 100 e^- / $Å^2$<br>oligonucleosomes: 120 e^- / $Å^2$<br>cryolamellae: 110 e^- / $Å^2$
Dose fractionation	cryosections: 1/cosine<br>oligonucleosomes and cryolamellae: (1/cosine)^(1/y), where y = 4
Tilt range	cryosections: ±60°; bidirectional, negative angles first<br>cryolamellae: −70° to +42° start −14°, dose-symmetric<br>oligonucleosomes: ±60° start 0°; dose-symmetric, negative angles first
Tilt increment	2°
**Cryo-ET data analysis**	
Tomogram processing	IMOD 4.11
Template matching	PEET 1.15
Reference creation	Bsoft 1.8.8
Mask creation	Bsoft 1.8.8, RELION 3.0.8
Subtomogram analysis	RELION 3.0.8, Auxiliary scripts
Tomogram visualization	UCSF Chimera 1.15, IMOD 4.11
Auxiliary scripts	github.com/anaphaze/ot-tools
Calculations	Google sheets, FIJI

Figure/movie editing	Adobe Photoshop, Illustrator, and Premiere Pro, www.biobender.com

Appendix Table S5. Cryotomogram details.

Tomogram	Description	Sample	Fig	Dose (e/Å^2)	Pixel size (Å)	Δf * (μm)	Cam	VPP	thick (nm)	resid
20161115_08	M	section	S2A,C	95	12.4	11.1	FII	−	160	0.56
20180528_02	M	section	S2B,D	100	7.3	0.5	FII	+	170	0.86
20221029_03	G1, 9% DMSO	lamella	2	110	3.4	0.5	K3	+	70	0.49
20221028_39	G1, 9% DMSO	lamella	2	110	3.4	0.5	K3	+	125	0.57
20221028_14	G1, 9% DMSO	lamella	3, 6, EV4, Mov EV2	110	3.4	0.5	K3	+	80	0.57
20221028_[10, 18, 19, 21, 23, 24, 29, 30, 41] 20221029_[20, 32, 40, 49, 51, 53] 20221030_[36, 41]	G1, 9% DMSO	lamella	-	110	3.4	0.5	K3	+	70–150	0.31–1.40
20220809_05	M, 9% DMSO	lamella	4 – 6	110	3.4	0.5	K3	+	150	0.31
20230209_28	M, 9% DMSO	lamella	4, Mov EV4	110	3.4	0.5	K3	+	110	0.56
20230209_[42, 58]	M, 9% DMSO	lamella	-	110	3.4	0.5	K3	+	120, 80	0.85, 0.90
20201217_01	G1, 0% DMSO	lamella	S4A	120	3.4	7.0	K3	−	140	0.76
20210110_20	G1, 3% DMSO	lamella	S4B	110	3.4	7.8	K3	−	190	0.20
20210303_16	G1, 6% DMSO	lamella	S4C	110	3.4	8.4	K3	−	200	0.52
20210303_02	G1, 9% DMSO	lamella	S4D	110	3.4	8.2	K3	−	110	0.79
20210423_17	M, 9% DMSO	lamella	S5A	110	3.4	4.9	K3	−	150	1.29
20210526_01	M, 9% Glycerol	lamella	S5B	110	3.4	4.2	K3	−	190	0.54
20210923_03	M, 9% DMSO	lamella	S5C	110	3.4	0.5	K3	+	110	0.91
20211101_16	M, 9% Glycerol	lamella	S5D	110	3.4	0.5	K3	+	180	0.73
20210916_03	Oligo	plunge	S6A	120	3.4	0.5	K3	+	75	0.27
20210904_26	Oligo, 9% DMSO	plunge	S6B	120	3.4	0.5	K3	+	90	0.41
20221028_31	G1, 9% DMSO	lamella	S9	110	3.4	0.5	K3	+	210	0.31
20210923_09	M, 9% DMSO	lamella	S25	110	3.4	0.5	K3	+	180	0.41
20210916_[05, 06, 07, 09–33]	Oligo	plunge	-	120	3.4	0.5	K3	+	~90	0.27–1.04
20210904_[12–41}]	Oligo, 9% DMSO	plunge	-	120	3.4	0.5	K3	+	~90	0.35–1.39
20240101_[29, 53]	G1, 9% Glycerol	lamella	S14	110	3.4	0.5	K3	+	~90, 70	0.26, 0.29
20240101_[21, 31, 44]	G1, 9% Glycerol	lamella	-	110	3.4	0.5	K3	+	100–120	0.29–0.62
20231207_[25, 27]	G1	lamella	S17	110	3.4	0.5	K3	+	90, 125	0.27, 0.30
20231207_[33, 37, 38, 40, 42], 20231215_[24, 26]	G1	lamella	-	110	3.4	0.5	K3	+	85–180	0.28–0.52

The tilt increment was 2° for all sets. All data reported in this table were used for subtomogram analysis and were deposited as EMPIAR-12425. Treatment/State: G1 = G1 phase; M = metaphase; Oligo = HeLa oligonucleosomes; plunge = plunge frozen. Dose, in electrons / Å^2. Pixel size is the unbinned pixel size as the specimen level. The K3 data were collected in super-resolution mode, with ½ the pixel size reported in the table. Pixel size therefore refers to the camera's "bin ×1" pixel. Refined defocus (Δf) values are reported for defocus phase-contrast data. Nominal defoci are reported for Volta phase-contrast (VPP) data. Δtilt = tilt increment. Fig = figures that show this dataset; those without a figure number were used for subtomogram averaging. Camera (Cam): FII = Falcon II, K3 = K3-GIF. thick = thickness, measured from the reconstructed cryotomogram. resid = alignment residual, in nanometers.

Appendix Table S6. Subtomogram analysis of chromatin.

	Oligonucleosomes		G1 phase lamella		Metaphase lamella	
	0% DMSO	9% DMSO	NCP Class 1 – 7	Di-NCP	NCP Class 1 – 8	Di-NCP
**Template matching**						
Tomograms	29	30	21		4	
Reference	Cylinder	Cylinder	Cylinder		Cylinder	
Mask	Cylinder	Cylinder	Cylinder		Cylinder	
**2-D classification**						
Subtomograms	1,136,628	1,280,772	-		-	
Classes (total)	50	50	-		-	
Classes (kept)	34	27	-		-	
T parameter	2	2	-		-	
Mask (Å)	110	110	-		-	
E-step (Å)	25	25	-		-	
**3-D classification**						
Subtomograms	207,884	175,984	165,964		145,804	
Reference	Cylinder	Cylinder	Di-NCP STA (G1)		Di-NCP STA (Metaphase)	
Mask, sphere (Å)	110	110	240		240	
E-step (Å)	20	20	20		20	
T parameter	4	4	4		4	
Classes (total)	30	30	50		50	
Classes (kept)	2	3	3	1	4	1
Symmetry imposed	C1	C1	C1		C1	
**Refinement**						
Subtomograms	8,124	8,119	19,489	4,199	17,224	4,668
Reference	Cylinder	Cylinder	NCP Class 1 – 7 STA (G1)	Di-NCP STA (G1)	NCP Class 1 – 8 STA (Metaphase)	Di-NCP STA (Metaphase)
Mask, sphere (Å)	110	110	240	240	240	240
Symmetry imposed	C1	C1	C1	C1	C1	C1
Resolution (Å)						
FSC = 0.5	24.2	24.0	30.6 – 33.7	26.8	32.5 – 34.3	26.3
FSC = 0.143	19.6	20.5	24.3 – 27.4	21.1	24.9 – 26.2	21.3
EMDB entry	EMD-37004	EMD-37005	EMD-36993	EMD-36992	EMD-36999	EMD-36998

NCP = mononucleosomes, Di-NCP = ordered stacked dinucleosomes, STA = subtomogram average. The values here reflect the final major round of 3-D classification (Appendix Figs S12D and S26D) and refinement (Appendix Figs S13 & S27, A & B).

Appendix Table S7. Subtomogram analysis of chromatin – nucleosomes with gyre-proximal densities.

	G1 phase lamella					Metaphase lamella			
	Prox. 1	Prox. 2	Prox. 3	Prox. 4	Prox. 5	Prox. 1	Prox. 2	Prox. 3	Prox. 4
**Template matching**									
Tomograms			21				4		
Reference			Cylinder				Cylinder		
Mask			Cylinder				Cylinder		
**2-D classification**									
Subtomograms			-				-		
Classes (total)			-				-		
Classes (kept)			-				-		
T parameter			-				-		
Mask (Å)			-				-		
E-step (Å)			-				-		
**3-D classification**									
Subtomograms			165,964				145,804		
Reference			Di-NCP STA (G1)				Di-NCP STA (Metaphase)		
Mask, sphere (Å)			240				240		
E-step (Å)			20				20		
T parameter			4				4		
Classes (total)			50				50		
Classes (kept)			1				1		
Symmetry imposed			C1				C1		
**Refinement**									
Subtomograms	2,016	1,309	1,947	2,126	2,038	1,361	1,837	1,725	1,397
Reference	Prox. 1 STA (G1)	Prox. 2 STA (G1)	Prox. 3 STA (G1)	Prox. 4 STA (G1)	Prox. 5 STA (G1)	Prox. 1 STA (Metaphase)	Prox. 2 STA (Metaphase)	Prox. 3 STA (Metaphase)	Prox. 4 STA (Metaphase)
Mask, sphere (Å)	240	240	240	240	240	240	240	240	240
Symmetry imposed	C1	C1	C1	C1	C1	C1	C1	C1	C1
Resolution (Å)									
FSC = 0.5	30.6	33.4	31.3	33.0	33.7	32.5	32.6	33.4	34.3
FSC = 0.143	24.3	25.5	25.3	27.4	26.4	25.6	24.9	25.9	26.2
EMDB entry			EMD-36994				EMD-37000		

Prox. = Nucleosomes with gyre-proximal density, STA = subtomogram average. The values here reflect the final major round of 3-D classification (Appendix Figs S12D and S26D) and refinement (Appendix Figs S13C and S27C).

Appendix Table S8. Subtomogram analysis of G1 chromatin with 9% glycerol or without cryoprotectant.

	G1 with 9% glycerol cryoprotectant		G1 without cryoprotectant
	NCP Class 1 – 6	Prox.	NCP Class 1 – 9
**Template matching**			
Tomograms	5		9
Reference	Cylinder		Cylinder
Mask	Cylinder		Cylinder
**2-D classification**			
Subtomograms	-		-
Classes (total)	-		-
Classes (kept)	-		-
T parameter	-		-
Mask (Å)	-		-
E-step (Å)	-		-
**3-D classification**			
Subtomograms	187,245		175,229
Reference	NCP STA (G1)		NCP STA (Metaphase)
Mask, sphere (Å)	240		240
E-step (Å)	20		20
T parameter	4		4
Classes (total)	50		50
Classes (kept)	3	1	4
Symmetry imposed	C1		C1
**Refinement**			
Subtomograms	12,607	1,670	17,312
Reference	NCP Class 1 – 6 STA	Prox. STA	NCP Class 1 – 9 STA
Mask, sphere (Å)	240	240	240
Symmetry imposed	C1	C1	C1
Resolution (Å)			
FSC = 0.5	28.8 – 34.0	31.4	29.4 – 55.7
FSC = 0.143	24.3 – 28.6	26.0	25.9 – 31.3
EMDB entry	EMD-62351	EMD-62352	EMD-62350

NCP = mononucleosomes, Prox. = Nucleosomes with gyre-proximal density. The values here reflect the final major round of 3-D classification and refinement shown in Appendix Figs S15 & S16 (9% glycerol) and Appendix Figs S18 & S19 (no cryoprotectant).

Appendix Table S9. Subtomogram analysis of megacomplexes.

	G1 phase lamella			Metaphase lamella
	Ribosome (Cytoplasm only)	Ribosome (Full volume)	Preribosome (Full volume)	Ribosome
**Template matching**				
Tomograms	11	21		4
Reference	Sphere	Sphere		Sphere
Mask	Sphere	Sphere		Sphere
**2-D classification**				
Subtomograms	-	192,309		-
Classes (total)	-	100		-
Classes (kept)	-	15		-
T parameter	-	2		-
Mask (Å)	-	300		-
E-step (Å)	-	20		-
**3-D classification**				
Subtomograms	11,972	25,886		48,849
Reference	Sphere	Ribosome STA		Ribosome STA
Mask, sphere (Å)	300	300		300
E-step (Å)	50	50		50
T parameter	4	4		4
Classes (total)	30	30		50
Classes (kept)	1	1	1	1
Symmetry imposed	C1	C1		C1
**Refinement**				
Subtomograms	684	702	635	332
Reference	Ribosome STA	Ribosome STA	Preribosome STA	Ribosome STA
Mask, sphere (Å)	300	300	300	300
Symmetry imposed	C1	C1	C1	C1
Resolution (Å)				
FSC = 0.5	31.7	33.4	33.3	36.8
FSC = 0.143	24.7	24.7	25.4	26.9
EMDB entry	EMD-37002		EMD-37001	EMD-37003

STA = subtomogram average.

Appendix discussion

Optimization of cryoprotectants for cellular cryo-ET

In cellular cryo-EM and cryo-ET experiments, cells are frozen and imaged in conditions that immobilize the water molecules in a glassy "vitreous ice" state that resembles the life-like disordered form (Dubochet *et al*, 1988). Cryo-EM images of intact eukaryotic cells have poor contrast because cells are generally too thick. Therefore, frozen-hydrated cellular samples are made much thinner by either cryosectioning (Ladinsky *et al*, 2006; Studer *et al*, 2014) or cryo-FIB milling (Hayles *et al*, 2007; Marko *et al*, 2006; Medeiros *et al*, 2018; Rigort *et al*, 2010; Schaffer *et al*, 2015; Villa *et al*, 2013). We did pilot cryo-ET experiments on metaphase RPE-1 cells prepared by sequential thymidine block and Taxol-induced arrest (before the Scott protocol was published). These cells were then self-pressurized frozen, which is a method we have previously used to prepare frozen-hydrated yeast for cryosections (Cai *et al*, 2018b; Ma *et al*, 2022; Ng *et al*, 2019; Ng *et al*, 2020), with dextran as an extracellular cryoprotectant. Compared to standard high-pressure freezing, self-pressurized freezing is faster and cheaper (Han *et al*, 2012; Yakovlev & Downing, 2011). In this method, a cellular sample is sealed in a copper tube, then submerged in liquid-ethane cryogen. The cellular material that is closest to the tube's inner-wall surface forms crystalline ice. This ice expands and pressurizes the material that is cooling deeper inside the tube, suppressing ice-crystal formation there. Unfortunately, our pilot samples showed diffraction-contrast features (Dubochet *et al.*, 1988) in the tilt-series projection images (Appendix Fig S2, A and B), indicating that the ice within the frozen cells was crystalline instead of vitreous. Furthermore, the crevasse artifacts were more severe and the cryosections were more often detached from the carbon support in our RPE-1 samples than in our yeast ones. In the cryotomograms, compacted chromosomes have the expected dimensions (hundreds of nanometers) and are surrounded by ribosomes (Appendix Fig S2, C and D), as previously observed *in situ* (Eltsov *et al*, 2008; McDowall *et al*, 1986). Chromatin densities in our pilot samples appeared string-like, rather than granular, which may be an artifact from the crystalline ice. While these experiments show that our RPE-1 cryosections are inadequate as cryo-ET samples, they suggest that mitotic chromosomes can potentially be located due to different image features relative to the surrounding cytoplasm.

An alternative and more-popular way to prepare frozen-hydrated cells is plunge freezing. While the region of human cells at the nuclear periphery is just thin enough (~5 to 10 μm) to be plunge-frozen in unmodified growth medium (medium) in a vitreous state (Cai *et al*, 2018a; Mahamid *et al*, 2016), both metaphase cells and the regions in the nuclei interior are thicker than this limit. Ice-crystal formation in thick plunge-frozen biological cryo-ET samples can be suppressed by prior treatment with cryoprotectants (Bäuerlein *et al*, 2022; Cai *et al*, 2022; Creekmore *et al*, 2024; Glynn *et al*, 2024; Harapin *et al*, 2015; Jentoft *et al*, 2023). Glycerol and DMSO are popular cryoprotectants used to prepare cell-line cryostocks, so we treated cells with each of these cryoprotectants at different concentrations and then characterized their morphology by timelapse light microscopy (Appendix Fig S3A). RPE-1 cells that were incubated with 6% and higher concentrations of DMSO, but not glycerol, showed detachment from the tissue culture plate and shrinkage of the nucleus after a 6-minute treatment (Appendix Fig S3, B and C). At the highest concentration (9%) of DMSO and glycerol tested, we found that DMSO-treated cells start detaching in 2 minutes. Therefore, at the highest – and most cryoprotective – concentrations of DMSO, incubations need to be shorter than 2 minutes, which was done for all following experiments. While this cryoprotectant screen suggests that glycerol is better than DMSO, the experiments below show that DMSO treatment allows for higher image contrast and that the treatment time can be shortened to a few seconds.

To determine the effects of cryoprotectants on ice-crystal formation and image contrast, we performed cryo-ET of cryo-FIB-milled cryolamellae of G1 cells that had been treated with different concentrations of DMSO and glycerol. Due to the time-consuming nature of these tests, we used G1 cells, which are easier to prepare, and we used DMSO to screen the cryoprotectant concentration. Grids bearing the G1 RPE-1 cells were briefly immersed in PBS+Ca+Mg plus cryoprotectant, then immediately mounted in the Vitrobot, blotted manually and then plunged into propane-ethane mix (details in Methods). Because cryoprotection is expected to change with sample thickness, we kept our analysis to regions of approximate equal thickness, namely the nuclear periphery, which could be located by the distinctive double membranes of the nuclear envelope. Tilt series of these samples show that ice-crystal diffraction contrast occupied large regions in samples treated with ≤ 3% cryoprotectant, smaller regions in 6% cryoprotectant (Appendix Fig S4), and were undetectable in most samples in 9% cryoprotectant.

Next, we collected cryo-ET data of cells in 9% glycerol for comparison with 9% DMSO, in both defocus phase contrast and with Volta phase plate (VPP). VPP data have more low-resolution contrast than defocus phase-contrast data (Danev *et al*, 2014; Fukuda *et al*, 2015). This extra contrast facilitates the detection of macromolecular complexes in challenging samples like cryotomograms of cells, especially if contrast is further lowered by cryoprotectant. We found that cells treated with 9% glycerol produce cryotomograms with unacceptably low contrast, even when the VPP is used (Appendix Fig S5). Therefore, a brief 9% DMSO treatment offers the best combination of cryoprotection and contrast. In all subsequently collected tilt series, only those without diffraction-contrast artifacts were used for further analysis.

DMSO has been shown to affect the stability of some protein complexes *in vitro* (Chan *et al*, 2017). To control for the potential disruption of nucleosomes in 9% DMSO, we plunge-froze HeLa oligonucleosomes in either pure storage buffer or storage buffer plus 9% DMSO, followed by cryo-ET with a VPP. Cryotomogram densities showed the characteristic beads-on-string motifs are preserved in 9% DMSO (Appendix Fig S6). Nucleosomes have a conspicuous double-gyre motif (two 10-nm-long parallel linear densities) in cryotomographic slices (Appendix Fig S6, insets). This double-gyre motif is evident when the nucleosome is oriented with its superhelical axis parallel to the ice surface, and corresponds to side and gyre views (Zhou *et al*, 2019). These observations suggest that the 9% DMSO treatment does not disrupt nucleosomes.

To further test if DMSO treatment alters nucleosome structure, we performed subtomogram analysis of the oligonucleosome samples (details in Methods). This workflow was originally established using nuclear lysates (Cai *et al*, 2018c) and has three major steps. First, nucleosome-like particles are template-matched using a rounded-cylinder reference. Template matching done this way is analogous to single-particle cryo-EM automated particle picking using a Gaussian (2-D) blob that has dimensions similar to the complex under study. Given the simplistic nature of the reference, template-matching hits are not considered to be “identified” nucleosomes, so we refer to them as candidate nucleosomes. Second, canonical nucleosomes are identified based on their structure by sequential 2-D and then 3-D subtomogram classification. Alternatively, the subtomograms are 3-D classified directly (2-D classification skipped), resulting in more nucleosomes detected, though at a cost of computation time (Tan *et al*, 2023). Third, the subtomogram class averages are inspected for known structures, which are subsequently refined in 3-D. Canonical nucleosome class averages were detected in both the absence of DMSO (Appendix Fig S7A) and presence of 9% DMSO (Appendix Fig S7B). Refinement of the canonical nucleosome classes that have the highest-resolution features

resulted in a 19 – 19.5 Å resolution subtomogram averages; both classes resemble the canonical nucleosome with some ordered linker DNA as expected of oligonucleosome samples.

DMSO – and also glycerol – may also have unexpected effects on cellular health. Immunofluorescence of G1 and metaphase cells treated with 9% DMSO or 9% glycerol show a similar CTD-S5P (Fig 1, B and C) and CTD-S2P (Appendix Fig S8) distributions compared to untreated cells. These confocal microscopy data suggest that large-scale chromatin structure is not disrupted by these cryoprotectant treatments. To control for the induction of apoptosis, we performed immunoblot analysis for the apoptotic marker, cleaved poly (ADP-ribose) polymerase-1 (PARP-1) (Kaufmann, 1989; Kaufmann *et al*, 1993). Untreated negative control G1 and metaphase cells did not show cleaved PARP-1 while positive-control cells treated with the apoptosis inducer staurosporine (Bertrand *et al*, 1994) showed cleaved PARP-1 (Appendix Fig S9). Neither DMSO nor glycerol treatment induced cleaved PARP-1 (Appendix Fig S9), indicating that brief cryoprotectant treatments with these cryoprotectants did not induce apoptosis. In summary, brief immersion in medium containing 9% DMSO both cryoprotects plunge-frozen cells without killing them and preserves nucleosome structure. In the cryo-ET experiments described below, most samples were cryoprotected in medium with 9% DMSO, with additional control samples prepared in medium with either no cryoprotectant or 9% glycerol.

Subtomogram averaging considerations

To get insight into the nucleosome organization of G1 cells, we performed subtomogram analysis using a cylindrical reference, which minimizes model bias. We sped up this initial analysis by doing template matching with a larger grid spacing of 21 nm. The expectation is that a subset of nucleosomes will be missed, which is acceptable as long as the analysis provides a representative sample of structures within this large dataset. We analyzed the chromatin domains by direct 3-D classification of the template-matched candidate nucleosomes. To limit the effects of neighboring particles in the alignment and classification, we used a tight 110 Å diameter spherical mask. As a positive control, we restricted subtomogram analysis to the chromatin domains. We found canonical nucleosome class averages (Appendix Fig S11A) like we saw in HeLa (Cai *et al.*, 2018a). These class averages have the unmistakable features of canonical nucleosomes: a ~10-nm-long double-gyre density that follows a left-handed ~1.65 turn helical path. Next, we performed a negative-control subtomogram analysis of the cytoplasm, which does not have chromatin. As expected, the cytoplasm does not have any nucleosome class averages (Appendix Fig S11B).

To increase the number of detectable nucleosomes, we repeated the template matching with a smaller 7 nm grid spacing. Three-dimensional classification of the resultant hits revealed 4 canonical-nucleosome class averages (Appendix Fig S12A). Two of these class averages had an extra density near one face, suggesting the presence of a nearby complex that was potentially excluded by the tight spherical mask. Because cryotomographic slices had densities that resemble stacked nucleosomes (Fig 2, B and D), we repeated 3-D classification, except using a stacked-double-cylinder reference and a mask large enough (240 Å sphere) to accommodate two nucleosomes (Appendix Fig S12B). Classification with these new parameters revealed two classes that each had a canonical nucleosome and an extra density near the face.

APPENDIX REFERENCES

Bäuerlein FJB, Pastor-Pareja JC, Fernández-Busnadiego R (2022) Cryo-electron tomography of native Drosophila tissues vitrified by plunge freezing. *bioRxiv*

Bertrand R, Solary E, O'Connor P, Kohn KW, Pommier Y (1994) Induction of a common pathway of apoptosis by staurosporine. *Exp Cell Res* 211: 314-321

Buchholz TO, Krull A, Shahidi R, Pigino G, Jekely G, Jug F (2019) Content-aware image restoration for electron microscopy. *Methods Cell Biol* 152: 277-289

Cai S, Böck D, Pilhofer M, Gan L (2018a) The in situ structures of mono-, di-, and trinucleosomes in human heterochromatin. *Mol Biol Cell* 29: 2450-2457

Cai S, Chen C, Tan ZY, Huang Y, Shi J, Gan L (2018b) Cryo-ET reveals the macromolecular reorganization of S. pombe mitotic chromosomes in vivo. *Proc Natl Acad Sci U S A* 115: 10977-10982

Cai S, Song Y, Chen C, Shi J, Gan L (2018c) Natural chromatin is heterogeneous and self-associates in vitro. *Mol Biol Cell* 29: 1652-1663

Cai S, Wu Y, Guillen-Samander A, Hancock-Cerutti W, Liu J, De Camilli P (2022) In situ architecture of the lipid transport protein VPS13C at ER-lysosome membrane contacts. *Proc Natl Acad Sci U S A* 119: e2203769119

Chan DS, Kavanagh ME, McLean KJ, Munro AW, Matak-Vinkovic D, Coyne AG, Abell C (2017) Effect of DMSO on Protein Structure and Interactions Assessed by Collision-Induced Dissociation and Unfolding. *Anal Chem* 89: 9976-9983

Chen M, Dai W, Sun SY, Jonasch D, He CY, Schmid MF, Chiu W, Ludtke SJ (2017) Convolutional neural networks for automated annotation of cellular cryo-electron tomograms. *Nature methods* 14: 983-985

Creekmore BC, Kixmoeller K, Black BE, Lee EB, Chang YW (2024) Ultrastructure of human brain tissue vitrified from autopsy revealed by cryo-ET with cryo-plasma FIB milling. *Nat Commun* 15: 2660

Danev R, Buijsse B, Khoshouei M, Plitzko JM, Baumeister W (2014) Volta potential phase plate for in-focus phase contrast transmission electron microscopy. *Proc Natl Acad Sci U S A* 111: 15635-15640

Dubochet J, Adrian M, Chang JJ, Homo JC, Lepault J, McDowall AW, Schultz P (1988) Cryo-electron microscopy of vitrified specimens. *Quarterly reviews of biophysics* 21: 129-228

Eltsov M, Maclellan KM, Maeshima K, Frangakis AS, Dubochet J (2008) Analysis of cryo-electron microscopy images does not support the existence of 30-nm chromatin fibers in mitotic chromosomes in situ. *Proc Natl Acad Sci U S A* 105: 19732-19737

Fukuda Y, Laugks U, Lucic V, Baumeister W, Danev R (2015) Electron cryotomography of vitrified cells with a Volta phase plate. *J Struct Biol* 190: 143-154

Glynn C, Smith JLR, Case M, Csöndör R, Katsini A, Sanita ME, Glen TS, Pennington A, Grange M (2024) Charting the molecular landscape of neuronal organisation within the hippocampus using cryo electron tomography. *bioRxiv*

Han HM, Huebinger J, Grabenbauer M (2012) Self-pressurized rapid freezing (SPRF) as a simple fixation method for cryo-electron microscopy of vitreous sections. *J Struct Biol* 178: 84-87

Harapin J, Bormel M, Sapra KT, Brunner D, Kaech A, Medalia O (2015) Structural analysis of multicellular organisms with cryo-electron tomography. *Nature methods* 12: 634-636

Hayles MF, Stokes DJ, Phifer D, Findlay KC (2007) A technique for improved focused ion beam milling of cryo-prepared life science specimens. *J Microsc* 226: 263-269

Heymann JB, Belnap DM (2007) Bsoft: image processing and molecular modeling for electron microscopy. *J Struct Biol* 157: 3-18

Jentoft IMA, Bauerlein FJB, Welp LM, Cooper BH, Petrovic A, So C, Penir SM, Politi AZ, Horokhovskyi Y, Takala I *et al* (2023) Mammalian oocytes store proteins for the early embryo on cytoplasmic lattices. *Cell* 186: 5308-5327 e5325

Kaufmann SH (1989) Induction of endonucleolytic DNA cleavage in human acute myelogenous leukemia cells by etoposide, camptothecin, and other cytotoxic anticancer drugs: a cautionary note. *Cancer Res* 49: 5870-5878

Kaufmann SH, Desnoyers S, Ottaviano Y, Davidson NE, Poirier GG (1993) Specific proteolytic cleavage of poly(ADP-ribose) polymerase: an early marker of chemotherapy-induced apoptosis. *Cancer Res* 53: 3976-3985

Khatter H, Myasnikov AG, Natchiar SK, Klaholz BP (2015) Structure of the human 80S ribosome. *Nature* 520: 640-645

Ladinsky MS, Pierson JM, McIntosh JR (2006) Vitreous cryo-sectioning of cells facilitated by a micromanipulator. *J Microsc* 224: 129-134

Ma OX, Chong WG, Lee JKE, Cai S, Siebert CA, Howe A, Zhang P, Shi J, Surana U, Gan L (2022) Cryo-ET detects bundled triple helices but not ladders in meiotic budding yeast. *PLoS One* 17: e0266035

Mahamid J, Pfeffer S, Schaffer M, Villa E, Danev R, Cuellar LK, Forster F, Hyman AA, Plitzko JM, Baumeister W (2016) Visualizing the molecular sociology at the HeLa cell nuclear periphery. *Science* 351: 969-972

Marko M, Hsieh C, Moberlychan W, Mannella CA, Frank J (2006) Focused ion beam milling of vitreous water: prospects for an alternative to cryo-ultramicrotomy of frozen-hydrated biological samples. *J Microsc* 222: 42-47

Mastronarde DN (1997) Dual-axis tomography: an approach with alignment methods that preserve resolution. *J Struct Biol* 120: 343-352

Mastronarde DN (2005) Automated electron microscope tomography using robust prediction of specimen movements. *J Struct Biol* 152: 36-51

McDowall AW, Smith JM, Dubochet J (1986) Cryo-electron microscopy of vitrified chromosomes in situ. *EMBO J* 5: 1395-1402

Medeiros JM, Bock D, Weiss GL, Kooger R, Wepf RA, Pilhofer M (2018) Robust workflow and instrumentation for cryo-focused ion beam milling of samples for electron cryotomography. *Ultramicroscopy* 190: 1-11

Ng CT, Deng L, Chen C, Lim HH, Shi J, Surana U, Gan L (2019) Electron cryotomography analysis of Dam1C/DASH at the kinetochore-spindle interface in situ. *J Cell Biol* 218: 455–473

Ng CT, Ladinsky MS, Gan L (2020) Serial Cryomicrotomy of Saccharomyces cerevisiae for Serial Electron Cryotomography. *Bio Protoc* 10: e3831

Nicastro D, Schwartz C, Pierson J, Gaudette R, Porter ME, McIntosh JR (2006) The molecular architecture of axonemes revealed by cryoelectron tomography. *Science* 313: 944-948

Pettersen EF, Goddard TD, Huang CC, Couch GS, Greenblatt DM, Meng EC, Ferrin TE (2004) UCSF Chimera--a visualization system for exploratory research and analysis. *J Comput Chem* 25: 1605-1612

Rigort A, Bauerlein FJ, Leis A, Gruska M, Hoffmann C, Laugks T, Bohm U, Eibauer M, Gnaegi H, Baumeister W *et al* (2010) Micromachining tools and correlative approaches for cellular cryo-electron tomography. *J Struct Biol* 172: 169-179

Schaffer M, Engel BD, Laugks T, Mahamid J, Plitzko JM, Baumeister W (2015) Cryo-focused Ion Beam Sample Preparation for Imaging Vitreous Cells by Cryo-electron Tomography. *Bio Protoc* 5

Scheres SH (2012) RELION: implementation of a Bayesian approach to cryo-EM structure determination. *J Struct Biol* 180: 519-530

Schindelin J, Arganda-Carreras I, Frise E, Kaynig V, Longair M, Pietzsch T, Preibisch S, Rueden C, Saalfeld S, Schmid B *et al* (2012) Fiji: an open-source platform for biological-image analysis. *Nature methods* 9: 676-682

Studer D, Klein A, Iacovache I, Gnaegi H, Zuber B (2014) A new tool based on two micromanipulators facilitates the handling of ultrathin cryosection ribbons. *J Struct Biol* 185: 125-128

Tan ZY, Cai S, Noble AJ, Chen JK, Shi J, Gan L (2023) Heterogeneous non-canonical nucleosomes predominate in yeast cells in situ. *Elife* 12

Villa E, Schaffer M, Plitzko JM, Baumeister W (2013) Opening windows into the cell: focused-ion-beam milling for cryo-electron tomography. *Current opinion in structural biology* 23: 771-777

Yakovlev S, Downing KH (2011) Freezing in sealed capillaries for preparation of frozen hydratedsections. *J Microsc* 244: 235-247

Zhou K, Gaullier G, Luger K (2019) Nucleosome structure and dynamics are coming of age. *Nat Struct Mol Biol* 26: 3-13
