# Supplementary material for: Nanoscale analysis of human G1 and metaphase chromatin in situ
Source: EMBO J. 2025 Mar 17;44(9):2658–94. doi: 10.1038/s44318-025-00407-2 (PMC12048539; doi:10.1038/s44318-025-00407-2)
Supplement: Supplementary file 3 — Movie EV1 [file 44318_2025_407_MOESM3_ESM.zip › Mov_EV1_legend.docx]

**Movie EV1. Subtomogram averages of nucleosomes in G1 cell nuclei.**

Subtomogram averages of mononucleosomes, stacked dinucleosomes, and nucleosomes with a gyre-proximal density, all from G1 chromatin domains. See also Figs 3, A – C.
