# Supplementary material for: Nanoscale analysis of human G1 and metaphase chromatin in situ
Source: EMBO J. 2025 Mar 17;44(9):2658–94. doi: 10.1038/s44318-025-00407-2 (PMC12048539; doi:10.1038/s44318-025-00407-2)
Supplement: Supplementary file 4 — Movie EV2 [file 44318_2025_407_MOESM4_ESM.zip › Mov_EV2_legend.docx]

**Movie EV2. *In situ* overview of G1 cell nucleus.**

The cryotomogram is rendered as 10 nm slices. Regions highlighted in blue indicate chromatin domains that were segmented using EMAN2. The remapped model shows nucleosomes (blue) and preribosomes (light blue) in the nucleus and ribosomes (yellow) in the cytoplasm. See also Figs 3D, which is rotated 90° counterclockwise relative to this movie.
