# Supplementary material for: Nanoscale analysis of human G1 and metaphase chromatin in situ
Source: EMBO J. 2025 Mar 17;44(9):2658–94. doi: 10.1038/s44318-025-00407-2 (PMC12048539; doi:10.1038/s44318-025-00407-2)
Supplement: Supplementary file 5 — Movie EV3 [file 44318_2025_407_MOESM5_ESM.zip › Mov_EV3_legend.docx]

**Movie EV3. Subtomogram averages of nucleosomes in metaphase chromatin.**

Subtomogram averages of mononucleosomes, stacked dinucleosomes, and nucleosomes with a gyre-proximal density, all in metaphase chromatin. See also Fig 5, A – C.
