# Supplementary material for: Nanoscale analysis of human G1 and metaphase chromatin in situ
Source: EMBO J. 2025 Mar 17;44(9):2658–94. doi: 10.1038/s44318-025-00407-2 (PMC12048539; doi:10.1038/s44318-025-00407-2)
Supplement: Supplementary file 6 — Movie EV4 [file 44318_2025_407_MOESM6_ESM.zip › Mov_EV4_legend.docx]

**Movie EV4. *In situ* overview of metaphase chromatin.**

The cryotomogram is rendered as 10 nm slices. The region highlighted in blue indicates chromatin, segmented using EMAN2. The remapped model shows nucleosomes (blue) and ribosomes (yellow). See also Fig 5D, which is rotated 90° counterclockwise relative to this movie.
