# Supplementary material for: Nanoscale analysis of human G1 and metaphase chromatin in situ
Source: EMBO J. 2025 Mar 17;44(9):2658–94. doi: 10.1038/s44318-025-00407-2 (PMC12048539; doi:10.1038/s44318-025-00407-2)
Supplement: Supplementary file 7 — Expanded View Figures [file 44318_2025_407_MOESM7_ESM.pdf]

Expanded View Figures

Figure EV1. Metaphase chromosomes are depleted of elongating RNA polymerase II.

Differential interference contrast (DIC) images of representative G1 and metaphase cells that were stained to detect DNA (stained with DAPI) and immunofluorescent detection of elongating RNAPII phosphorylated at serine 2 of the RPB1 subunit's C-terminal tail heptad repeats (RPB1-S2P). (**A**) G1 and metaphase cells were incubated in DMEM/F12 medium for 1 min before fixation. (**B**) G1 and metaphase cells were incubated in DMEM/F12 medium containing 9% DMSO for 1 min before fixation. (**C**) G1 and metaphase cells were incubated in DMEM/F12 medium containing 9% glycerol for 1 min before fixation. In the middle two columns, the DAPI and immunofluorescence signals are shown in inverted contrast for clarity.

 © The Author(s)

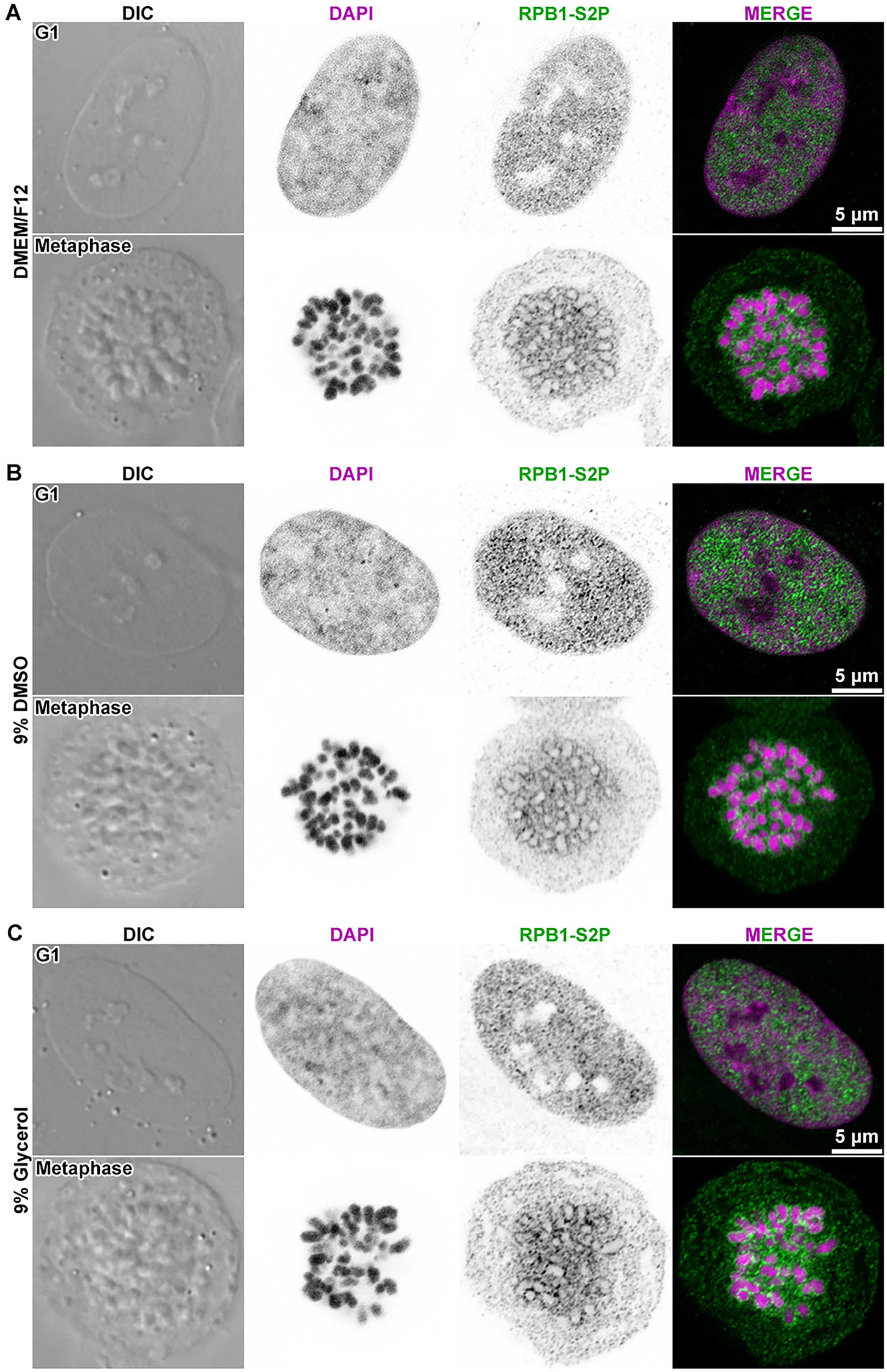
A
DIC
DAPI
RPB1-S2P
MERGE
G1
Metaphase
DMEM/F12
5 µm
B
DIC
DAPI
RPB1-S2P
MERGE
G1
Metaphase
9% DMSO
5 µm
C
DIC
DAPI
RPB1-S2P
MERGE
G1
Metaphase
9% Glycerol
5 µm

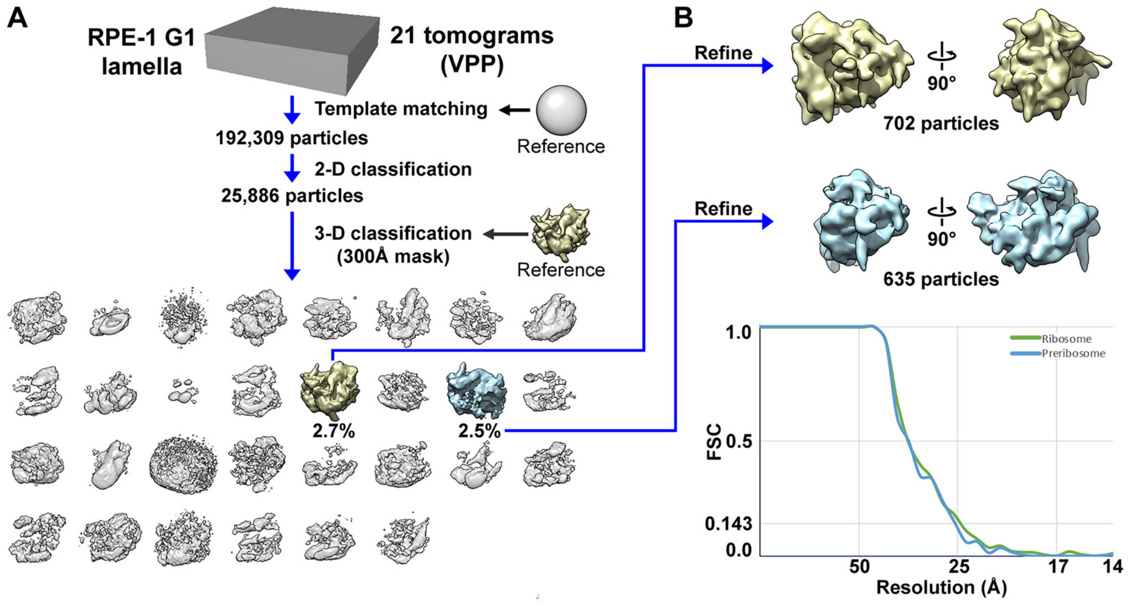

Figure EV2. Subtomogram analysis of preribosomes.

(**A**) Template matching for preribosomes was performed on all cryotomograms containing nuclear regions, using a spherical reference. The candidate hits were then subjected to 2-D classification; classes that contain subtomograms that do not correspond to large complexes were removed. The remaining subtomograms were then subjected to 3-D classification, using the ribosome refined class average shown in Appendix Fig. S10B as the reference, but low-pass filtered to 60 Å resolution. (**B**) The preribosome (blue) class average was refined to ~32 Å resolution, based on the FSC = 0.5 criterion. The 80S ribosome average (yellow) is also shown for comparison purposes. The preribosome resembles the 60S subunit of the mature ribosome and is oriented to better show their similarities.

 © The Author(s)

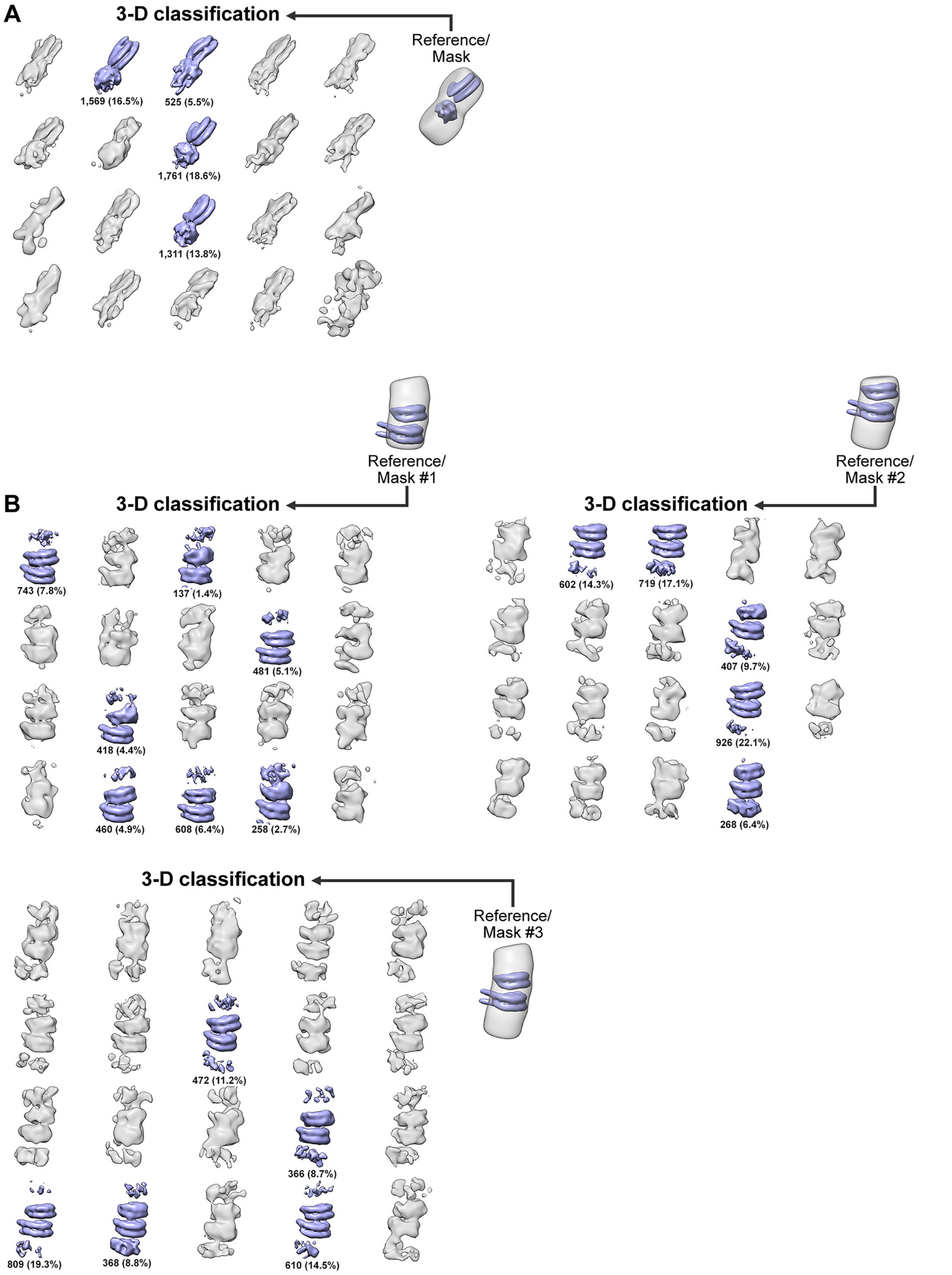
A
3-D classification
Reference/
Mask
1,569 (16.5%)
525 (5.5%)
1,761 (18.6%)
1,311 (13.8%)
B
3-D classification
Reference/
Mask #1
743 (7.8%)
137 (1.4%)
481 (5.1%)
418 (4.4%)
460 (4.9%)
608 (6.4%)
258 (2.7%)
3-D classification
Reference/
Mask #2
602 (14.3%)
719 (17.1%)
407 (9.7%)
926 (22.1%)
268 (6.4%)
3-D classification
Reference/
Mask #3
472 (11.2%)
366 (8.7%)
809 (19.3%)
368 (8.8%)
610 (14.5%)

© The Author(s)

Figure EV3. Subtomogram analysis of alternative ordered nucleosome packing motifs in G1 cells.

Nucleosome particles from Groups 2 and 3 (Appendix Fig. S13B, C, respectively) were subjected to an additional round of 3-D classification, using custom masks that enclose volumes where an additional complex may reside. Since the particles were already aligned from the previous refinement step, a restricted angular search range was imposed for these runs. The "reference/mask" models in the figure depicts the location of the volume masked-in (gray), relative to the reference (blue) used for each classification run. The masks used for these 3-D classification runs were optimized for (**A**) side-by-side nucleosomes and (**B**) ordered trinucleosomes and tetranucleosomes. Class averages that contain at least one ordered nucleosome are shaded blue.

 © The Author(s)

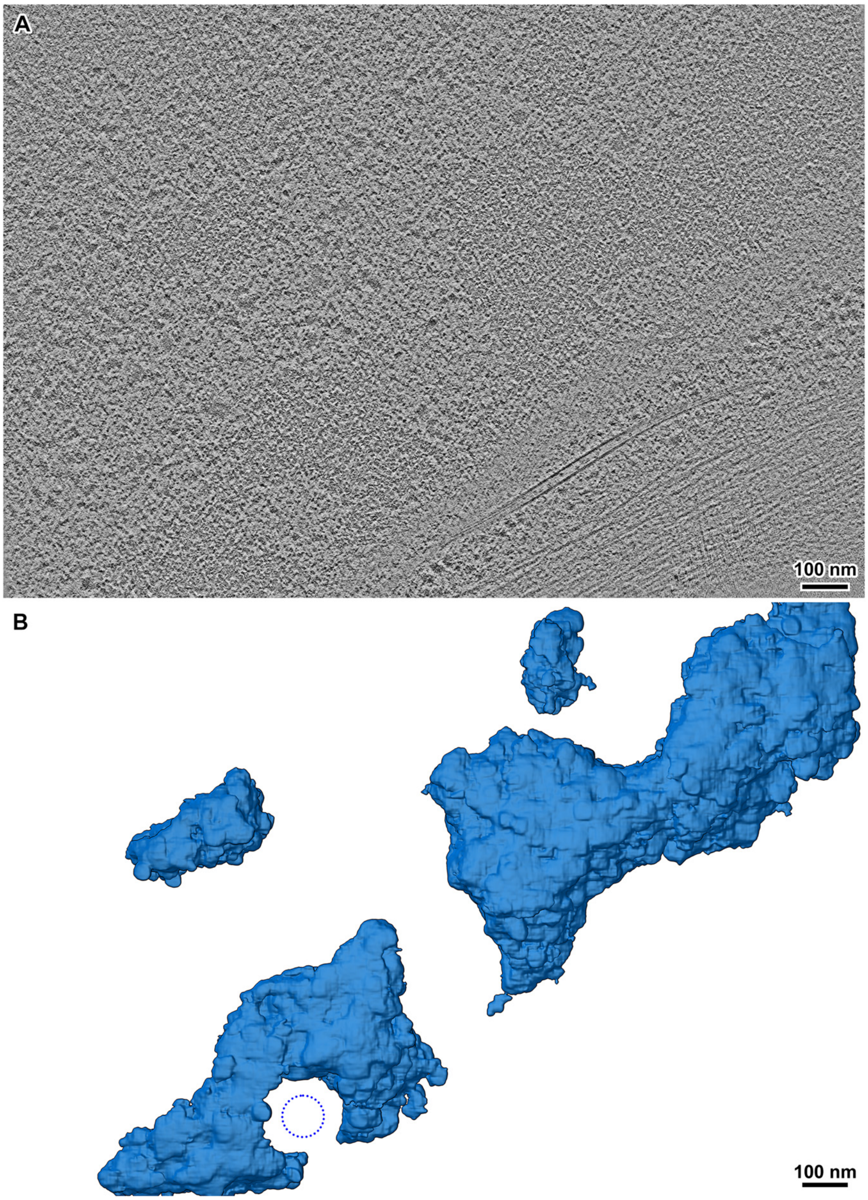

Figure EV4. G1 chromatin domains are irregular.

(**A**) Cryotomographic slice a region near the nuclear envelope. (**B**) Convolutional neural network (CNN) based segmentation of the region in (**A**). For clarity, the CNN segmentation rendering shows the entire ~90 nm thickness of the lamella whereas the cryotomographic slice shows the central 10 nm. Because of this difference, the CNN segmentation shows more chromatin than is visible in the cryotomographic slice. The dotted blue circle indicates the approximate position of the nuclear pore complex (not segmented).

© The Author(s)

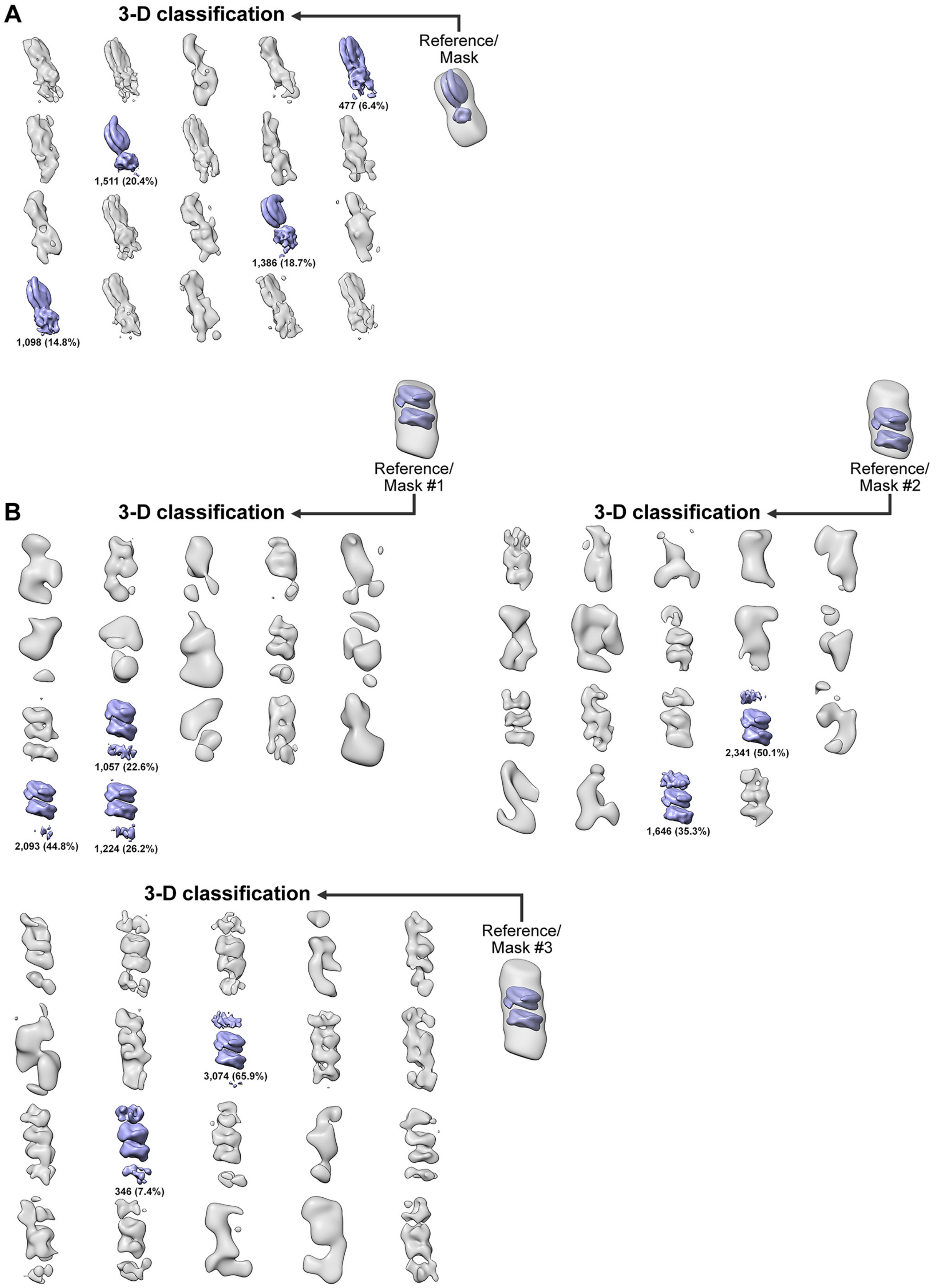
A
3-D classification
Reference/
Mask
477 (6.4%)
1,511 (20.4%)
1,386 (18.7%)
1,098 (14.8%)
B
Reference/
Mask #1
3-D classification
1,057 (22.6%)
2,093 (44.8%)
1,224 (26.2%)
Reference/
Mask #2
3-D classification
2,341 (50.1%)
1,646 (35.3%)
3-D classification
Reference/
Mask #3
3,074 (65.9%)
346 (7.4%)

 © The Author(s)

Figure EV5. Subtomogram analysis of alternative ordered nucleosome packing motifs in metaphase cells.

Similar to Fig. EV3, nucleosome particles from Groups 2 and 3 (Appendix Fig. S27B, C, respectively) were subjected to an additional round of 3-D classification, using custom masks that enclose volumes where an additional complex may reside. Since the particles were already aligned from the previous refinement step, a restricted angular search range was imposed for these runs. The "reference/mask" models in the figure depicts the location of the volume masked-in (gray), relative to the reference (blue) used for each classification run. The masks used for these 3-D classification runs were optimized for (**A**) side-by-side nucleosomes and (**B**) ordered trinucleosomes. Class averages that contain at least one ordered nucleosome are shaded blue.

© The Author(s)
